# Integrated genomics analysis highlights important SNPs and genes implicated in moderate-to-severe asthma based on GWAS and eQTL datasets

**DOI:** 10.1186/s12890-020-01303-7

**Published:** 2020-10-16

**Authors:** Zhouzhou Dong, Yunlong Ma, Hua Zhou, Linhui Shi, Gongjie Ye, Lei Yang, Panpan Liu, Li Zhou

**Affiliations:** 1 Critical Care Unit, Ningbo Medical Center Lihuili Hospital, Taipei Medical University Ningbo Medical Center, Ningbo, Zhejiang 315100 P.R. China; 2 Institute of Biomedical Big Data, Wenzhou Medical University, Wenzhou, 325027 Zhejiang China; 3 School of Biomedical Engineering, School of Ophthalmology & Optometry and Eye Hospital, Wenzhou Medical University, Wenzhou, 325027 Zhejiang China; 4 Department of Respiratory Disease, The First Affiliated Hospital, School of Medicine, Zhejiang University, Hangzhou, Zhejiang P.R. China; 5 Department of Immunology and Rheumatology, Ningbo Medical Center Lihuili Hospital, Taipei Medical University Ningbo Medical Center, Ningbo, Zhejiang 315100 P.R. China

**Keywords:** Severe asthma, Genetic variants, Gene expression, Susceptibility genes, GWAS

## Abstract

**Background:**

Severe asthma is a chronic disease contributing to disproportionate disease morbidity and mortality. From the year of 2007, many genome-wide association studies (GWAS) have documented a large number of asthma-associated genetic variants and related genes. Nevertheless, the molecular mechanism of these identified variants involved in asthma or severe asthma risk remains largely unknown.

**Methods:**

In the current study, we systematically integrated 3 independent expression quantitative trait loci (eQTL) data (*N* = 1977) and a large-scale GWAS summary data of moderate-to-severe asthma (*N* = 30,810) by using the Sherlock Bayesian analysis to identify whether expression-related variants contribute risk to severe asthma. Furthermore, we performed various bioinformatics analyses, including pathway enrichment analysis, PPI network enrichment analysis, in silico permutation analysis, DEG analysis and co-expression analysis, to prioritize important genes associated with severe asthma.

**Results:**

In the discovery stage, we identified 1129 significant genes associated with moderate-to-severe asthma by using the Sherlock Bayesian analysis. Two hundred twenty-eight genes were prominently replicated by using MAGMA gene-based analysis. These 228 replicated genes were enriched in 17 biological pathways including antigen processing and presentation (Corrected *P* = 4.30 × 10^− 6^), type I diabetes mellitus (Corrected *P* = 7.09 × 10^− 5^), and asthma (Corrected *P* = 1.72 × 10^− 3^). With the use of a series of bioinformatics analyses, we highlighted 11 important genes such as *GNGT2*, *TLR6*, and *TTC19* as authentic risk genes associated with moderate-to-severe/severe asthma. With respect to *GNGT2*, there were 3 eSNPs of rs17637472 (P_eQTL_ = 2.98 × 10^− 8^ and P_GWAS_ = 3.40 × 10^− 8^), rs11265180 (P_eQTL_ = 6.0 × 10^− 6^ and P_GWAS_ = 1.99 × 10^− 3^), and rs1867087 (P_eQTL_ = 1.0 × 10^− 4^ and P_GWAS_ = 1.84 × 10^− 5^) identified. In addition, *GNGT2* is significantly expressed in severe asthma compared with mild-moderate asthma (*P* = 0.045), and Gngt2 shows significantly distinct expression patterns between vehicle and various glucocorticoids (Anova *P* = 1.55 × 10^− 6^).

**Conclusions:**

Our current study provides multiple lines of evidence to support that these 11 identified genes as important candidates implicated in the pathogenesis of severe asthma.

## Background

Asthma is the most prevalent chronic respiratory disease which is characterized by aberrant and inflamed mucosa of the airways, wheezing and shortness of breath [1, 2]. Among these asthma patients, 10–15% have severe asthma with clinical symptoms including associated frequent severe exacerbations, dependence on high doses of inhaled or oral steroids, debilitating breathlessness, and low baseline lung function [3, 4]. Severe asthma among children and adult populations is a significant threat that is correlated with disproportionate disease morbidity and mortality. Previous twin and family studies have been reported that both environmental and genetic components convey susceptibility to asthma [5, 6]. There is estimated to vary between 35 and 95% of the heritability of asthma [5, 7]. Genetic studies offer a structured approach of identifying molecular targets for treating the syndrome and understanding the etiology of asthma [8–10].

With the development of sequencing and microarray technique, genome-wide association study (GWAS) is widely employed and thought to be an effective mean of simultaneously examining the association of tons of SNPs with traits of interest. The well-powered GWAS based on large-scale samples highly increase the likelihood to identify disease-associated genetic loci. In recent years, many GWASs on asthma were conducted, and numerous genetic loci were identified to be associated with asthma [2, 8–16]. Meanwhile, numerous genetic association studies of severe asthma were carried out, but most of these studies are underpowered so that cannot clearly differentiate asthma severity variants from these identified asthma susceptibility variants [17]. Most recently, a GWAS on asthma severity with large-scale samples (*N* = 57,695) was reported, and several asthma severity risk genes were identified; such as *MUC5AC*, *KIAA1109*, and *GATA3* [15]. Nevertheless, the molecular mechanism of how these identified genetic loci convey risk to severe asthma is still equivocal. It should be noted that these identified genetic loci from GWAS contain a plenty of useful information, which contribute to uncover novel risk genes and biological pathways implicated in the pathogenesis of asthma. Furthermore, in view of applying very strict genome-wide threshold of significance, many genetic loci with weak or modest effects were very difficult to be identified in a single GWAS, which is one of the explanations of missing heritability. Therefore, more comprehensive studies are needed to reveal the underlying effects of the small-to-modest genetic variants on severe asthma.

A growing number of studies have strongly reported that the aberrant gene expression-associated SNPs have vital parts in the etiology of complex diseases [18–21]. Recently, accumulating integrative studies have been performed for integrating the GWAS summary genetic data and expression quantitative trait loci (eQTL) data to identify the underlying regulatory effects of disease-risk genetic variants on gene expression levels. A recent article introduced a Bayesian statistical approach named as the Sherlock integrative analysis to incorporate genetic variants from GWAS summary data with eQTL data. Comparing with traditional GWAS method that commonly ignore- small or moderate effect SNPs, Sherlock integrative analysis is an effective Bayesian algorithm for employing SNPs with moderate-to-strong genetic association signals. With the use of this Bayesian tool, a growing number of studies have been performed and many novel risk genes which are very hard to be detected by traditional GWAS alone were identified in different complex diseases, such as schizophrenia [22] and major depressive disorders [23, 24].

The main goal of the current study is to identify authentic severe asthma-associated genetic loci and extend our understanding of genetic determinants influencing doctor-diagnosed severe asthma. Here, we first performed a systematically integrative genomics analysis to integrate GWAS-based SNP data with eQTL data based on the Sherlock Bayesian analysis. Furthermore, we repeated the Sherlock analysis by using two independent eQTL datasets to validate the potential biological role of these identified risk genes. In addition, through using various bioinformatics analyses based on multi-omics data, we highlighted a number of susceptible genes as promising candidates contributing risk to moderate-to-severe asthma.

## Methods

### GWAS summary datasets

#### Dataset #1 GWAS dataset on moderate-to-severe asthma

We downloaded a large-scale GWAS summary dataset on moderate-to-severe asthma reported by Shrine and coworkers [15]. We chose GWAS summary data from stage 1 for our current genomics analysis. With respect to the GWAS of stage 1, a total of 5135 patients with moderate-to-severe asthma and 25,675 controls were enrolled from the Genetics of Asthma Severity and Phenotypes (GASP) initiative and the U-BIOPRED asthma cohort [25]. All the subjects were based on European origin. The mean age of patients was 55 year old (SE ± 8). The phenotype of moderate-to-severe asthma was assessed by using clinical records according to the British Thoracic Society (BTS) 2014 guidelines. All subjects gave written and signed informed consent. The ethical approval was approved by the ethics committee for each participating clinical institution, and adhered to the standards set by International Conference on Harmonisation and Good Clinical Practice, which is registered on ClinicalTrials.gov (identifier: NCT01982162). The Affymetrix Axiom UK BiLEVE array [26] were used for genotyping and further imputation. A total of 33,771,858 SNPs were qualified for subsequent analysis.

#### Dataset #2 GWAS dataset on random phenotype of asthma

To ensure identified genes were due to genetic biology instead of artificial factors, we constructed a Null GWAS dataset as negative control. The genotype data of Null GWAS were on the basis of a reported GWAS dataset (*N* = 3960) [27], and the phenotype of Null GWAS were depended on randomly assigned the trait (asthma or control) to 3960 individuals. Considering the Null GWAS was assumed to be no true effect, the small sample size of this dataset is not a big issue. We used the PLINK (1.07v) based on the additive genetic model of the Null GWAS for a logistic regression analysis.

### Multiple eQTL datasets used for Sherlock Bayesian analysis

#### Dataset #3 Zeller et al. eQTL dataset

For this eQTL dataset reported by Zeller and coworkers [28], a total of 1490 unrelated subjects were included from a single-center cohort study of the Gutenberg Heart Study (GHS). Each chosen subject was randomly drawn from the local registry offices. Individuals signed informed consent were interviewed with a 5-h baseline examination with the collection of blood samples. All the chosen individuals’ blood samples were used for isolation of RNA and DNA. The Affymetrix Genome-wide Human SNP Array 6.0, which contains 900,392 SNPs, was utilized for genotyping. After employing a stringent quality control by considering the factors of Hardy-Weinberg equilibrium, genotype calling rate, and minor allele frequency, a number of 675,350 SNPs were eligible for subsequent analysis. Furthermore, the Illumina HT-12 V3 BeadChip was used to obtain gene expression abundance from monocyte RNA samples. After removing not well-characterized genes, a number of 12,808 genes were included for the eQTL analysis.

#### Dataset #4 Dixon et al. eQTL dataset

This dataset reported by Dixon et al. [29] was utilized as an independent eQTL dataset for validation. With regard to this dataset, Dixon and colleagues generated a global map of the genetic effects of variants on the expression levels of genes in Epstein-Barr virus-transformed lymphoblastoid cell lines of children from families enrolled through a proband with asthma. All chosen children provided written informed consent and the ethical approval was approved by the UK Multicentre Research Ethics Committee. Both the Illumina Human Hap300 Genotyping BeadChip and the Sentrix Human-1 Genotyping BeadChip were used for sample genotyping, and the Affymetrix U133 Plus 2.0 GeneChip was utilized for gaining gene expression abundance. After standard and strict inclusion criteria, a total of 400 asthmatic children with genotypes and expression abundance data were utilized to create an eQTL resource, which contains 408,273 genotyped SNPs and 20,599 genes.

#### Dataset #5 Duan et al. eQTL dataset

This dataset published by Duan et al. [30] was also used as an independent eQTL dataset for validation. The genotype data of 87 CEPH from Utah (CEU) samples were downloaded from the online HapMap database release 22 version. After filtering SNPs with Mendelian-inheritance transmission errors and minor allele frequencies < 5%, a total of 2,098,437 SNPs remain for analysis. In addition, gene expression levels of HapMap CEU lymphoblastoid cell lines were assessed by using Affymetrix GeneChip Human Exon 1.0 ST array. Finally, Duan and coworkers created an eQTL resource containing the pairwise association between 12,747 gene expression levels and 2,098,437 SNPs in the HapMap CEU population.

### Gene-level enrichment analysis

We applied the SNP-based *p* values from each GWAS summary statistics as input for gene-level enrichment analysis. The powerful bioinformatics tool of The Multi-marker Analysis of GenoMic Annotation [31] (MAGMA; more information refers to the official website: https://ctg.cncr.nl/software/magma) is used for gene-level enrichment analysis in the current investigation. Multiple regression model was employed in the MAGMA tool to combine the linkage disequilibrium (LD) information among SNPs within a specific genomic region and identify multi-variant convergent effects. SNP assigned to a gene was dependant on the location of the SNP whether mapped into the gene or within a genomic region extended +/− 20 kb downstream or upstream of the gene [32]. The LD information of SNP-SNP pairs was calculated by using the reference data of 1000 Genome European panel phase 3. The significance level of each gene was corrected by using the Bonferroni correction method. The Venn plot was generated by using the web-access tool of Draw Venn Diagram (http://bioinformatics.psb.ugent.be/webtools/Venn/).

### The method of Sherlock Bayesian analysis

The Sherlock analysis [33] is a powerful Bayesian statistical method that incorporates GWAS summary data with eQTL data to systematically discover the cis- or trans-regulatory effects of SNPs on risk genes implicated in the complex trait of interest. The procedures of this method as follows: Sherlock algorithm first uses information from eQTL dataset to find expression-associated SNPs; i.e., called eSNPs. Subsequently, the Sherlock algorithm will examine the association between eSNPs and asthma using the GWAS-based summary dataset. There were three judgmental scenarios: (1) A positive score would be assigned to an eSNP if this eSNP shows a significant association with asthma in GWAS data; (2) A negative score would be assigned to an eSNP if this eSNP has no significant association with asthma in GWAS data; (3) No score would be assigned if this SNP is only significantly associated with asthma but not an eSNP. The total score of a gene is an aggregation of the scores of eSNPs based on the integration of GWAS and eQTL data. Further, the Sherlock algorithm uses the logarithm of the Bayes factor (LBF) for a specific gene as an important indicator to predict asthma-associated risk genes. The significance of genes from Sherlock analysis was adjusted by using the Benjamini-Hochberg correction for multiple testing.

### Pathway-based analysis of risk genes

To elucidate the molecular functions of the prioritized severe asthma-associated risk genes from Sherlock analysis, we applied ClueGO, a plug-in tool of Cytoscape platform [34], to enrich significant pathways or organized biological terms. Based on the Kyoto Encyclopedia of Genes and Genomes (KEGG) which is a very popular pathway resource [35], we carried out a pathway enrichment analysis to identify significant functional links between these risk genes and biological pathways. In addition, we also conducted a Gene Ontology (GO) enrichment analysis based on three well-used categories of GO terms: molecular function, cellular component, and biological process. We also employed the feature of “*GO Term Fusion*” in the ClueGO tool to reduce the redundancies of enriched GO terms. For all enrichment analysis, the hypergeometric test was applied to assess the significance, and *P* values were adjusted by using the Bonferroni step down method.

### In silico permutation analysis

To determine whether the Sherlock-identified genes from Dataset #3 (*N* = 1129 genes) were significantly overlapped with identified genes from Sherlock analysis of Datasets #4 (*N* = 964 genes) and #5 (*N* = 771 genes) and MAGMA analysis of Dataset #1 (*N* = 1778 genes), we carried out in silico permutation analysis of 10^5^ times [36]. By randomly selecting the same size as the identified significant genes from background genes of each dataset (*N* = 9776 ~ 18,311 genes) for 100,000 times, we recorded the counts of genes overlapped with genes from Sherlock analysis of Datasets #3. Then we calculated how many times the counts of overlapped genes were larger than the number observed from real data among 100,000 trials. Empirical *P* value was permuted with the use of the probability of the observed number and *P*-value < 0.05 is treated as significance.

### Protein-protein interaction network analysis of identified risk genes

Previous studies have reported that disease-associated risk genes confer a predisposition to be of collective and functional interactions with each other [20, 37, 38]. Additionally, various protein-protein interaction (PPI) network-based analyses have been extensively utilized to identify the functional patterns of risk genes implicated in targeted phenotypes or traits [39]. Thus, we applied a PPI network analysis by using the well-used GeneMANIA tool [40], a plug-in of Cytoscape for prioritizing the promising genes for further bioinformatics analysis or lab experiments, to generate a subnetwork of these identified severe asthma-associated genes. Based on the inputted gene list, GeneMANIA software could incorporate current existing proteomics and genomics data to predict genes that functionally interacted with these identified genes.

### Asthma-associated SNPs and genes from GWAS catalog

To determine whether some of risk eSNPs or genes identified from Sherlock or MAGMA analysis are documented by GWAS Catalog, we downloaded the asthma-associated SNPs and genes information from the GWAS Catalog website (https://www.ebi.ac.uk/gwas/). From the database inception to 05 August 2019, 56 asthma-related studies reported with significant or suggestive findings. As for these 56 studies, there were a number of 846 risk SNPs and 856 mapped genes documented in the GWAS Catalog database.

### Functional annotation analysis by using HaploReg tool

To further explore the functionality of identified eSNPs from the Sherlock analysis, we used the web-based tool of HaploReg v4.1 (http://pubs.broadinstitute.org/mammals/haploreg/haploreg.php) [41] to perform afunctional annotation analysis. The vast majority of disease-associated common variants identified from GWAS studies might regulate gene expression instead of directly affect the function of proteins. The HaploReg was developed to aid the function dissection of GWAS findings and predict putative causal variants that were LD with tag SNPs. Based on systematically comparative, epigenomic and regulatory annotation, the HaploReg tool also could be used to predict targeted genes associated with traits of interest [41].

### Gene expression patterns between severe asthma and control

The assumption of identifying disease-relevant risk genes from the Sherlock Bayesian algorithm is that the aberrant expression of risk genes involve in the development of disease of interest. Thus, we utilized two RNA expression datasets downloaded from the NCBI GEO database (Accession Nos. GSE130499 and GSE123750) to validate the functionality of identified severe asthma-associated genes. The first dataset of GSE130499 based on bronchial epithelial cells (*N* = 154 participants) [42] was designed to identify the differential gene expression (DEG) profiles between severe asthma (SA, *N* = 44), mild-moderate asthma (notSA, *N* = 72), and normal control (Control, *N* = 38). The mean age of individuals with severe asthma was 47 year old (SE ± 1.7). The second dataset of GSE123750 includes blood transcriptomics profiles of school-aged children diagnosed with severe (*N* = 75) and mild-moderate asthma (*N* = 37) from the U-BIOPRED asthma project. We could use this dataset to examine whether these identified risk genes are prone to be vulnerable to severe asthma. Corrplot R package was used to compare the different co-expression patterns of identified genes among three groups. One-way ANOVA analysis with Tukey post hoc analysis was performed for calculating the significance of gene expression among SA, notSA, and control groups. Significance between severe asthma and mild-moderate asthma group was calculated by using Student’s T-test. *P* < 0.05 was considered to be significant.

### Gene expression profiles among various glucocorticoids for severe asthma

The gene expression data of inhaled glucocorticoids (GCs) for severe asthma was downloaded from the NCBI GEO database (Accession No. GSE119789) [43]. The Agilent SurePrint G3 Mouse Gene Expression Microarray v.2 was employed to profile gene expression pattern of GCs, including Dexamethasone (DEX), Fluticasone Furoate (FF), VSG158, and VSG159, and control treatment of EtOH DMSO (Vehicle) in the mouse macrophage RAW264.7 cells. RAW264.7 cells were treated with designated steroids at 100 nM 1 h before overnight lipopolysaccharide stimulation of inflammation. There are 3 biological replicates in each group. Thus, we explored whether these effective GCs have significant links with severe-associated risk genes identified from our Sherlock analysis based on large GWAS and eQTL data. Heatmap was generated by using pheatmap R package. Corrplot R package was employed to compare the distinct expression profiles of risk genes with the different treatment of GCs. One-way ANOVA analysis with Tukey post hoc test was carried out for calculating the significance.

## Results

### Prioritization of asthma-associated risk genes using the Sherlock analysis

**Fig. 1:**
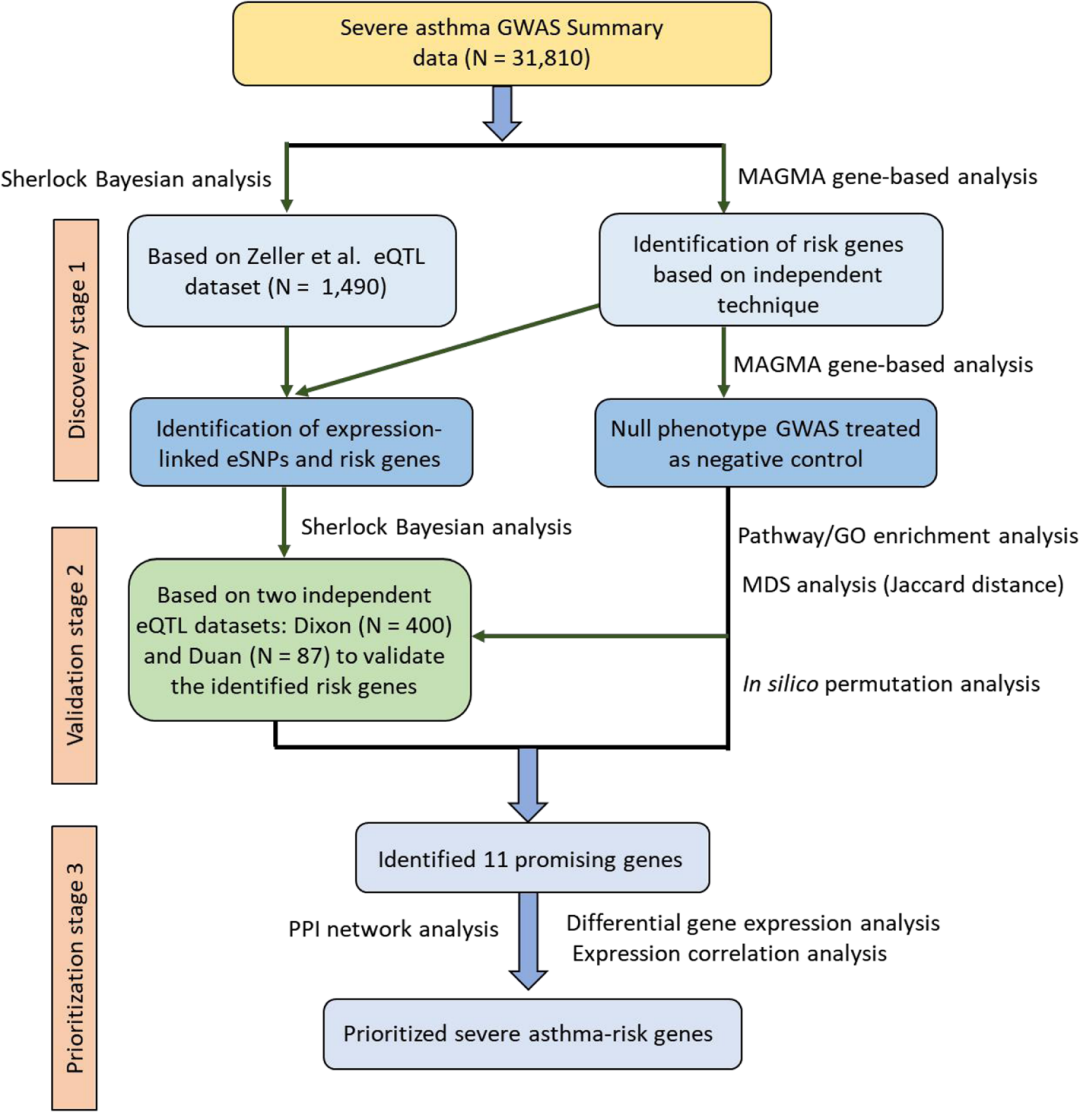
The overall workflow of current investigation

In the first place, we employed Sherlock Bayesian inference method to reveal whether aberrant gene expression confers risk to moderate-to-severe asthma by incorporating GWAS summary data (Dataset #1, *N* = 31,810) and eQTL data (Dataset #3, *N* = 1490). All procedures of the present investigation are shown in Fig. 1. In this discovery analysis, we identified a number of 1129 significant genes to be associated with moderate-to-severe asthma risk through altering in its expression (Simulated *P*-value < 0.05, Supplemental Table S1). For example, the top-ranked risk genes with significant eSNPs conferring risk to asthma: *HLA-DRB3/HLA-DRB5/HLA-DRB4/HLA-DRB1* (Simulated *P* = 7.93 × 10^− 7^), *RBM43* (Simulated *P* = 1.59 × 10^− 6^), *HLA-DOB* (Sherlock-based P = 1.59 × 10^− 6^), *IKZF3* (Simulated P = 1.59 × 10^− 6^), *IL18R1* (Simulated P = 1.59 × 10^− 6^), *GNGT2* (Simulated *P* = 1.11 × 10^− 5^), *MAP2K5* (Simulated *P* = 2.54 × 10^− 5^), and *HLA-DQA1* (Simulated *P* = 2.85 × 10^− 5^). Although numerous genes have been reported to be associated with asthma in the GWAS Catalog, a greater number of novel risk genes were identified in our current Bayesian analysis (Supplemental Table S1).

### MAGMA gene-based analysis of GWAS on moderate-to-severe asthma

Furthermore, we used an independent technique of gene-based analysis for identifying more reliable genes contributing risk to moderate or severe asthma. The MAGMA gene-based analysis revealed significant 1778 genes (MAGMA-based *P* < 0.05) to be associated with moderate-to-severe asthma. The association signals of gene-based analysis were yielded by *HLA-DQA1* (MAGMA-based *P* = 2.90 × 10^− 23^), *HLA-DQB1* (MAGMA-based *P* = 3.98 × 10^− 21^), *IL18R1* (MAGMA-based *P* = 4.99 × 10^− 17^), *CLEC16A* (MAGMA-based *P* = 5.27 × 10^− 17^), *DEXI* (MAGMA-based *P* = 9.70 × 10^− 15^), *TSLP* (MAGMA-based *P* = 2.99 × 10^− 14^), *SMAD3* (MAGMA-based *P* = 1.21 × 10^− 13^), and *IL1RL1* (MAGMA-based *P* = 1.52 × 10^− 13^). Consistently, there were a number of 228 genes overlapped with the results of Sherlock analysis in the discovery stage (Supplemental Table S2).

### Pathway-based analysis of 228 identified risk genes

**Fig. 2:**
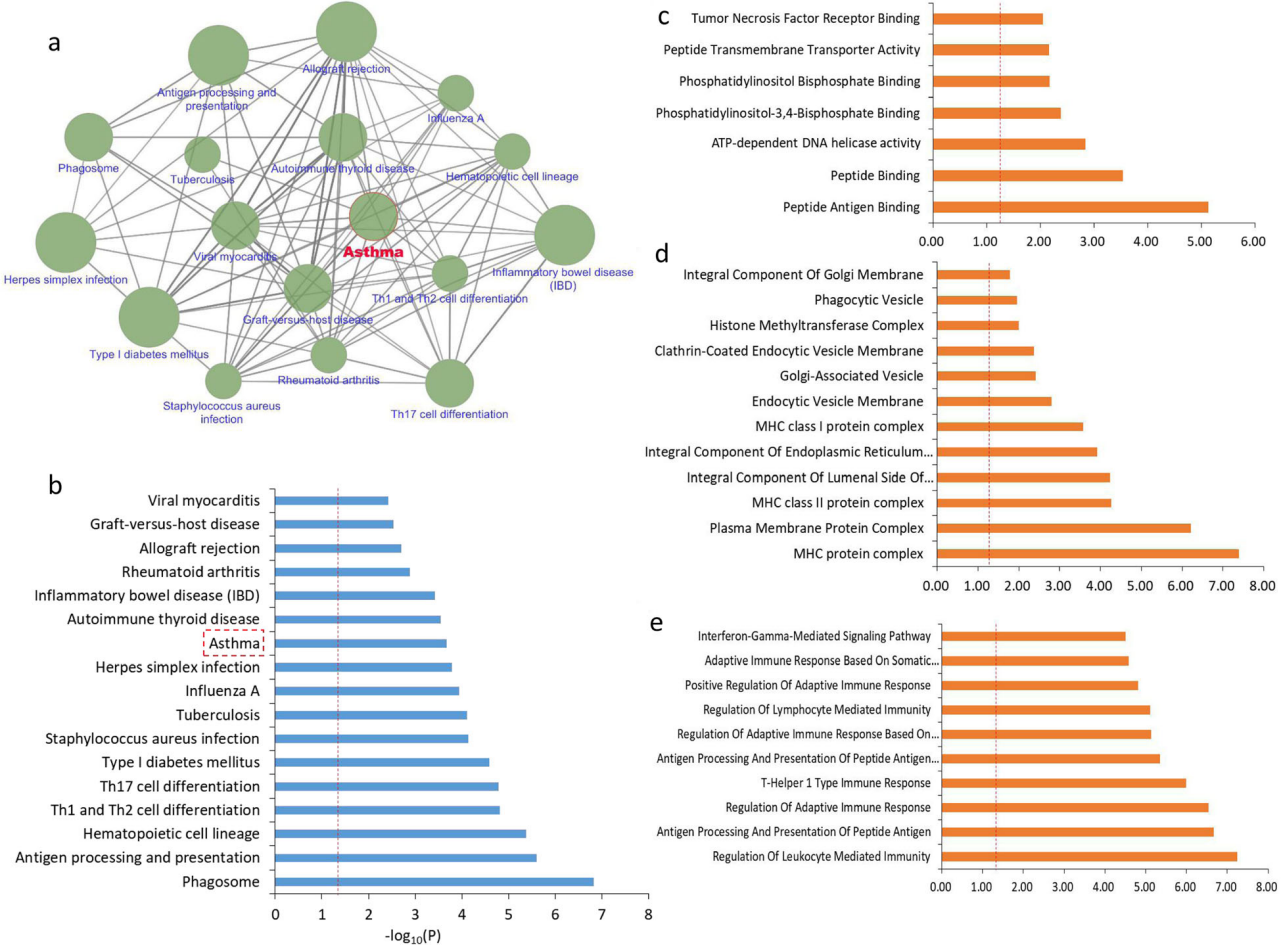
Pathway-based enrichment of 228 severe asthma-relevant genes. **a** The interaction network of enriched 17 biological pathways. The red color marked the pathway of asthma. **b** KEGG pathway enrichment analysis for 228 identified genes with 17 biological pathways enriched. **c** GO-terms of molecular function enrichment analysis for 228 identified genes. **d** GO-terms of cellular component enrichment analysis for 228 identified genes. **e** GO-terms of biological process enrichment analysis for 228 identified genes (Top-ranked). The red vertical line represents that the threshold of the *P*-value after the Bonferroni step down correction

Through using these identified 228 risk genes, we carried out a pathway-based enrichment analysis based on the KEGG pathway resource. There were 17 biological pathways prominently overrepresented by these identified genes (Fig. 2a-b and Supplemental Table S3). These significantly enriched pathways have functionally interacted with each other (Fig. 2a). The top-ranked significant pathways were antigen processing and presentation (Corrected *P* = 4.30 × 10^− 6^), type I diabetes mellitus (Corrected *P* = 7.09 × 10^− 5^), inflammatory bowel disease (Corrected *P* = 1.14 × 10^− 4^). Of note, the pathway of asthma (Corrected *P* = 1.72 × 10^− 3^) was significantly enriched by these 228 risk genes (Fig. 2b and Supplemental Table S3). In addition, we further performed GO-term enrichment analysis according to 3 categories of molecular function, cellular component, and biological process, respectively. With regard to the terms of molecular function (Fig. 2c and Supplemental Table S4), these identified risk genes were significantly overrepresented in 7 terms, including peptide antigen binding (Corrected *P* = 5.17 × 10^− 5^), peptide binding (Corrected P = 1.72 × 10^− 3^), and ATP-dependent DNA helicase activity (Corrected *P* = 7.15 × 10^− 3^). For the terms of cellular component (Fig. 2d and Supplemental Table S5), 12 GO-terms were significantly overrepresented; e.g., MHC protein complex (Corrected *P* = 4.91 × 10^− 7^), plasma membrane protein complex (Corrected *P* = 6.79 × 10^− 6^), and class II protein complex (Corrected *P* = 5.43 × 10^− 4^). With respect to the terms of biological process (Fig. 2e, Supplemental Fig. S1, and Supplemental Table S6), we detected a number of 32 significantly enriched GO-terms; e.g., regulation of leukocyte mediated immunity (Corrected *P* = 4.22 × 10^− 6^), antigen processing and presentation of peptide antigen (Corrected *P* = 1.58 × 10^− 5^), and regulation of adaptive immune response (Corrected *P* = 2.12 × 10^− 5^).

### Two independent eQTL datasets for validation

**Fig. 3:**
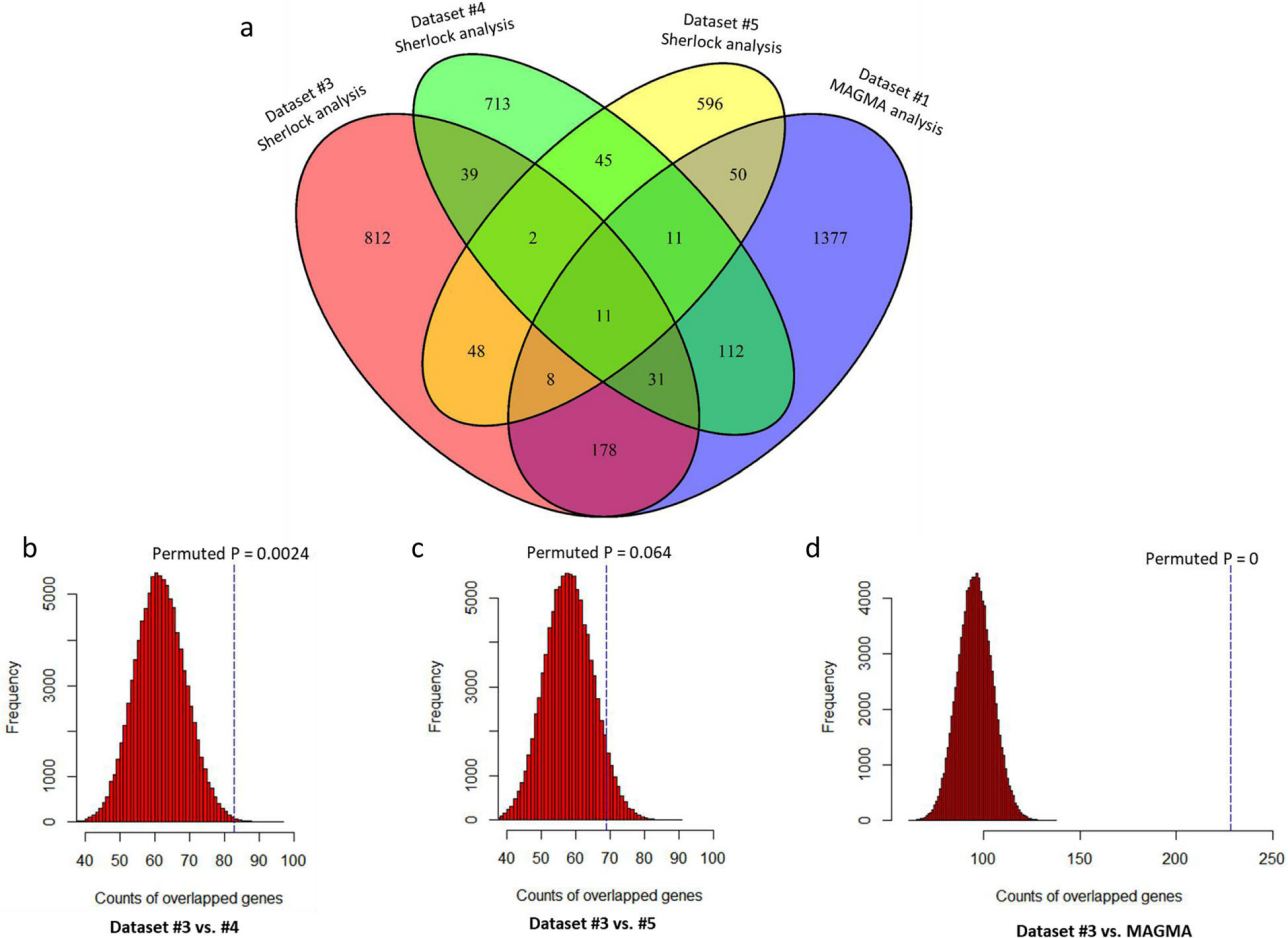
Consistent findings of severe asthma-relevant risk genes from independent datasets. **a** Venn plot of severe asthma-relevant genes based on four datasets: Dataset #1 (MAGMA analysis), Dataset #3 (Sherlock analysis), Dataset #4 (Sherlock analysis), and Dataset #5 (Sherlock analysis). There were 11 promising genes validated among all four datasets. **b** In silico permutation analysis (100,000 times) of the counts of risk genes from Dataset #3 overlapped with that from Dataset #4. **c** In silico permutation analysis (100,000 times) of the counts of risk genes from Dataset #3 overlapped with that from Dataset #5. **d** In silico permutation analysis (100,000 times) of the counts of risk genes from Dataset #3 overlapped with genes from MAGMA analysis of Dataset #1

We re-performed the Sherlock Bayesian analysis with the same parameter settings by using two independent expression QTL datasets (Datasets #4 and #5). For the eQTL data from Dataset #4, Sherlock analysis detected a number of 964 significant or suggestive risk genes (Simulated *P* value < 0.05, Fig. 3a). The top signals of this dataset were *HLA-DQA2* (Simulated *P* = 7.73 × 10^− 7^), *HLA-DQA1* (Simulated P = 7.73 × 10^− 7^), and *P4HA2* (Simulated P = 7.73 × 10^− 7^). For the eQTL data from Dataset #5, Sherlock analysis detected a group of 771 significant or suggestive risk genes (Simulated P value < 0.05, Fig. 3a). The top signals of this dataset were *ORMDL3* (Simulated *P* = 1.02 × 10^− 6^), *HLA-DRB5/HLA-DRB1/HLA-DQB1/HLA-DQB2* (Simulated *P* = 4.09 × 10^− 6^), and *ZNF749* (Simulated *P* = 2.05 × 10^− 5^).

**Fig. 4:**
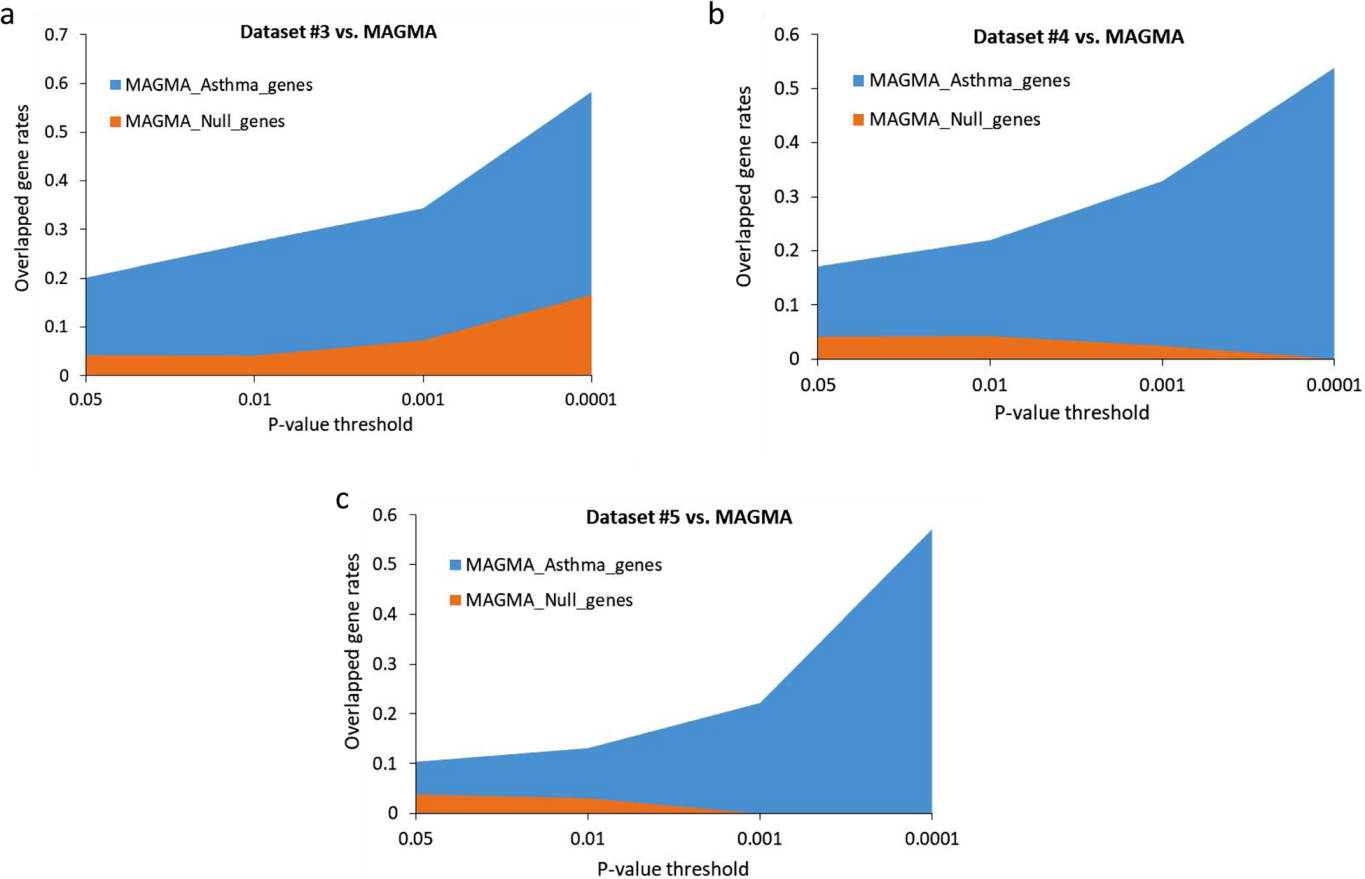
Sherlock-identified risk genes overlapped with MAGMA-identified genes. **a** Sherlock-identified genes from Dataset #3 were higher overlapped with genes from asthma-based GWAS (Dataset #1) than that from Null-based GAWS (Dataset #2) at four different P-value thresholds of 0.05, 0.01, 0.001, and 0.0001. **b** Sherlock-identified genes from Dataset #4 were higher overlapped with genes from asthma-based GWAS (Dataset #1) than that from Null-based GAWS (Dataset #2) at four different P-value thresholds of 0.05, 0.01, 0.001, and 0.0001. **c** Sherlock-identified genes from Dataset #5 were higher overlapped with genes from asthma-based GWAS (Dataset #1) than that from Null-based GAWS (Dataset #2) at four different P-value thresholds of 0.05, 0.01, 0.001, and 0.0001

To further replicate the reliability of above identified risk genes, we compared these Sherlock-identified genes from Datasets #4 and #5 with two groups of identified genes from Datasets #3 and #1, indicating there exist a considerable number of genes overlapped among these 4 groups (Fig. 3a). We found that Sherlock-identified genes from Dataset #3 in the discovery stage showed a significantly or marginally higher overlap with genes from Datasets #4 (Permuted *P* = 0.0024; Fig. 3b), #5 (Permuted *P* = 0.064; Fig. 3c), and #1 (Permuted P = 0, i.e., very significant; Fig. 3d) than that of random selection from background genes. In addition, we observed that Sherlock-identified genes from Datasets #3, #4, and #5 have relatively higher overlapped gene rates with MAGMA-identified genes from asthma GWAS data than that from Null GWAS data (Dataset #2) (Fig. 4a,b,c).

From technique and biological validation from these independent datasets, there were a number of 11 risk genes common across all analyses (Fig. 3a and Table 1). Interestingly, 4 genes of *MPI*, *DECR2*, *LNPEP*, and *TTC19* are newly reported to be involved in severe asthma risk. Based on Null GWAS data, we found that all these 11 identified genes showed non-significant associations with the random phenotype of asthma (Table 1).

**Table 1:** Integrative genomics analysis highlighted 11 highlighted genes contributed to moderate-to-severe asthma risk

Gene name	Simulated ***P***-value (Sherlock analysis of Dataset #3)	Simulated ***P-***value (Sherlock analysis of Dataset #4)	Simulated ***P***-value (Sherlock analysis of Dataset #5)	MAGMA-based ***P***-value (Dataset #1)	MAGMA-based ***P*** value (Dataset #2, negative control)	GWAS Catalog documented genes
*HLA-DRB5*	7.93 × 10^−7^	1.60 × 10^−3^	4.09 × 10^−6^	7.57 × 10^−10^	0.11	Documented gene
*HLA-DRB1*	7.93 × 10^−7^	4.64 × 10^−6^	4.09 × 10^− 6^	1.41 × 10^−11^	0.59	Documented gene
*GNGT2*	1.11 × 10^−5^	2.30 × 10^−4^	1.83 × 10^−2^	1.42 × 10^− 2^	0.48	Documented gene
*HLA-DQA1*	2.85 × 10^−5^	7.73 × 10^−7^	1.49 × 10^−3^	2.90 × 10^−23^	0.58	Documented gene
*SLC22A5*	1.85 × 10^−4^	3.87 × 10^−5^	2.66 × 10^− 5^	1.23 × 10^−6^	0.46	Documented gene
*STAT6*	2.19 × 10^−4^	2.75 × 10^−2^	3.31 × 10^− 2^	1.94 × 10^−11^	0.97	Documented gene
*MPI*	1.90 × 10^−3^	2.02 × 10^−2^	2.39 × 10^−2^	1.12 × 10^−5^	0.84	Not documented gene
*TLR6*	3.63 × 10^−3^	3.54 × 10^− 3^	6.69 × 10^− 3^	4.10 × 10^−2^	0.93	Documented gene
*DECR2*	1.62 × 10^−2^	4.32 × 10^− 2^	1.58 × 10^−4^	2.22 × 10^− 2^	0.78	Not documented gene
*LNPEP*	2.28 × 10^−2^	5.55 × 10^−4^	4.33 × 10^−2^	3.98 × 10^− 2^	0.46	Not documented gene
*TTC19*	2.52 × 10^−2^	1.81 × 10^−2^	2.20 × 10^− 2^	3.23 × 10^− 2^	0.30	Not documented gene

### PPI network-based analysis of 11 highlighted risk genes

**Fig. 5:**
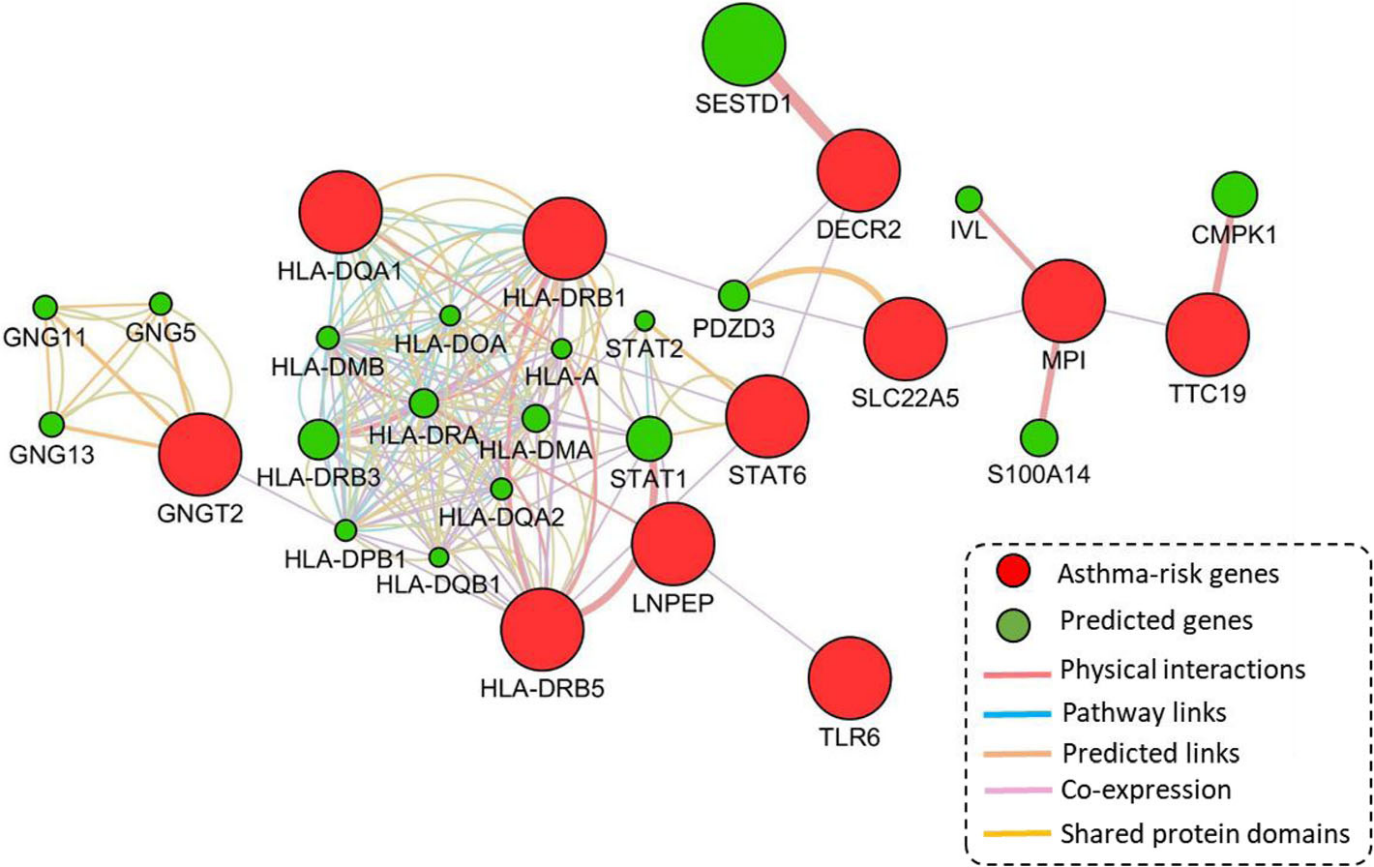
Protein-protein interaction networks among 11 highlighted asthma-associated genes. The 11 asthma-risk genes are shown in red color and the predicted genes are shown in green color. The predicted attributes included physical interactions, pathway links, co-expression, predicted links, and shared protein domains

To reveal the functional interactions of 11 highlighted risk genes associated with moderate-to-severe asthma, we performed a PPI network enrichment analysis with the use of the GeneMANIA bioinformatics tool [40]. Figure 5 demonstrated that these identified 11 asthma-associated genes were highly interacted together with multiple layers of evidence, encompassing physical interactions, pathway links, co-expression correlations, predicted links, and shared protein domains. These 11 highlighted risk genes accompanied with the other 19 predicted genes constructed a biological sub-network, which potentially implicated in the pathogenesis of severe asthma. For example, the hub genes of *MPI*, *TTC19*, and *SLC22A5* show evidence of co-expressions (Fig. 5). The important gene of *GNGT2* showed high interactions with other genes in this sub-network. Furthermore, the well-reported asthma-associated genes of *HLA-DQA1*, *HLA-DRB1*, and *HLA-DRB5* have the most number of interacted edges with identified and predicted genes (Fig. 5).

### Differential gene expression patterns among severe asthma different datasets

**Fig. 6:**
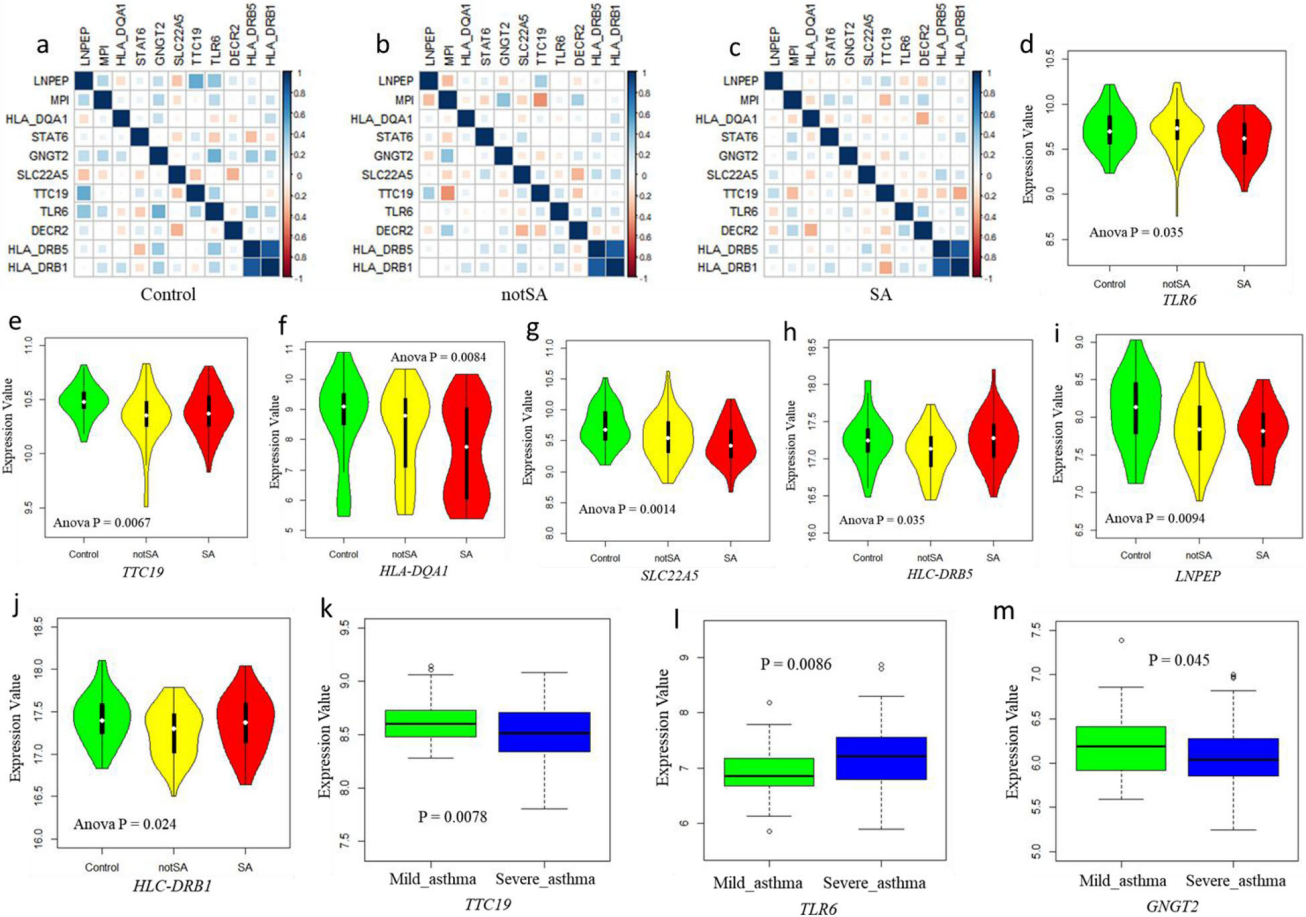
Differential expression patterns of 11 highlighted genes between severe asthma and control. **a** Co-expression patterns of 11 highlighted genes in control group. **b** Co-expression patterns of 11 highlighted genes in notSA group. **c** Co-expression patterns of 11 highlighted genes in SA group. **d - j** Violin plots show the differential expression profiles of highlighted genes among control, notSA, and SA group; **d** for *TLR6*, **e** for *TTC19*, **f** for *HLA-DQA1*, **g** for *SLC22A5*, **h** for *HLC-DRB5*, **i** for *LNPEP*, **j** for *HLC-DRB1*. **k** - **m** Boxplots show the differential expression patterns of *TTC19* (**k**), *TLR6* (**l**), and *GNGT2* (**m**) between severe asthma and mild-moderate asthma

**Fig. 7:**
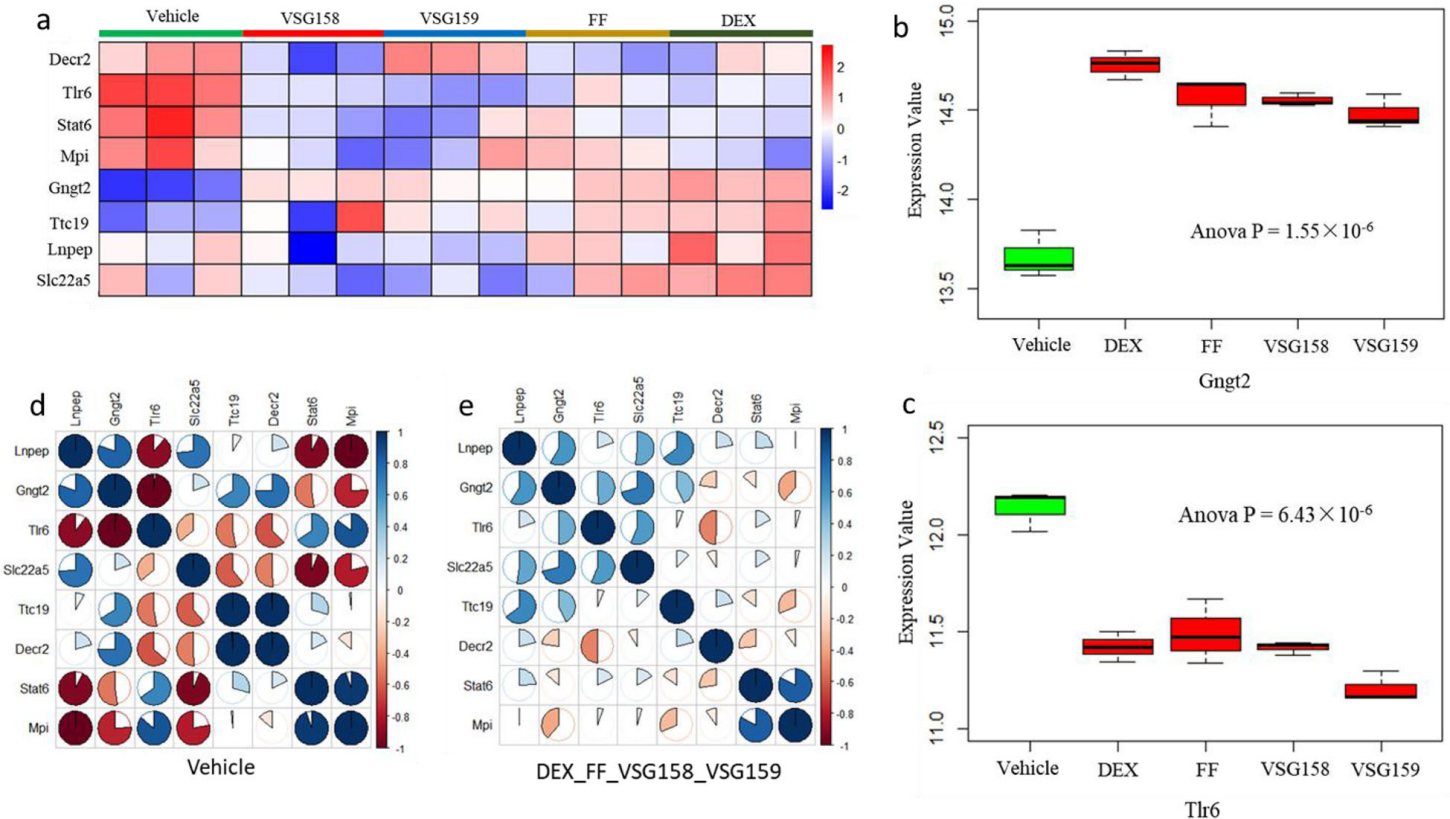
Altered gene expression profiles treated with various glucocorticoids for severe asthma. **a** The heatmap shows the differential expression profiles between vehicle and various glucocorticoids including VSG158, VSG159, FF, and DEX. **b** Boxplot shows the significant difference of Gngt2 expression between vehicle and various glucocorticoids including VSG158, VSG159, FF, and DEX. **c** Boxplot shows the significant difference of Tlr6 expression between vehicle and various glucocorticoids including VSG158, VSG159, FF, and DEX. **d** Co-expression patterns of identified genes in vehicle group. **e** Co-expression patterns of highlighted genes in glucocorticoid group (including VSG158, VSG159, FF, and DEX)

To further validate the functionality of 11 highlighted risk genes, we tested the different co-expression profiles of these genes among SA, notSA, and control group in the GSE130499 dataset. We detected that the co-expression patterns of these 11 important genes among SA and notSA group showed obvious differences by comparison with control group (Fig. 6a-c). There were 7 genes showing significantly abnormal expressions among 3 groups (Fig. 6d-j and Supplemental Fig. S2); for example *TLR6* (ANOVA *P* = 0.035) and *TTC19* (ANOVA *P* = 0.0067). Meanwhile, in an independent dataset of GSE123750, we found that *TTC19* (*P* = 0.0078; Fig. 6k), *TLR6* (*P* = 0.0086; Fig. 6l), and *GNG12* (*P* = 0.045; Fig. 6m) were significantly expressed in severe asthma by comparison with mild-moderate asthma. In addition, we observed that there existed distinct expression patterns between vehicle and various GCs including VSG158, VSG159, FF, and DEX in the dataset of GSE119789 (Fig. 7a and Supplemental Fig. S3); for example, Gngt2 (ANOVA *P* = 1.55 × 10^− 6^; Fig. 7b) and Tlr6 (ANOVA *P* = 6.43 × 10^− 6^; Fig. 7c). Consistently, we also detected that the co-expression patterns of these 11 important genes were distinctly altered by using various GCs comparing with vehicle (Fig. 7d-e and Supplemental Fig. S4).

### Identification of risk eSNPs among these 11 identified asthma-risk genes

For each of these 11 identified genes, we found multiple important eSNPs showing significant association with the expression of this gene and moderate-to-severe asthma risk simultaneously (Supplemental Table S7). To name a few, for the gene of *TLR6*, there were 3 cis-regulatory eSNPs of rs11466640 (P_eQTL_ = 1.64 × 10^− 7^ and P_GWAS_ = 2.0 × 10^− 3^), rs5743592 (P_eQTL_ = 3.0 × 10^− 6^ and P_GWAS_ = 2.49 × 10^− 3^), and rs5743595 (P_eQTL_ = 4.5 × 10^− 7^ and P_GWAS_ = 2.61 × 10^− 3^) and one trans-regulatory eSNP of rs11130280 (P_eQTL_ = 5.0 × 10^− 6^ and P_GWAS_ = 1.83 × 10^− 2^). As for *GNGT2* gene, 3 SNPs of rs17637472 (P_eQTL_ = 2.98 × 10^− 8^ and P_GWAS_ = 3.40 × 10^− 8^), rs11265180 (P_eQTL_ = 6.0 × 10^− 6^ and P_GWAS_ = 1.99 × 10^− 3^), and rs1867087 (P_eQTL_ = 1.0 × 10^− 4^ and P_GWAS_ = 1.84 × 10^− 5^) were severe asthma-associated eSNPs. To replicate this findings, we used the web-based tool of HaploReg [41] and found 2 eSNPs of rs17637472 and rs1867087 were significantly associated with the expression of *GNGT2* in whole blood cells (*P* = 2.13 × 10^− 52^ and *P* = 3.79 × 10^− 22^, Supplemental Table S8). In addition, *GNGT2* gene contains some suggestive asthma-associated SNPs (Supplemental Fig. S5); e.g. rs648980 (P_GWAS_ = 1.93 × 10^− 4^), rs617182 (P_GWAS_ = 1.95 × 10^− 4^), rs55978930 (P_GWAS_ = 2.79 × 10^− 4^), rs113201977 (P_GWAS_ = 4.14 × 10^− 4^). The polymorphisms of rs648980 (P_eQTL_ = 1.40 × 10^− 32^) and rs617182 (P_eQTL_ = 6.25 × 10^− 34^) also showed a significant association with *GNGT2* expression level in whole blood samples [44], and rs55978930 (P_eQTL_ = 2.52 × 10^− 6^) is significantly associated with *GNGT2* expression level in thyroid tissue [45].

## Discussion

Asthma is a heterogeneous and chronic airways disease, and it is estimated to influence 235–334 million individuals worldwide [1, 2, 46]. Approximately 15% of asthma patients are treated for severe asthma, which is in relation to poor control and response to treatment [3, 4, 47]. Severe asthma is influenced by both environmental and genetic components [5–7]. There is a considerable interest in improvement of our understanding of the biological mechanism of severe asthma by using genetics and genomics approaches. Current investigation based on comprehensive genomics integrative analysis is designed to prioritize important risk genes associated with moderate-to-severe asthma.

In general, GWAS is a wide-used and effective method for identifying disease-associated common SNPs. To date, through using GWAS, thousands of SNPs have been reported to be associated with complex diseases/traits of interests [48, 49]. Consistently, many studies have demonstrated that numerous SNPs were identified to be associated with asthma [2, 8–14]. But, only several GWASs reported to identify risk variants for moderate-to-severe/severe asthma [15, 50, 51]. Because of examining millions of SNPs at one experiment of GWAS which employs very stringent correction methods for multiple testing, the power of GWAS has been remarkably constrained. Furthermore, considering numerous SNPs were highly linked with each other, these previously reported asthma-associated SNPs appeared to be accompanied with several highly linkage disequilibrium (LD) SNPs with similar *P* values. Thus, to confirm the exact causal SNPs became a very tough and necessary job. In light of the vast majority of identified SNPs being located in the non-coding genomic regions, it is plausible to infer that identified SNPs contribute to asthma risk through mediating the expression level of the causative genes. Thus, the Sherlock Bayesian analysis is a powerful method to identify asthma-associated novel risk genes and eSNPs which cannot be identified by any single GWAS study.

In the present study, we utilized the Sherlock Bayesian analysis to integrate a large-scale GWAS summary dataset (*N* = 30,810) with an eQTL dataset (*N* = 1490) as discovery dataset for identifying susceptible genes and SNPs associated with moderate-to-severe asthma. We identified 1129 significant genes with eSNPs to be associated with moderate-to-severe asthma. Some of the top-ranked significant genes, e.g., *HLA-DRB3*, *HLA-DRB5*, and *HLA-DRB4*, have been widely documented to be involved in mild asthma pathogenesis in previous studies [2, 11–16, 52], which is in line with previous finding that there exist shared genetic components between mild and moderate-to-severe asthma [47]. Furthermore, we employed an independent technique of MAGMA gene-based analysis, which has been extensively used for identifying susceptible genes associated with complex diseases [31, 53–56], to validate Sherlock-identified genes in the discovery stage. Interestingly, 228 genes were significantly replicated. These identified genes were significantly enriched in 17 biological pathways, including the pathway of antigen processing and presentation, type I diabetes mellitus, inflammatory bowel disease, and asthma. These results provide a mechanistic clue for further experimental validation. To improve the reliability of our findings, we used two independent eQTL datasets for biological validation, and found that Sherlock-identified genes in the discovery stage were higher overlapped with Sherlock- and MAGMA-identified genes in replication stages than that of random selections. Together, based on biological and technical validation, the findings of our current integrative genomics analysis are reliable.

Subsequently, through performing joint bioinformatics analyses, including MAGMA gene analysis, pathway enrichment analysis, in silico permutation analysis, PPI network analysis, co-expression analysis, and DEG analysis, we highlighted 11 important genes, such as *GNGT2*, *TLR6*, and *TTC19*, may represent authentic risk genes for moderate-to-severe asthma. By using the PPI network enrichment analysis, we observed that these 11 highlighted genes together with the other 19 predicted genes constructed a biological sub-network, suggesting that these genes have collective functions in severe asthma-related risk rather than false-positives. In view of the assumption of genes identified from Sherlock Bayesian analysis is that the expression level of risk genes with eSNPs are abnormally changed in individuals at disease status [33], the expression of identified genes should be dysregulated in severe asthmatic patients if the Sherlock-identified genes truly confer risk to asthma. Thus, we performed DEG analyses using 2 RNA expression datasets on severe asthma and 1 RNA expression dataset on the inhaled glucocorticoids treatment for severe asthma. We found that there exist different co-expression patterns of these 11 highlighted genes among severe asthma, not severe asthma and control group, as well as between vehicle and various GCs, indicating that the altered expression of these 11 genes may implicate in the etiology of severe asthma. There were 8 of 11 genes (8/11 = 72.7%) showing significantly different expression profiles between severe asthma and controls; for example, *GNGT2*, *TLR6*, *TTC19*, *LNPEP*, *SLC22A5* and *HLA-DQA1*.

For the important gene of *GNGT2*, there were 3 eSNPs of rs17637472, rs11265180, and rs1867087 associated with moderate-to-severe asthma risk. 2 SNPs of rs17637472 and rs1867087 have identified to be significantly associated with the expression level of *GNGT2* gene [44]. Consistently, the polymorphism of rs17637472 was reported to be the strongest cis-eQTL for *GNGT2* gene [57, 58]. Demenais and colleagues [16] demonstrated that the polymorphism of rs17637472 is a susceptibility locus for asthma based on a large-scale GWAS (*N* = 142,486) from ethnically-diverse populations. Ferreira and coworkers [12] documented that the *GNGT2* gene was predicted targets of a sentinel risk SNP of rs12952581 for asthma. In addition, we also observed several suggestive asthma-associated SNPs (e.g., rs648980, rs617182, and rs55978930) were mapped into the gene of *GNGT2*, indicating the association signals of this gene was not alone and reflecting this gene predisposes to be a genuine risk gene rather than false-positive one. The protein encoded by *GNGT2* gene is thought to play an important role in cone phototransduction. It belongs to the G protein gamma family and is localized specifically in cones [59]. GNGT2 protein has an interaction with beta-arrestin 1 for promoting G-protein-dependent Akt signaling to activate NF-kappaB [60]. A previous genome-wide methylation analysis [61] based on the Illumina Human Methylation 450 K array of whole blood samples (*N* = 724) has demonstrated that the CpG site of cg00980784 in *GNGT2* was significantly associated with current smoking compared with never smoking after correction for multiple testing. Smoking is a well-established risk factor for the development of asthma [62].

The protein encoded by *TLR6* gene is a member of the Toll-like receptor (TLR) family, which regulate immune system pathogen recognition and activate innate immunity. Clinical experiments demonstrated that Ser249Pro polymorphism in *TLR6* has a protective effect on asthma [63, 64]. Furthermore, genetic variants in TLR6 exerting their protection on asthma were linked with greater mononuclear cell generation of Th1-type cytokines [65]. Based on a mouse model, Moreira et al. [66] showed that the protective role of TLR6 for asthma is regulated by IL-23 and IL-17A. In our current analysis, there were 4 eSNPs of rs5743595 (P_eQTL_ = 4.5 × 10^− 7^ and P_GWAS_ = 2.61 × 10^− 3^), rs11130280 (P_eQTL_ = 5.0 × 10^− 6^ and P_GWAS_ = 1.83 × 10^− 2^), rs5743592 (P_eQTL_ = 3.0 × 10^− 6^ and P_GWAS_ = 2.49 × 10^− 3^), and rs11466640 (P_eQTL_ = 1.64 × 10^− 7^ and P_GWAS_ = 2.0 × 10^− 3^) in *TLR6* associated with moderate-to-severe asthma risk. In addition, numerous SNPs in the gene of *TLR6* were reported to be associated with allergic disease [67–69].

Among these 11 identified genes, there were 4 newly discovered genes of *MPI*, *DECR2*, *LNPEP*, and *TTC19*. For *TTC19* gene, it encodes a protein with a tetratricopeptide repeat (TPR) domain, which is embedded in the inner mitochondrial membrane and is involved in the formation of the mitochondrial respiratory chain III [70]. There were seven eSNPs with cis- or trans-regulatory roles in *TTC19* to be associated with severe asthma. For example, the cis-regulatory eSNP of rs3785631 is associated with severe asthma (P_GWAS_ = 6.37 × 10^− 3^) and gene expression of *TTC19* (P_eQTL_ = 1.79 × 10^− 17^). The genetic variation in *LNPEP*, which is a zinc-dependent aminopeptidase that cleaves vasopressin, was reported to be associated with 28-day mortality in septic shock [71]. Here we found nine eSNPs in *LNPEP* to be associated with severe asthma. With regard to *MPI*, there were four eSNPs to be associated with severe asthma. Together, our findings showed that these 11 genes convey risk of moderate-to-severe/severe asthma and worth us to do further experimental validation.

Some limitations are warranted to comment. First, although we collected multi-layers of omics data, there existed other omics data missed in our current investigation. For example, gene expression datasets used in current analysis are derived from lymphoblastoid cell lines and monocytes. Further studies are needed to assess tissues that could be more relevant to asthma pathogenesis, such as nasal or lung tissues. Second, because of the heterogeneity of used datasets, we used different correction methods for multiple testing for each individual omics data, e.g., simulated *P* < 0.05 in Sherlock analysis, MAGMA-based P < 0.05 in MAGMA analysis, FDR < 0.05 for pathway enrichment analysis, and ANOVA P < 0.05 for DEG analysis. Moreover, our current integrative genomics analysis found many SNPs showed significant association among European population. We did not explore their effects on asthma in other ancestries. For example, the SNP of rs17637472 showed significant association not only in Europeans but also in a multi-ethnic meta-analysis, suggesting that the eQTL effect may be also extensible to individuals of other ancestries. However, this inference should be evaluated by using genotype and gene expression data from populations of different ethnicities.

## Conclusions

In summary, current study provides several lines of evidence to support that 11 highlighted genes including *GNGT2*, *TLR6*, and *TTC19* could be treated as genuine moderate-to-severe/severe asthma-associated genes. Through incorporating GWAS summary-based genetic information with eQTL data, we offered a reasonable explanation of the biological functions of genetic variants on severe asthma risk. The results of current investigation give several eSNPs and risk genes for subsequent functional experimentations to explore the biological mechanism of developing severe asthma.

## Supplementary information

**Additional file 1.**

**Additional file 2.**

## Data Availability

GWAS summary dataset on moderate-to-severe asthma was downloaded from the GWAS catalog resource (ftp://ftp.ebi.ac.uk/pub/databases/gwas/summary_statistics/). The accession number of this GWAS summary dataset is ShrineN_30552067_GCST006911. The eQTL dataset for discovery was used from the official website (http://sherlock.ucsf.edu/submit.html, Zeller_10). The eQTL datasets for independent validation were available in the official website (http://sherlock.ucsf.edu/submit.html, Dixon_7 and Duan_08). Three RNA expression datasets (Accession Nos. GSE130499, GSE123750, and GSE119789) were downloaded from the NCBI GEO database (https://www.ncbi.nlm.nih.gov/geo/).

## Abbreviations

GWAS: genome-wide association study
eQTL: expression quantitative trait loci
eSNP: expression-linked single nucleotide polymorphism
GO: gene ontology
KEGG: Kyoto Encyclopedia of Genes and Genomes
GEO: Gene expression omnibus
PPI: protein-protein interaction
LD: linkage disequilibrium
DGE: differential gene expression
GASP: the Genetics of Asthma Severity and Phenotyoes
BTS: The British Thoracic Society
GHS: the Gutenberg Heart Study
LBF: the Bayes factor
GCs: inhaled glucocorticoids
DEX: Dexamethasone
FF: Fluticasone Furoate
