# Supplementary material for: Integrated genomics analysis highlights important SNPs and genes implicated in moderate-to-severe asthma based on GWAS and eQTL datasets
Source: BMC Pulm Med. 2020 Oct 16;20:270. doi: 10.1186/s12890-020-01303-7 (PMC7568423; doi:10.1186/s12890-020-01303-7)
Supplement: Supplementary file 1 — Additional file 1. [file 12890_2020_1303_MOESM1_ESM.pdf]

Supplemental Figures

Supplemental Figure S1. GO-terms of biological process enrichment analysis for 228 identified genes. The red vertical line represents that the threshold of the P-value after the Bonferroni step down correction.

Supplemental Figure S2. Violin plots show the expression profiles of identified genes among control, notSA, and SA group. a) for *STAT6*, b) for *MPI*, c) for *GNGT2*, d) for *DECR2*.

Supplemental Figure S3. Boxplots show the differential gene expressions between vehicle and various glucocorticoids including VSG158, VSG159, FF, and DEX. a) for stat6, b) for Decr2, c) for Slc22a5, d) for Lnpep, e) for Ttc19, f) for Mpi.

Supplemental Figure S4. Co-expression patterns of identified genes between vehicle and various glucocorticoids including VSG158, VSG159, FF, and DEX. a) Co-expression patterns of identified genes in vehicle group; b) Co-expression patterns of identified genes in DEX; c) Co-expression patterns of identified genes in FF; d) Co-expression patterns of identified genes in VSG158; e) Co-expression patterns of identified genes in VSG159.

Supplemental Figure S5. Regional plot shows risk SNPs mapped into the severe asthma-associated gene of *GNGT2* based on the GWAS dataset of Dataset #1.

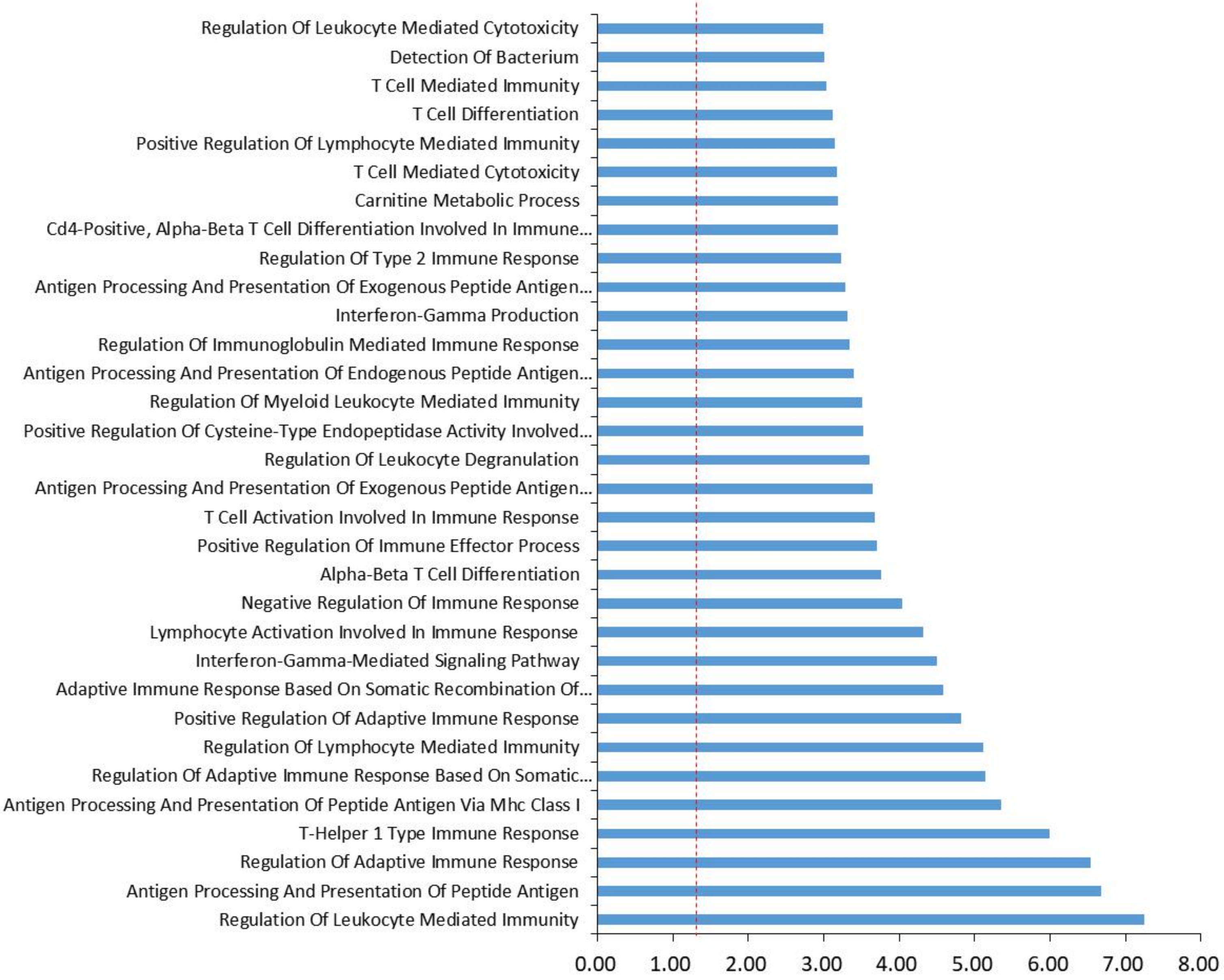

Supplemental Figure S1

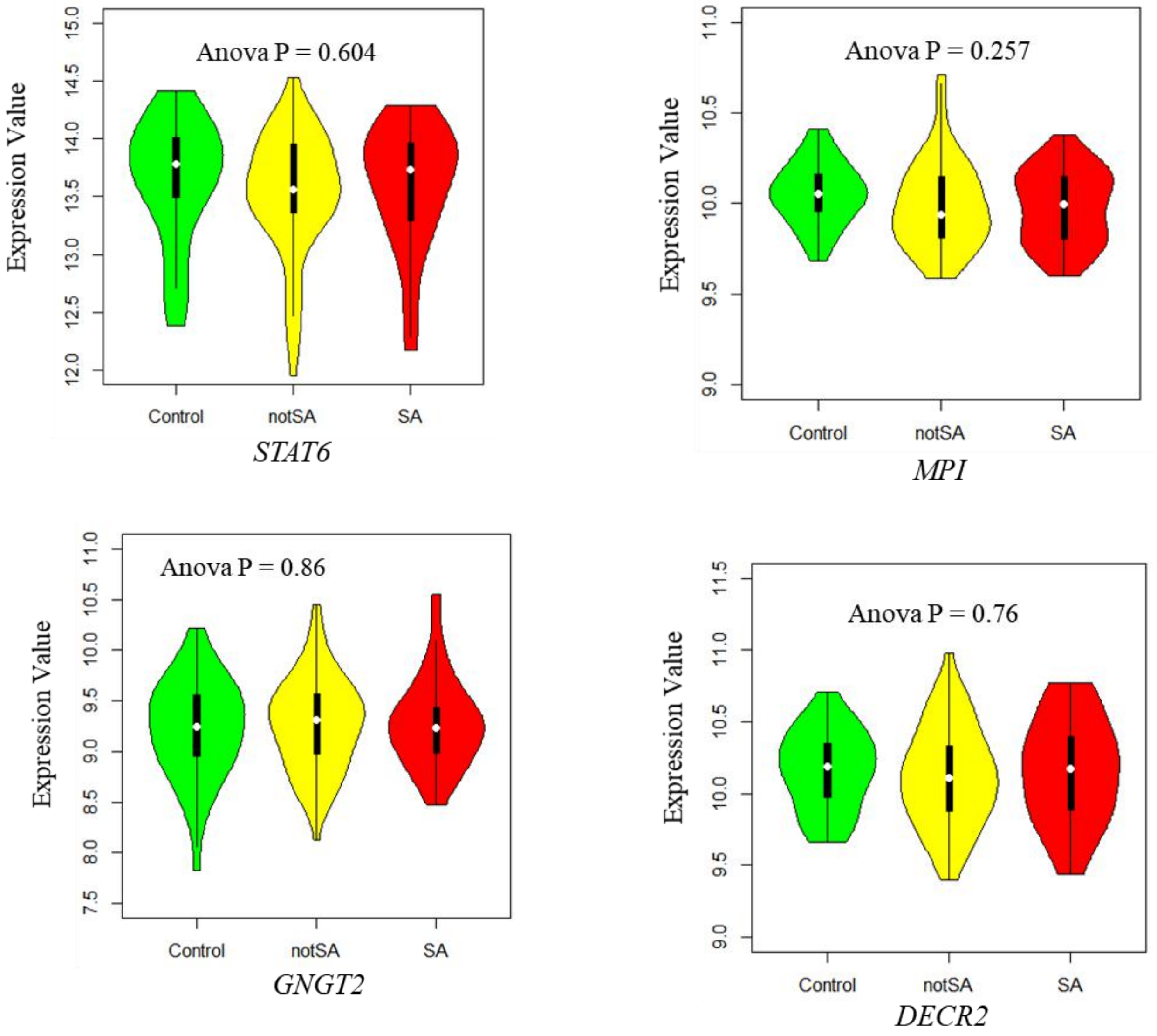

Supplemental Figure S2

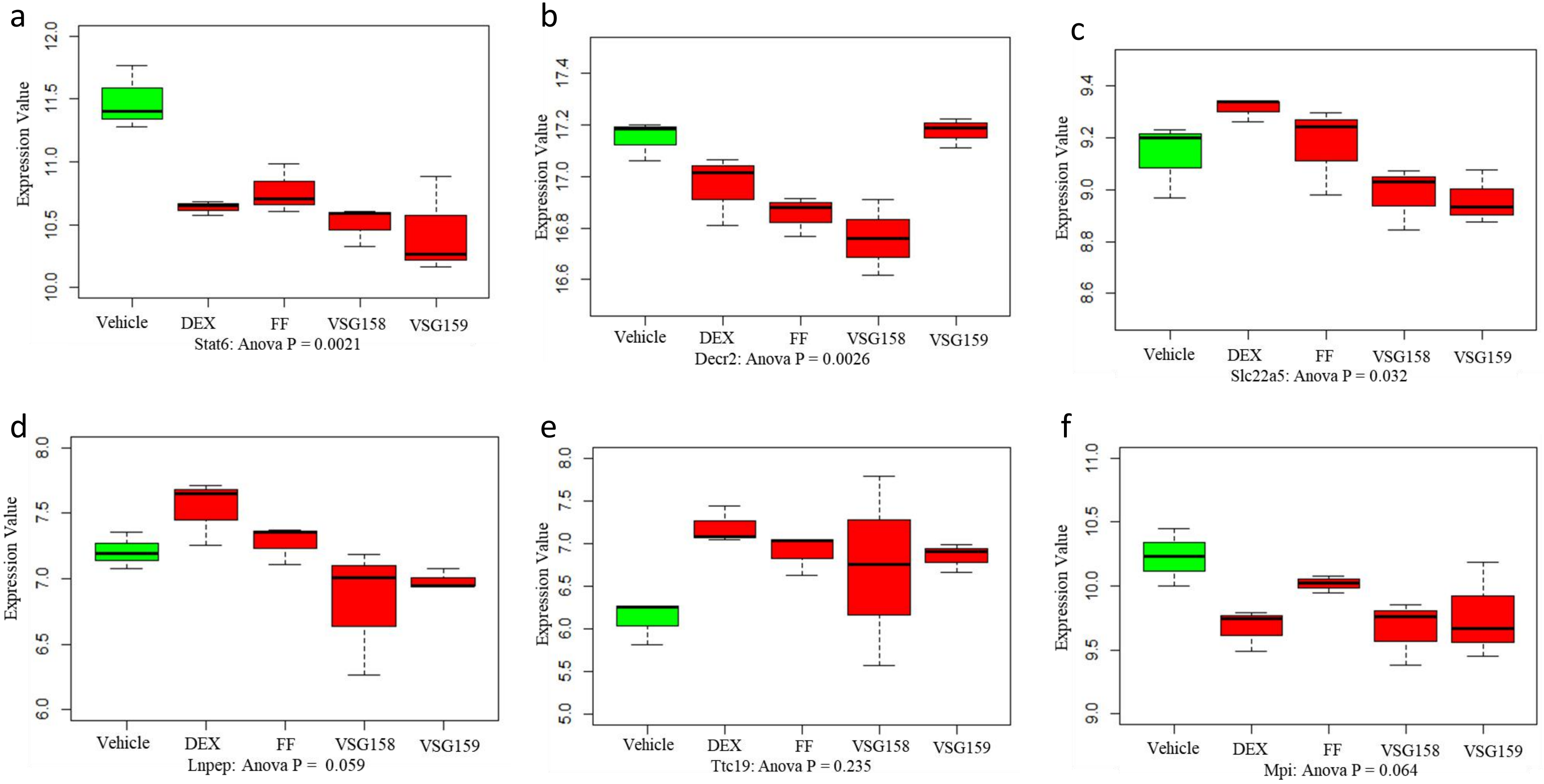

Supplemental Figure S3

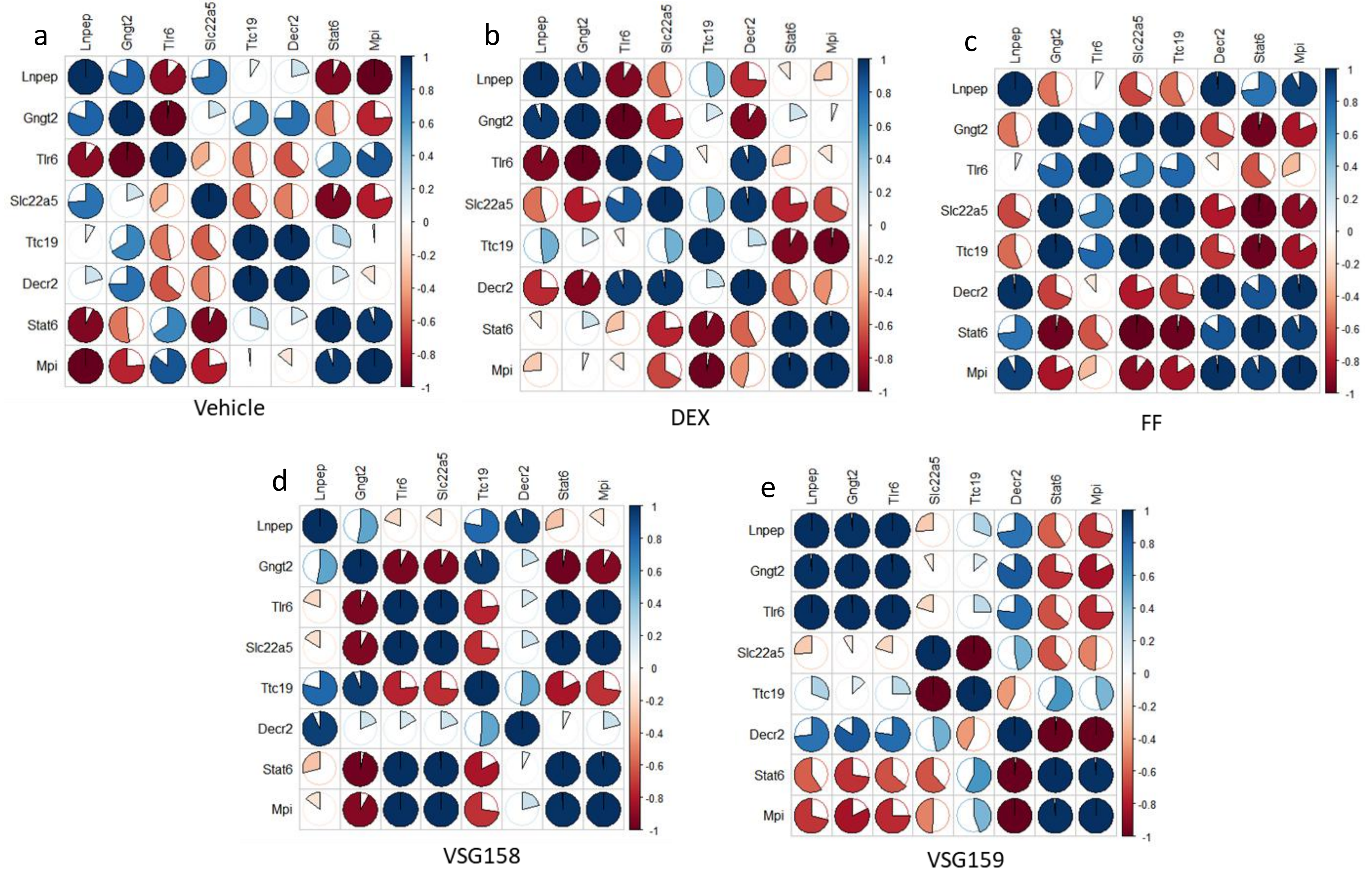

Supplemental Figure S4

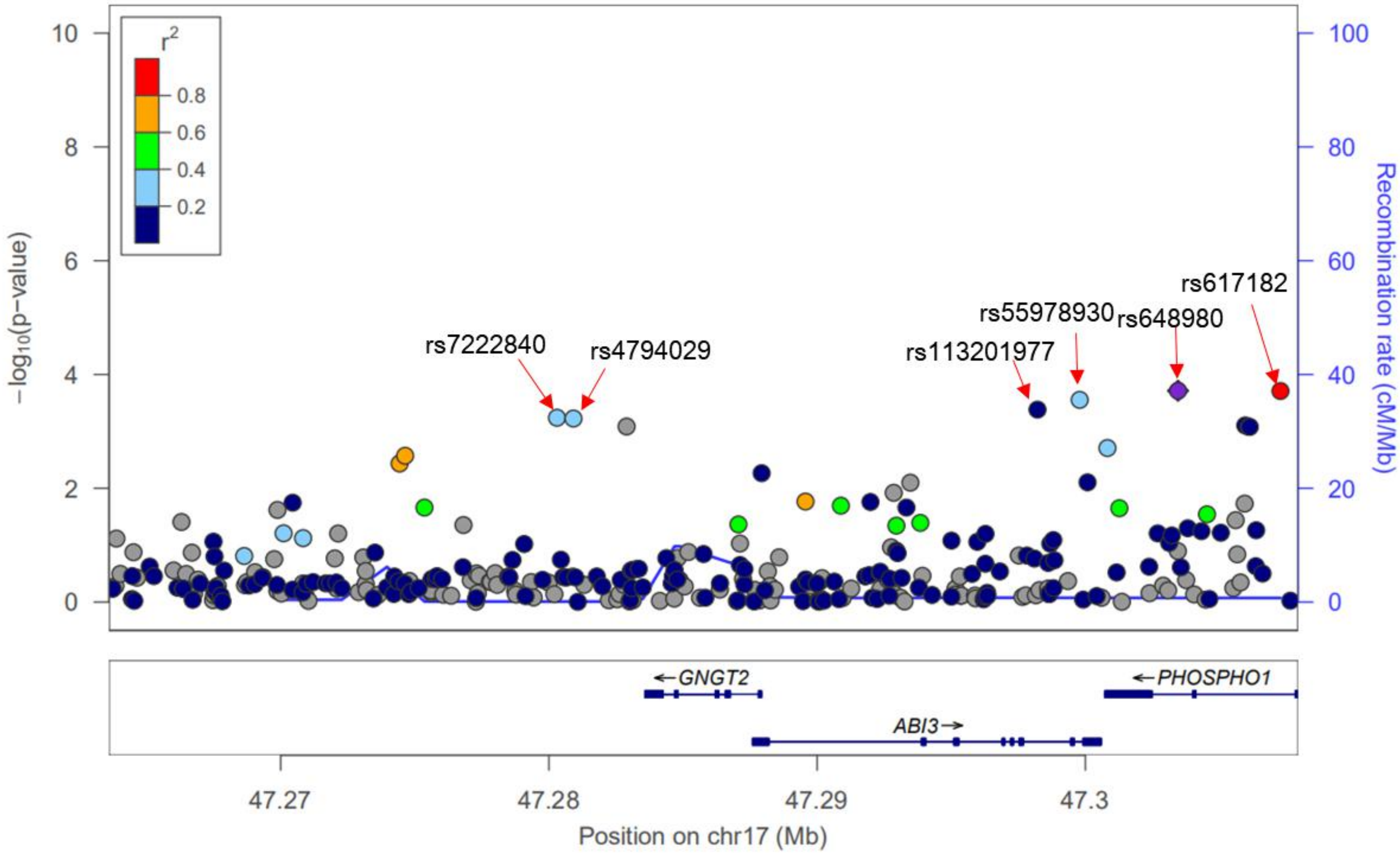

Supplemental Figure S5
