# Supplementary material for: Integrated genomics analysis highlights important SNPs and genes implicated in moderate-to-severe asthma based on GWAS and eQTL datasets
Source: BMC Pulm Med. 2020 Oct 16;20:270. doi: 10.1186/s12890-020-01303-7 (PMC7568423; doi:10.1186/s12890-020-01303-7)
Supplement: Supplementary file 2 — Additional file 2. [file 12890_2020_1303_MOESM2_ESM.docx]

**Supplemental Tables**

**Supplemental Table S1**. **Sherlock Bayesian analysis identifies 1,129 genes as severe asthma-risk genes in discovery samples (Dataset #3)**

| **Gene** | **LBF** | **Sherlock-based P-value** | **GWAS Catalog** |
| --- | --- | --- | --- |
| *HLA-DRB3* | 12.77 | 7.93E-07 | Not documented gene |
| *HLA-DRB5* | 10.67 | 7.93E-07 | Documented gene |
| *HLA-DRB4* | 10.14 | 7.93E-07 | Not documented gene |
| *HLA-DRB1* | 8.87 | 7.93E-07 | Documented gene |
| *RBM43* | 8.12 | 1.59E-06 | Not documented gene |
| *HLA-DOB* | 7.86 | 1.59E-06 | Not documented gene |
| *IKZF3* | 7.17 | 1.59E-06 | Documented gene |
| *IL18R1* | 7.17 | 1.59E-06 | Documented gene |
| *DEXI* | 7.10 | 1.59E-06 | Not documented gene |
| *SUOX* | 7.08 | 3.17E-06 | Documented gene |
| *RERE* | 6.89 | 4.76E-06 | Documented gene |
| *GNGT2* | 6.34 | 1.11E-05 | Documented gene |
| *HLA-DMA* | 6.03 | 1.90E-05 | Not documented gene |
| *SLC22A4* | 5.99 | 1.90E-05 | Not documented gene |
| *MAP2K5* | 5.83 | 2.54E-05 | Not documented gene |
| *TGIF2* | 5.78 | 2.69E-05 | Not documented gene |
| *HLA-DQA1* | 5.74 | 2.85E-05 | Documented gene |
| *KRT83* | 5.57 | 3.80E-05 | Not documented gene |
| *NDFIP1* | 5.57 | 3.80E-05 | Documented gene |
| *PERLD1* | 5.53 | 4.12E-05 | Not documented gene |
| *IER3* | 5.45 | 4.76E-05 | Not documented gene |
| *D2HGDH* | 5.43 | 5.07E-05 | Documented gene |
| *ID2* | 5.36 | 5.39E-05 | Not documented gene |
| *H6PD* | 4.98 | 9.51E-05 | Not documented gene |
| *MBNL1* | 4.94 | 1.01E-04 | Not documented gene |
| *SSRP1* | 4.91 | 1.05E-04 | Not documented gene |
| *CD1E* | 4.90 | 1.08E-04 | Not documented gene |
| *CIC* | 4.89 | 1.08E-04 | Not documented gene |
| *LGALS3BP* | 4.84 | 1.16E-04 | Not documented gene |
| *EFEMP2* | 4.84 | 1.17E-04 | Not documented gene |
| *ZNF76* | 4.82 | 1.17E-04 | Not documented gene |
| *MBIP* | 4.80 | 1.19E-04 | Not documented gene |
| *RPS26* | 4.74 | 1.27E-04 | Documented gene |
| *BSND* | 4.71 | 1.35E-04 | Not documented gene |
| *FBLN2* | 4.70 | 1.35E-04 | Not documented gene |
| *FES* | 4.66 | 1.43E-04 | Not documented gene |
| *CLK3* | 4.65 | 1.44E-04 | Not documented gene |
| *ZNF673* | 4.58 | 1.62E-04 | Not documented gene |
| *C1D* | 4.54 | 1.70E-04 | Not documented gene |
| *MUC13* | 4.53 | 1.76E-04 | Not documented gene |
| *STAT2* | 4.53 | 1.76E-04 | Not documented gene |
| *ZNF20* | 4.52 | 1.78E-04 | Not documented gene |
| *TUBB2A* | 4.51 | 1.81E-04 | Not documented gene |
| *SLC22A5* | 4.49 | 1.85E-04 | Documented gene |
| *RPA2* | 4.48 | 1.85E-04 | Not documented gene |
| *GPBAR1* | 4.47 | 1.85E-04 | Not documented gene |
| *ATP6V0E2* | 4.45 | 1.90E-04 | Not documented gene |
| *PPP2R1B* | 4.45 | 1.92E-04 | Not documented gene |
| *PNKD* | 4.44 | 1.92E-04 | Not documented gene |
| *CCBL1* | 4.44 | 1.92E-04 | Not documented gene |
| *AOC2* | 4.44 | 1.92E-04 | Not documented gene |
| *TOMM40L* | 4.43 | 1.95E-04 | Not documented gene |
| *MGAT4A* | 4.39 | 2.06E-04 | Not documented gene |
| *MFN2* | 4.36 | 2.16E-04 | Not documented gene |
| *STAT6* | 4.35 | 2.19E-04 | Documented gene |
| *RTCD1* | 4.33 | 2.20E-04 | Not documented gene |
| *NPAT* | 4.32 | 2.27E-04 | Not documented gene |
| *N6AMT1* | 4.31 | 2.28E-04 | Not documented gene |
| *ENDOG* | 4.29 | 2.39E-04 | Not documented gene |
| *DEF8* | 4.26 | 2.57E-04 | Not documented gene |
| *SLC22A17* | 4.20 | 2.90E-04 | Not documented gene |
| *KCTD11* | 4.10 | 3.60E-04 | Not documented gene |
| *CDC42SE2* | 4.09 | 3.61E-04 | Not documented gene |
| *CAMK2D* | 4.05 | 3.76E-04 | Not documented gene |
| *TMEM4* | 4.04 | 3.88E-04 | Not documented gene |
| *CD160* | 4.02 | 3.98E-04 | Not documented gene |
| *AOAH* | 4.02 | 3.98E-04 | Not documented gene |
| *UBE2J1* | 4.02 | 3.99E-04 | Not documented gene |
| *MADCAM1* | 3.98 | 4.34E-04 | Not documented gene |
| *MYO5C* | 3.93 | 4.72E-04 | Not documented gene |
| *ZNF273* | 3.91 | 4.82E-04 | Not documented gene |
| *RTN1* | 3.91 | 4.88E-04 | Not documented gene |
| *EBI2* | 3.90 | 5.02E-04 | Not documented gene |
| *ZNF473* | 3.89 | 5.22E-04 | Not documented gene |
| *ZNF672* | 3.88 | 5.23E-04 | Not documented gene |
| *PDGFD* | 3.84 | 5.44E-04 | Not documented gene |
| *CTDSPL2* | 3.81 | 5.67E-04 | Not documented gene |
| *NR2F2* | 3.80 | 5.67E-04 | Not documented gene |
| *CTAGE6* | 3.79 | 5.77E-04 | Not documented gene |
| *TINP1* | 3.75 | 6.13E-04 | Not documented gene |
| *ERG* | 3.72 | 6.31E-04 | Not documented gene |
| *HCG27* | 3.72 | 6.34E-04 | Not documented gene |
| *SLC11A1* | 3.69 | 6.86E-04 | Not documented gene |
| *FCER1G* | 3.64 | 7.29E-04 | Documented gene |
| *DEF6* | 3.61 | 7.53E-04 | Not documented gene |
| *CEP63* | 3.53 | 8.32E-04 | Not documented gene |
| *B9D2* | 3.52 | 8.45E-04 | Not documented gene |
| *P2RY1* | 3.51 | 8.59E-04 | Not documented gene |
| *FAM86A* | 3.49 | 8.78E-04 | Not documented gene |
| *DHX35* | 3.49 | 8.86E-04 | Not documented gene |
| *RPS26P10* | 3.47 | 9.00E-04 | Not documented gene |
| *SEPHS1* | 3.46 | 9.04E-04 | Not documented gene |
| *BRUNOL6* | 3.46 | 9.05E-04 | Not documented gene |
| *CCDC4* | 3.46 | 9.07E-04 | Not documented gene |
| *ULK3* | 3.44 | 9.23E-04 | Not documented gene |
| *TCN2* | 3.44 | 9.24E-04 | Not documented gene |
| *VARS2* | 3.37 | 1.01E-03 | Not documented gene |
| *NFYA* | 3.37 | 1.01E-03 | Not documented gene |
| *RASSF1* | 3.37 | 1.01E-03 | Not documented gene |
| *DUSP18* | 3.36 | 1.02E-03 | Not documented gene |
| *USP35* | 3.34 | 1.06E-03 | Not documented gene |
| *FADS2* | 3.34 | 1.06E-03 | Documented gene |
| *CAPZB* | 3.30 | 1.10E-03 | Not documented gene |
| *ZNF667* | 3.28 | 1.13E-03 | Not documented gene |
| *PROK2* | 3.27 | 1.15E-03 | Not documented gene |
| *NIPSNAP3B* | 3.26 | 1.15E-03 | Not documented gene |
| *NCKIPSD* | 3.21 | 1.23E-03 | Not documented gene |
| *SLC25A17* | 3.19 | 1.26E-03 | Not documented gene |
| *PRMT5* | 3.19 | 1.26E-03 | Not documented gene |
| *PLEKHN1* | 3.19 | 1.27E-03 | Not documented gene |
| *RPS26L1* | 3.18 | 1.28E-03 | Not documented gene |
| *NUMBL* | 3.16 | 1.33E-03 | Not documented gene |
| *HLA-DRB6* | 3.15 | 1.35E-03 | Documented gene |
| *SUSD3* | 3.15 | 1.35E-03 | Not documented gene |
| *PLA2G10* | 3.14 | 1.36E-03 | Not documented gene |
| *ALG5* | 3.14 | 1.36E-03 | Not documented gene |
| *SDF4* | 3.14 | 1.36E-03 | Not documented gene |
| *SACM1L* | 3.13 | 1.39E-03 | Not documented gene |
| *PGM1* | 3.11 | 1.43E-03 | Not documented gene |
| *NSMCE1* | 3.11 | 1.43E-03 | Not documented gene |
| *FCER1A* | 3.09 | 1.47E-03 | Not documented gene |
| *PCNXL2* | 3.07 | 1.51E-03 | Not documented gene |
| *ZNF615* | 3.05 | 1.55E-03 | Not documented gene |
| *RPS27L* | 3.03 | 1.60E-03 | Not documented gene |
| *ZNF576* | 3.02 | 1.61E-03 | Not documented gene |
| *CEP192* | 3.01 | 1.64E-03 | Not documented gene |
| *RAB18* | 3.01 | 1.64E-03 | Documented gene |
| *MITD1* | 3.01 | 1.65E-03 | Not documented gene |
| *MTERFD1* | 2.98 | 1.69E-03 | Not documented gene |
| *AADACL1* | 2.97 | 1.71E-03 | Not documented gene |
| *LYG1* | 2.95 | 1.75E-03 | Not documented gene |
| *TSGA10* | 2.95 | 1.76E-03 | Not documented gene |
| *SNORD73A* | 2.94 | 1.77E-03 | Not documented gene |
| *RPL10A* | 2.91 | 1.84E-03 | Not documented gene |
| *ATP2B1* | 2.91 | 1.85E-03 | Not documented gene |
| *DHRSX* | 2.90 | 1.87E-03 | Not documented gene |
| *ATAD3A* | 2.90 | 1.89E-03 | Not documented gene |
| *ELL3* | 2.89 | 1.89E-03 | Not documented gene |
| *PHF1* | 2.89 | 1.90E-03 | Not documented gene |
| *MPI* | 2.89 | 1.90E-03 | Not documented gene |
| *KIF16B* | 2.86 | 1.98E-03 | Documented gene |
| *DAGLA* | 2.86 | 1.98E-03 | Not documented gene |
| *GLMN* | 2.84 | 2.03E-03 | Not documented gene |
| *TMEM16J* | 2.82 | 2.11E-03 | Not documented gene |
| *TNS4* | 2.81 | 2.12E-03 | Not documented gene |
| *ZNF559* | 2.80 | 2.13E-03 | Not documented gene |
| *SCAMP2* | 2.80 | 2.14E-03 | Not documented gene |
| *WDR37* | 2.78 | 2.21E-03 | Not documented gene |
| *NLK* | 2.77 | 2.25E-03 | Not documented gene |
| *ZBTB2* | 2.77 | 2.25E-03 | Not documented gene |
| *TNNT2* | 2.74 | 2.31E-03 | Not documented gene |
| *GLB1* | 2.74 | 2.32E-03 | Documented gene |
| *ZNF469* | 2.73 | 2.34E-03 | Not documented gene |
| *FEZ2* | 2.72 | 2.39E-03 | Not documented gene |
| *TBCCD1* | 2.71 | 2.40E-03 | Not documented gene |
| *PDIA6* | 2.71 | 2.41E-03 | Not documented gene |
| *RBM18* | 2.69 | 2.44E-03 | Not documented gene |
| *PIK3R3* | 2.69 | 2.46E-03 | Not documented gene |
| *CACNA1H* | 2.68 | 2.47E-03 | Not documented gene |
| *HMGB1* | 2.68 | 2.48E-03 | Not documented gene |
| *SPRYD4* | 2.67 | 2.50E-03 | Not documented gene |
| *CMPK1* | 2.65 | 2.56E-03 | Not documented gene |
| *HCST* | 2.65 | 2.60E-03 | Not documented gene |
| *ALDH7A1* | 2.63 | 2.64E-03 | Documented gene |
| *DENND2C* | 2.63 | 2.64E-03 | Not documented gene |
| *PDAP1* | 2.61 | 2.72E-03 | Not documented gene |
| *LCOR* | 2.61 | 2.74E-03 | Not documented gene |
| *ZNF57* | 2.59 | 2.80E-03 | Not documented gene |
| *NPTN* | 2.59 | 2.82E-03 | Not documented gene |
| *SLC38A2* | 2.57 | 2.91E-03 | Not documented gene |
| *ZZEF1* | 2.56 | 2.92E-03 | Not documented gene |
| *EARS2* | 2.56 | 2.93E-03 | Not documented gene |
| *SPINK2* | 2.56 | 2.94E-03 | Not documented gene |
| *RUNX3* | 2.53 | 3.06E-03 | Documented gene |
| *VPS26* | 2.53 | 3.09E-03 | Not documented gene |
| *CDK9* | 2.52 | 3.10E-03 | Not documented gene |
| *7-Sep* | 2.52 | 3.12E-03 | Not documented gene |
| *GEM* | 2.52 | 3.13E-03 | Not documented gene |
| *LIPT1* | 2.52 | 3.13E-03 | Not documented gene |
| *CTSK* | 2.51 | 3.18E-03 | Not documented gene |
| *SMC4* | 2.51 | 3.19E-03 | Not documented gene |
| *FAM80B* | 2.50 | 3.21E-03 | Not documented gene |
| *SPIB* | 2.50 | 3.22E-03 | Not documented gene |
| *OVCA2* | 2.49 | 3.22E-03 | Not documented gene |
| *MINK1* | 2.49 | 3.24E-03 | Not documented gene |
| *INGX* | 2.46 | 3.36E-03 | Not documented gene |
| *SGCB* | 2.45 | 3.40E-03 | Not documented gene |
| *SLC25A37* | 2.45 | 3.42E-03 | Not documented gene |
| *NAP1L1* | 2.45 | 3.42E-03 | Not documented gene |
| *CYP17A1* | 2.45 | 3.44E-03 | Not documented gene |
| *STARD3* | 2.44 | 3.47E-03 | Not documented gene |
| *ZNF23* | 2.44 | 3.48E-03 | Not documented gene |
| *KLHL17* | 2.44 | 3.48E-03 | Not documented gene |
| *IL23A* | 2.44 | 3.48E-03 | Not documented gene |
| *H2AFV* | 2.43 | 3.49E-03 | Not documented gene |
| *VPS4A* | 2.43 | 3.50E-03 | Not documented gene |
| *MFSD5* | 2.43 | 3.51E-03 | Not documented gene |
| *SNORA62* | 2.43 | 3.51E-03 | Not documented gene |
| *BTBD15* | 2.43 | 3.52E-03 | Not documented gene |
| *PRSS3* | 2.42 | 3.54E-03 | Not documented gene |
| *TMEM177* | 2.42 | 3.56E-03 | Not documented gene |
| *GAS1* | 2.42 | 3.57E-03 | Documented gene |
| *TLR6* | 2.40 | 3.63E-03 | Documented gene |
| *SPPL2B* | 2.40 | 3.66E-03 | Not documented gene |
| *CFHR1* | 2.39 | 3.68E-03 | Not documented gene |
| *DEFB1* | 2.36 | 3.80E-03 | Not documented gene |
| *CENPB* | 2.35 | 3.86E-03 | Not documented gene |
| *RDH11* | 2.35 | 3.87E-03 | Not documented gene |
| *CDH23* | 2.35 | 3.87E-03 | Not documented gene |
| *CRNN* | 2.35 | 3.87E-03 | Not documented gene |
| *HORMAD1* | 2.34 | 3.93E-03 | Not documented gene |
| *NCF1C* | 2.34 | 3.95E-03 | Not documented gene |
| *HERPUD2* | 2.32 | 4.06E-03 | Not documented gene |
| *REV1* | 2.32 | 4.08E-03 | Not documented gene |
| *FOXD4* | 2.32 | 4.08E-03 | Not documented gene |
| *OR52H1* | 2.31 | 4.11E-03 | Not documented gene |
| *ST3GAL5* | 2.31 | 4.11E-03 | Not documented gene |
| *ALMS1* | 2.29 | 4.18E-03 | Not documented gene |
| *RIMS4* | 2.29 | 4.19E-03 | Not documented gene |
| *LRP12* | 2.29 | 4.19E-03 | Not documented gene |
| *MRPL21* | 2.27 | 4.29E-03 | Not documented gene |
| *HECTD2* | 2.27 | 4.31E-03 | Not documented gene |
| *DHX29* | 2.26 | 4.34E-03 | Not documented gene |
| *DNAJC7* | 2.26 | 4.36E-03 | Not documented gene |
| *BDNF* | 2.26 | 4.36E-03 | Not documented gene |
| *IL27RA* | 2.25 | 4.39E-03 | Not documented gene |
| *CENPN* | 2.25 | 4.39E-03 | Not documented gene |
| *RDH5* | 2.25 | 4.44E-03 | Not documented gene |
| *FAM20B* | 2.23 | 4.51E-03 | Not documented gene |
| *ZC3H10* | 2.23 | 4.53E-03 | Not documented gene |
| *UBE2E1* | 2.23 | 4.54E-03 | Not documented gene |
| *RG9MTD3* | 2.22 | 4.60E-03 | Not documented gene |
| *HSPA1A* | 2.21 | 4.63E-03 | Not documented gene |
| *KPNA4* | 2.21 | 4.65E-03 | Not documented gene |
| *NRBP2* | 2.20 | 4.71E-03 | Not documented gene |
| *WDR44* | 2.19 | 4.76E-03 | Not documented gene |
| *ARHGEF9* | 2.19 | 4.78E-03 | Not documented gene |
| *PSIP1* | 2.18 | 4.81E-03 | Not documented gene |
| *FAM132A* | 2.17 | 4.87E-03 | Not documented gene |
| *MED8* | 2.17 | 4.88E-03 | Not documented gene |
| *CC2D2B* | 2.15 | 5.01E-03 | Not documented gene |
| *EPC1* | 2.14 | 5.03E-03 | Not documented gene |
| *NCF1* | 2.14 | 5.03E-03 | Not documented gene |
| *ZNF826* | 2.14 | 5.04E-03 | Not documented gene |
| *TAP1* | 2.14 | 5.07E-03 | Not documented gene |
| *HEXIM2* | 2.13 | 5.09E-03 | Not documented gene |
| *HIST1H2BO* | 2.13 | 5.10E-03 | Not documented gene |
| *MSL3L1* | 2.13 | 5.11E-03 | Not documented gene |
| *OR2A20P* | 2.13 | 5.15E-03 | Not documented gene |
| *CNP* | 2.12 | 5.16E-03 | Not documented gene |
| *MAP4K3* | 2.12 | 5.18E-03 | Not documented gene |
| *CES1* | 2.12 | 5.19E-03 | Not documented gene |
| *BAZ2A* | 2.11 | 5.22E-03 | Not documented gene |
| *HIATL1* | 2.11 | 5.22E-03 | Not documented gene |
| *STK36* | 2.11 | 5.22E-03 | Not documented gene |
| *PSMB2* | 2.11 | 5.27E-03 | Not documented gene |
| *DOK2* | 2.10 | 5.30E-03 | Not documented gene |
| *CTSS* | 2.10 | 5.30E-03 | Not documented gene |
| *NEDD9* | 2.10 | 5.34E-03 | Not documented gene |
| *CRKRS* | 2.10 | 5.34E-03 | Not documented gene |
| *TBX3* | 2.10 | 5.35E-03 | Not documented gene |
| *CLEC3B* | 2.09 | 5.37E-03 | Not documented gene |
| *ERLIN2* | 2.09 | 5.41E-03 | Not documented gene |
| *DUSP10* | 2.08 | 5.43E-03 | Not documented gene |
| *CLYBL* | 2.08 | 5.46E-03 | Not documented gene |
| *AKR1A1* | 2.08 | 5.49E-03 | Not documented gene |
| *MAPKAPK3* | 2.08 | 5.50E-03 | Not documented gene |
| *MFSD1* | 2.07 | 5.51E-03 | Not documented gene |
| *UBE2T* | 2.07 | 5.54E-03 | Not documented gene |
| *THAP4* | 2.07 | 5.56E-03 | Not documented gene |
| *CORO1C* | 2.06 | 5.58E-03 | Not documented gene |
| *PRPF6* | 2.06 | 5.58E-03 | Not documented gene |
| *SLC35E4* | 2.06 | 5.58E-03 | Not documented gene |
| *CDKN1B* | 2.06 | 5.59E-03 | Not documented gene |
| *ZNF74* | 2.06 | 5.64E-03 | Not documented gene |
| *PANX2* | 2.05 | 5.70E-03 | Not documented gene |
| *ANKRD37* | 2.04 | 5.75E-03 | Not documented gene |
| *FBXL3* | 2.03 | 5.85E-03 | Not documented gene |
| *CENTD3* | 2.01 | 5.95E-03 | Not documented gene |
| *MACROD1* | 2.01 | 5.97E-03 | Not documented gene |
| *PCMT1* | 2.01 | 5.98E-03 | Not documented gene |
| *NRSN2* | 2.01 | 5.98E-03 | Not documented gene |
| *TXLNA* | 2.01 | 6.00E-03 | Not documented gene |
| *KCND3* | 2.01 | 6.01E-03 | Not documented gene |
| *PPP3CC* | 2.00 | 6.02E-03 | Not documented gene |
| *RAP1B* | 2.00 | 6.08E-03 | Not documented gene |
| *NUP43* | 1.99 | 6.11E-03 | Not documented gene |
| *LIPH* | 1.98 | 6.18E-03 | Not documented gene |
| *SNORD14A* | 1.98 | 6.23E-03 | Not documented gene |
| *TRIM56* | 1.97 | 6.25E-03 | Not documented gene |
| *PHF5A* | 1.97 | 6.26E-03 | Documented gene |
| *CGRRF1* | 1.97 | 6.34E-03 | Not documented gene |
| *PGCP* | 1.96 | 6.38E-03 | Not documented gene |
| *LAMA2* | 1.96 | 6.39E-03 | Not documented gene |
| *NPHP3* | 1.96 | 6.40E-03 | Not documented gene |
| *ACVR1* | 1.96 | 6.42E-03 | Not documented gene |
| *COQ5* | 1.95 | 6.47E-03 | Not documented gene |
| *BACE2* | 1.94 | 6.57E-03 | Not documented gene |
| *IL4R* | 1.93 | 6.69E-03 | Documented gene |
| *CES4* | 1.93 | 6.71E-03 | Not documented gene |
| *CHODL* | 1.92 | 6.79E-03 | Not documented gene |
| *NBPF12* | 1.92 | 6.79E-03 | Not documented gene |
| *ZNF236* | 1.91 | 6.82E-03 | Not documented gene |
| *SERPINE1* | 1.91 | 6.84E-03 | Not documented gene |
| *NAV1* | 1.91 | 6.87E-03 | Not documented gene |
| *MPV17* | 1.90 | 6.96E-03 | Not documented gene |
| *KCNQ1* | 1.90 | 6.96E-03 | Not documented gene |
| *B3GNT2* | 1.89 | 7.04E-03 | Not documented gene |
| *SNAP29* | 1.88 | 7.06E-03 | Not documented gene |
| *OR6C3* | 1.88 | 7.09E-03 | Not documented gene |
| *CDK7* | 1.88 | 7.14E-03 | Not documented gene |
| *NIP7* | 1.88 | 7.15E-03 | Not documented gene |
| *PRDX3* | 1.88 | 7.16E-03 | Not documented gene |
| *GOLPH3L* | 1.87 | 7.19E-03 | Not documented gene |
| *COPA* | 1.87 | 7.21E-03 | Not documented gene |
| *BMPR1A* | 1.87 | 7.21E-03 | Not documented gene |
| *CDKL1* | 1.87 | 7.25E-03 | Not documented gene |
| *STRBP* | 1.86 | 7.27E-03 | Not documented gene |
| *A1BG* | 1.86 | 7.27E-03 | Not documented gene |
| *CALD1* | 1.86 | 7.28E-03 | Not documented gene |
| *RBM15* | 1.86 | 7.28E-03 | Not documented gene |
| *ARRDC1* | 1.86 | 7.29E-03 | Documented gene |
| *TLK1* | 1.86 | 7.34E-03 | Not documented gene |
| *PMM1* | 1.85 | 7.43E-03 | Not documented gene |
| *HRK* | 1.85 | 7.44E-03 | Not documented gene |
| *THOC5* | 1.84 | 7.47E-03 | Not documented gene |
| *SLC2A6* | 1.84 | 7.55E-03 | Not documented gene |
| *COL17A1* | 1.84 | 7.57E-03 | Not documented gene |
| *GALR2* | 1.84 | 7.60E-03 | Not documented gene |
| *CLK1* | 1.83 | 7.63E-03 | Not documented gene |
| *CYP1B1* | 1.83 | 7.65E-03 | Not documented gene |
| *HSF2* | 1.83 | 7.65E-03 | Not documented gene |
| *WDR24* | 1.83 | 7.66E-03 | Not documented gene |
| *ZNF2* | 1.83 | 7.70E-03 | Not documented gene |
| *TBC1D8* | 1.82 | 7.75E-03 | Not documented gene |
| *HEMGN* | 1.81 | 7.87E-03 | Not documented gene |
| *ZNF181* | 1.80 | 7.98E-03 | Not documented gene |
| *SELL* | 1.80 | 7.99E-03 | Not documented gene |
| *HP1BP3* | 1.80 | 8.00E-03 | Not documented gene |
| *LTBP1* | 1.80 | 8.00E-03 | Not documented gene |
| *KBTBD7* | 1.80 | 8.03E-03 | Not documented gene |
| *TP53* | 1.78 | 8.13E-03 | Not documented gene |
| *LILRB2* | 1.78 | 8.15E-03 | Not documented gene |
| *SPOPL* | 1.78 | 8.18E-03 | Not documented gene |
| *HYAL3* | 1.78 | 8.19E-03 | Not documented gene |
| *MYO7A* | 1.77 | 8.31E-03 | Not documented gene |
| *PELO* | 1.77 | 8.31E-03 | Not documented gene |
| *DNCL1* | 1.76 | 8.33E-03 | Not documented gene |
| *CXCL1* | 1.76 | 8.38E-03 | Not documented gene |
| *WDR1* | 1.76 | 8.40E-03 | Not documented gene |
| *ITIH3* | 1.76 | 8.40E-03 | Not documented gene |
| *TAS2R45* | 1.75 | 8.47E-03 | Not documented gene |
| *TRAPPC6B* | 1.74 | 8.59E-03 | Not documented gene |
| *KLHL2* | 1.74 | 8.61E-03 | Not documented gene |
| *SRPX* | 1.73 | 8.65E-03 | Not documented gene |
| *GEN1* | 1.73 | 8.65E-03 | Not documented gene |
| *ZNF219* | 1.73 | 8.65E-03 | Not documented gene |
| *INDO* | 1.73 | 8.70E-03 | Not documented gene |
| *IREB2* | 1.73 | 8.72E-03 | Not documented gene |
| *FADD* | 1.72 | 8.76E-03 | Not documented gene |
| *PIGL* | 1.72 | 8.77E-03 | Not documented gene |
| *DCLRE1B* | 1.72 | 8.82E-03 | Not documented gene |
| *NEBL* | 1.71 | 8.90E-03 | Not documented gene |
| *TMTC4* | 1.71 | 8.91E-03 | Not documented gene |
| *MED27* | 1.70 | 8.98E-03 | Not documented gene |
| *SLC26A9* | 1.70 | 9.01E-03 | Not documented gene |
| *SPG3A* | 1.69 | 9.12E-03 | Not documented gene |
| *TIMP2* | 1.69 | 9.16E-03 | Not documented gene |
| *UNC50* | 1.68 | 9.23E-03 | Not documented gene |
| *WDR51B* | 1.67 | 9.30E-03 | Not documented gene |
| *SRP54* | 1.67 | 9.32E-03 | Not documented gene |
| *SORD* | 1.67 | 9.37E-03 | Not documented gene |
| *RNF19B* | 1.67 | 9.38E-03 | Not documented gene |
| *PEX19* | 1.66 | 9.43E-03 | Not documented gene |
| *CROP* | 1.65 | 9.52E-03 | Not documented gene |
| *GRAMD4* | 1.65 | 9.54E-03 | Not documented gene |
| *STX4* | 1.65 | 9.57E-03 | Not documented gene |
| *GNL2* | 1.64 | 9.66E-03 | Not documented gene |
| *SNX32* | 1.64 | 9.70E-03 | Not documented gene |
| *EGR3* | 1.63 | 9.76E-03 | Not documented gene |
| *RPS6KA4* | 1.63 | 9.80E-03 | Not documented gene |
| *ICAM5* | 1.62 | 9.86E-03 | Not documented gene |
| *SPTBN1* | 1.62 | 9.87E-03 | Not documented gene |
| *LYPLAL1* | 1.62 | 9.91E-03 | Not documented gene |
| *GNA13* | 1.62 | 9.94E-03 | Not documented gene |
| *PRCP* | 1.62 | 9.97E-03 | Not documented gene |
| *PLEKHA5* | 1.61 | 1.00E-02 | Not documented gene |
| *MAP3K7IP2* | 1.61 | 1.00E-02 | Not documented gene |
| *FBXO30* | 1.61 | 1.00E-02 | Not documented gene |
| *NOX4* | 1.61 | 1.00E-02 | Not documented gene |
| *GADD45A* | 1.60 | 1.02E-02 | Not documented gene |
| *CDH20* | 1.60 | 1.02E-02 | Not documented gene |
| *PPOX* | 1.60 | 1.02E-02 | Not documented gene |
| *IGHMBP2* | 1.59 | 1.03E-02 | Not documented gene |
| *GPR18* | 1.59 | 1.03E-02 | Not documented gene |
| *CBR3* | 1.59 | 1.03E-02 | Not documented gene |
| *LYCAT* | 1.58 | 1.04E-02 | Not documented gene |
| *MRLC2* | 1.58 | 1.04E-02 | Not documented gene |
| *HIST1H2BG* | 1.58 | 1.04E-02 | Not documented gene |
| *AURKAPS1* | 1.58 | 1.04E-02 | Not documented gene |
| *BBS7* | 1.58 | 1.05E-02 | Not documented gene |
| *GSK3B* | 1.57 | 1.05E-02 | Not documented gene |
| *UNQ9370* | 1.57 | 1.06E-02 | Not documented gene |
| *SMAD7* | 1.56 | 1.06E-02 | Not documented gene |
| *ZNRF1* | 1.56 | 1.06E-02 | Not documented gene |
| *BCL6* | 1.54 | 1.08E-02 | Not documented gene |
| *PNMA5* | 1.53 | 1.10E-02 | Not documented gene |
| *ECHDC1* | 1.53 | 1.10E-02 | Not documented gene |
| *ICAM4* | 1.53 | 1.10E-02 | Not documented gene |
| *IRF1* | 1.53 | 1.10E-02 | Not documented gene |
| *RPL28* | 1.52 | 1.11E-02 | Not documented gene |
| *LARP4* | 1.52 | 1.11E-02 | Not documented gene |
| *HDAC2* | 1.52 | 1.11E-02 | Not documented gene |
| *TOM1* | 1.52 | 1.11E-02 | Not documented gene |
| *THRAP5* | 1.51 | 1.12E-02 | Not documented gene |
| *FAM108B1* | 1.51 | 1.13E-02 | Not documented gene |
| *UROC1* | 1.50 | 1.14E-02 | Not documented gene |
| *MKS1* | 1.50 | 1.14E-02 | Not documented gene |
| *ECSIT* | 1.49 | 1.15E-02 | Not documented gene |
| *TNFRSF10D* | 1.49 | 1.15E-02 | Not documented gene |
| *PLCH2* | 1.49 | 1.16E-02 | Not documented gene |
| *TEP1* | 1.49 | 1.16E-02 | Not documented gene |
| *DAP* | 1.49 | 1.16E-02 | Not documented gene |
| *SMARCE1* | 1.49 | 1.16E-02 | Documented gene |
| *OR51E2* | 1.48 | 1.17E-02 | Not documented gene |
| *LRRC20* | 1.48 | 1.18E-02 | Not documented gene |
| *PIK4CA* | 1.48 | 1.18E-02 | Not documented gene |
| *MTRR* | 1.47 | 1.18E-02 | Not documented gene |
| *CXCL14* | 1.47 | 1.19E-02 | Not documented gene |
| *OR2A5* | 1.47 | 1.19E-02 | Not documented gene |
| *NUP205* | 1.47 | 1.19E-02 | Not documented gene |
| *ZNF514* | 1.47 | 1.20E-02 | Not documented gene |
| *FSD1L* | 1.46 | 1.20E-02 | Not documented gene |
| *PSRC1* | 1.46 | 1.21E-02 | Not documented gene |
| *FGR* | 1.46 | 1.21E-02 | Not documented gene |
| *ACYP1* | 1.45 | 1.22E-02 | Not documented gene |
| *ZNF594* | 1.45 | 1.23E-02 | Not documented gene |
| *ADORA2B* | 1.44 | 1.23E-02 | Not documented gene |
| *DUSP13* | 1.44 | 1.23E-02 | Not documented gene |
| *CLPB* | 1.44 | 1.23E-02 | Not documented gene |
| *NGFRAP1* | 1.44 | 1.24E-02 | Not documented gene |
| *ADRA2A* | 1.44 | 1.24E-02 | Not documented gene |
| *SDHB* | 1.44 | 1.24E-02 | Not documented gene |
| *NKAIN3* | 1.43 | 1.25E-02 | Not documented gene |
| *HIST1H2BF* | 1.43 | 1.25E-02 | Not documented gene |
| *SRGAP3* | 1.43 | 1.26E-02 | Not documented gene |
| *ENTPD4* | 1.42 | 1.26E-02 | Not documented gene |
| *SP3* | 1.42 | 1.26E-02 | Not documented gene |
| *PTDSS2* | 1.42 | 1.27E-02 | Not documented gene |
| *CCM2* | 1.42 | 1.27E-02 | Not documented gene |
| *ALOX5* | 1.42 | 1.27E-02 | Documented gene |
| *FCHO2* | 1.42 | 1.27E-02 | Not documented gene |
| *RAB20* | 1.42 | 1.27E-02 | Not documented gene |
| *CYP2A7* | 1.41 | 1.28E-02 | Not documented gene |
| *SHB* | 1.41 | 1.28E-02 | Not documented gene |
| *E4F1* | 1.41 | 1.28E-02 | Not documented gene |
| *ZBTB11* | 1.41 | 1.29E-02 | Not documented gene |
| *EIF3H* | 1.40 | 1.30E-02 | Not documented gene |
| *PAK4* | 1.40 | 1.30E-02 | Not documented gene |
| *PPP1R12A* | 1.39 | 1.32E-02 | Not documented gene |
| *GRB14* | 1.40 | 1.31E-02 | Not documented gene |
| *NOC2L* | 1.39 | 1.32E-02 | Not documented gene |
| *STEAP4* | 1.39 | 1.32E-02 | Not documented gene |
| *ZHX2* | 1.38 | 1.33E-02 | Not documented gene |
| *UBA7* | 1.38 | 1.33E-02 | Not documented gene |
| *CLN6* | 1.38 | 1.33E-02 | Not documented gene |
| *FAM129A* | 1.38 | 1.33E-02 | Not documented gene |
| *PIP5KL1* | 1.38 | 1.33E-02 | Not documented gene |
| *EIF4EBP2* | 1.38 | 1.34E-02 | Not documented gene |
| *TPRKB* | 1.38 | 1.34E-02 | Not documented gene |
| *FAM133B* | 1.37 | 1.35E-02 | Not documented gene |
| *ARPP-19* | 1.37 | 1.36E-02 | Not documented gene |
| *PHIP* | 1.37 | 1.36E-02 | Not documented gene |
| *ZNF154* | 1.36 | 1.36E-02 | Not documented gene |
| *BEXL1* | 1.36 | 1.36E-02 | Not documented gene |
| *CLCN5* | 1.36 | 1.37E-02 | Not documented gene |
| *TMCO6* | 1.36 | 1.37E-02 | Not documented gene |
| *RNPC3* | 1.36 | 1.37E-02 | Not documented gene |
| *UNC13D* | 1.36 | 1.37E-02 | Not documented gene |
| *EIF2B1* | 1.35 | 1.37E-02 | Not documented gene |
| *PARP14* | 1.35 | 1.38E-02 | Not documented gene |
| *CHRNB1* | 1.35 | 1.38E-02 | Not documented gene |
| *GFRA2* | 1.35 | 1.38E-02 | Documented gene |
| *ACTR3* | 1.35 | 1.39E-02 | Not documented gene |
| *CCDC17* | 1.35 | 1.39E-02 | Not documented gene |
| *PBX1* | 1.34 | 1.40E-02 | Not documented gene |
| *ZNF503* | 1.33 | 1.41E-02 | Not documented gene |
| *PSMA5* | 1.32 | 1.43E-02 | Not documented gene |
| *BGN* | 1.32 | 1.43E-02 | Not documented gene |
| *BCAR3* | 1.32 | 1.43E-02 | Not documented gene |
| *ARRDC4* | 1.32 | 1.44E-02 | Not documented gene |
| *MGAT2* | 1.32 | 1.44E-02 | Not documented gene |
| *APOB48R* | 1.31 | 1.45E-02 | Not documented gene |
| *HBB* | 1.31 | 1.45E-02 | Not documented gene |
| *TLR9* | 1.31 | 1.46E-02 | Not documented gene |
| *COG6* | 1.30 | 1.46E-02 | Documented gene |
| *ZFAND2A* | 1.30 | 1.47E-02 | Not documented gene |
| *PMS2L3* | 1.30 | 1.48E-02 | Not documented gene |
| *TGFBI* | 1.30 | 1.48E-02 | Not documented gene |
| *ORAI1* | 1.29 | 1.49E-02 | Not documented gene |
| *MICA* | 1.29 | 1.49E-02 | Not documented gene |
| *ATP10D* | 1.29 | 1.49E-02 | Not documented gene |
| *MTHFD2L* | 1.29 | 1.49E-02 | Not documented gene |
| *KATNA1* | 1.29 | 1.50E-02 | Not documented gene |
| *TTC18* | 1.29 | 1.50E-02 | Not documented gene |
| *PCGF6* | 1.29 | 1.50E-02 | Not documented gene |
| *PMS2L2* | 1.29 | 1.50E-02 | Not documented gene |
| *ATP5B* | 1.28 | 1.50E-02 | Not documented gene |
| *OR7C2* | 1.28 | 1.52E-02 | Not documented gene |
| *NXT2* | 1.28 | 1.52E-02 | Not documented gene |
| *SLC29A1* | 1.27 | 1.52E-02 | Not documented gene |
| *SEMA3F* | 1.27 | 1.53E-02 | Not documented gene |
| *MEI1* | 1.27 | 1.53E-02 | Not documented gene |
| *YIPF5* | 1.27 | 1.53E-02 | Not documented gene |
| *ALG12* | 1.26 | 1.54E-02 | Not documented gene |
| *ARHGAP18* | 1.26 | 1.55E-02 | Not documented gene |
| *SLC25A26* | 1.26 | 1.55E-02 | Not documented gene |
| *UIMC1* | 1.26 | 1.55E-02 | Not documented gene |
| *EIF2C2* | 1.26 | 1.55E-02 | Not documented gene |
| *GTF2H1* | 1.25 | 1.56E-02 | Not documented gene |
| *PKIA* | 1.25 | 1.56E-02 | Not documented gene |
| *GUSBL1* | 1.25 | 1.57E-02 | Not documented gene |
| *AGTPBP1* | 1.24 | 1.58E-02 | Not documented gene |
| *WSB2* | 1.24 | 1.58E-02 | Not documented gene |
| *LAMB2* | 1.24 | 1.59E-02 | Not documented gene |
| *PSMD13* | 1.24 | 1.60E-02 | Not documented gene |
| *SLC20A1* | 1.23 | 1.60E-02 | Not documented gene |
| *MCOLN3* | 1.23 | 1.61E-02 | Not documented gene |
| *UNC45A* | 1.23 | 1.61E-02 | Not documented gene |
| *DECR2* | 1.23 | 1.62E-02 | Not documented gene |
| *TNKS1BP1* | 1.22 | 1.64E-02 | Not documented gene |
| *GDI1* | 1.22 | 1.64E-02 | Not documented gene |
| *FOXRED2* | 1.21 | 1.65E-02 | Not documented gene |
| *SLC9A8* | 1.21 | 1.65E-02 | Not documented gene |
| *PPP3R1* | 1.21 | 1.65E-02 | Not documented gene |
| *RGS12* | 1.21 | 1.65E-02 | Not documented gene |
| *NANS* | 1.21 | 1.66E-02 | Not documented gene |
| *GFI1B* | 1.21 | 1.66E-02 | Not documented gene |
| *ATP11C* | 1.21 | 1.66E-02 | Not documented gene |
| *CHUK* | 1.20 | 1.68E-02 | Not documented gene |
| *ANKRD41* | 1.20 | 1.68E-02 | Not documented gene |
| *IMAA* | 1.20 | 1.68E-02 | Not documented gene |
| *PAPD5* | 1.20 | 1.68E-02 | Not documented gene |
| *TAF7* | 1.20 | 1.69E-02 | Not documented gene |
| *KCNMA1* | 1.19 | 1.69E-02 | Not documented gene |
| *NCOA6IP* | 1.19 | 1.70E-02 | Not documented gene |
| *HECA* | 1.19 | 1.70E-02 | Not documented gene |
| *CYP2W1* | 1.18 | 1.72E-02 | Not documented gene |
| *EIF4E3* | 1.18 | 1.72E-02 | Not documented gene |
| *SSR3* | 1.18 | 1.73E-02 | Documented gene |
| *ANP32C* | 1.18 | 1.73E-02 | Not documented gene |
| *MORG1* | 1.17 | 1.74E-02 | Not documented gene |
| *OBFC2A* | 1.17 | 1.74E-02 | Not documented gene |
| *LRRC57* | 1.17 | 1.75E-02 | Not documented gene |
| *FAM136A* | 1.17 | 1.76E-02 | Not documented gene |
| *GOT1* | 1.16 | 1.76E-02 | Not documented gene |
| *FAM98A* | 1.16 | 1.76E-02 | Not documented gene |
| *BHLHB2* | 1.16 | 1.77E-02 | Not documented gene |
| *4-Sep* | 1.16 | 1.77E-02 | Not documented gene |
| *KLK1* | 1.16 | 1.78E-02 | Not documented gene |
| *TOM1L2* | 1.16 | 1.78E-02 | Not documented gene |
| *LYPD5* | 1.15 | 1.79E-02 | Not documented gene |
| *LLGL2* | 1.15 | 1.79E-02 | Not documented gene |
| *PRPF38B* | 1.15 | 1.79E-02 | Not documented gene |
| *PRMT2* | 1.15 | 1.80E-02 | Not documented gene |
| *SP140* | 1.14 | 1.81E-02 | Not documented gene |
| *TACR3* | 1.14 | 1.81E-02 | Not documented gene |
| *CA5A* | 1.14 | 1.82E-02 | Not documented gene |
| *PLOD1* | 1.14 | 1.83E-02 | Not documented gene |
| *BTF3L4* | 1.14 | 1.83E-02 | Not documented gene |
| *RCSD1* | 1.14 | 1.83E-02 | Not documented gene |
| *DAK* | 1.13 | 1.83E-02 | Not documented gene |
| *SLC15A2* | 1.13 | 1.83E-02 | Not documented gene |
| *TESSP5* | 1.13 | 1.83E-02 | Not documented gene |
| *IFRD1* | 1.13 | 1.85E-02 | Not documented gene |
| *GPR176* | 1.13 | 1.85E-02 | Not documented gene |
| *B3GALNT1* | 1.12 | 1.86E-02 | Not documented gene |
| *ST8SIA6* | 1.12 | 1.86E-02 | Not documented gene |
| *HIST2H2BE* | 1.12 | 1.87E-02 | Not documented gene |
| *ATXN7L3* | 1.12 | 1.87E-02 | Not documented gene |
| *MMD* | 1.11 | 1.88E-02 | Not documented gene |
| *NHEDC2* | 1.11 | 1.88E-02 | Not documented gene |
| *ABHD14B* | 1.11 | 1.88E-02 | Not documented gene |
| *TANK* | 1.11 | 1.88E-02 | Not documented gene |
| *EIF3I* | 1.11 | 1.89E-02 | Not documented gene |
| *CIITA* | 1.11 | 1.89E-02 | Not documented gene |
| *GPR35* | 1.11 | 1.90E-02 | Not documented gene |
| *CCL20* | 1.10 | 1.90E-02 | Documented gene |
| *LRRK2* | 1.10 | 1.90E-02 | Not documented gene |
| *COL12A1* | 1.10 | 1.90E-02 | Documented gene |
| *MTPN* | 1.10 | 1.91E-02 | Not documented gene |
| *CCS* | 1.10 | 1.91E-02 | Not documented gene |
| *SOCS2* | 1.10 | 1.91E-02 | Not documented gene |
| *ABHD12B* | 1.10 | 1.91E-02 | Not documented gene |
| *RDH8* | 1.09 | 1.92E-02 | Not documented gene |
| *NCF1B* | 1.09 | 1.92E-02 | Not documented gene |
| *ACAT2* | 1.09 | 1.93E-02 | Not documented gene |
| *PNOC* | 1.09 | 1.93E-02 | Not documented gene |
| *MT1H* | 1.09 | 1.93E-02 | Not documented gene |
| *TRNT1* | 1.09 | 1.93E-02 | Not documented gene |
| *LCN2* | 1.09 | 1.93E-02 | Not documented gene |
| *MAGEB6B* | 1.09 | 1.94E-02 | Not documented gene |
| *EBI3* | 1.09 | 1.94E-02 | Not documented gene |
| *PSMD8* | 1.08 | 1.95E-02 | Not documented gene |
| *BACE1* | 1.08 | 1.95E-02 | Not documented gene |
| *IFRD2* | 1.08 | 1.95E-02 | Not documented gene |
| *MUTED* | 1.08 | 1.96E-02 | Not documented gene |
| *MT2A* | 1.08 | 1.96E-02 | Not documented gene |
| *ARFIP1* | 1.08 | 1.96E-02 | Not documented gene |
| *HARS2* | 1.08 | 1.97E-02 | Not documented gene |
| *HOXB6* | 1.08 | 1.97E-02 | Not documented gene |
| *RRAGD* | 1.07 | 1.98E-02 | Not documented gene |
| *OR5D16* | 1.07 | 1.98E-02 | Not documented gene |
| *HNRNPL* | 1.07 | 1.99E-02 | Not documented gene |
| *NHP2L1* | 1.06 | 2.01E-02 | Not documented gene |
| *TRPV4* | 1.06 | 2.01E-02 | Not documented gene |
| *FAM54A* | 1.06 | 2.01E-02 | Not documented gene |
| *CDC42EP1* | 1.06 | 2.01E-02 | Not documented gene |
| *IDH2* | 1.05 | 2.03E-02 | Not documented gene |
| *ACOT11* | 1.05 | 2.03E-02 | Not documented gene |
| *TSPYL2* | 1.05 | 2.04E-02 | Not documented gene |
| *ATXN7L1* | 1.04 | 2.06E-02 | Not documented gene |
| *CHST11* | 1.04 | 2.06E-02 | Not documented gene |
| *SLC46A3* | 1.04 | 2.07E-02 | Not documented gene |
| *FPR3* | 1.04 | 2.08E-02 | Not documented gene |
| *ATP5S* | 1.04 | 2.08E-02 | Not documented gene |
| *PTPN18* | 1.03 | 2.08E-02 | Not documented gene |
| *SPHAR* | 1.03 | 2.09E-02 | Not documented gene |
| *ANP32B* | 1.03 | 2.09E-02 | Not documented gene |
| *UBXD4* | 1.03 | 2.10E-02 | Not documented gene |
| *MFSD2* | 1.03 | 2.10E-02 | Not documented gene |
| *RABEP1* | 1.02 | 2.12E-02 | Not documented gene |
| *EIF2S2* | 1.02 | 2.12E-02 | Not documented gene |
| *WDR55* | 1.02 | 2.14E-02 | Not documented gene |
| *LEP* | 1.01 | 2.14E-02 | Not documented gene |
| *ZNF274* | 1.01 | 2.14E-02 | Not documented gene |
| *TIGD7* | 1.01 | 2.15E-02 | Not documented gene |
| *SFTPB* | 1.01 | 2.15E-02 | Not documented gene |
| *AKAP9* | 1.00 | 2.17E-02 | Not documented gene |
| *PSME3* | 1.00 | 2.17E-02 | Not documented gene |
| *ZNF749* | 1.00 | 2.18E-02 | Not documented gene |
| *KCTD13* | 1.00 | 2.19E-02 | Not documented gene |
| *CD82* | 0.99 | 2.20E-02 | Not documented gene |
| *DEFB107A* | 0.99 | 2.20E-02 | Not documented gene |
| *MCM6* | 0.99 | 2.20E-02 | Not documented gene |
| *PTRH1* | 0.99 | 2.21E-02 | Not documented gene |
| *DGCR6* | 0.99 | 2.21E-02 | Not documented gene |
| *DDX12* | 0.99 | 2.22E-02 | Not documented gene |
| *ZNF397* | 0.98 | 2.25E-02 | Not documented gene |
| *BCL2A1* | 0.97 | 2.26E-02 | Not documented gene |
| *CLIP1* | 0.97 | 2.27E-02 | Not documented gene |
| *KCND1* | 0.97 | 2.27E-02 | Not documented gene |
| *PDF* | 0.97 | 2.28E-02 | Not documented gene |
| *ACTL6A* | 0.97 | 2.28E-02 | Not documented gene |
| *LNPEP* | 0.97 | 2.28E-02 | Not documented gene |
| *ANLN* | 0.97 | 2.28E-02 | Not documented gene |
| *GTSE1* | 0.97 | 2.28E-02 | Not documented gene |
| *RGS2* | 0.97 | 2.29E-02 | Not documented gene |
| *RRAS* | 0.96 | 2.30E-02 | Not documented gene |
| *FOXD4L1* | 0.96 | 2.31E-02 | Not documented gene |
| *TMEM65* | 0.96 | 2.31E-02 | Not documented gene |
| *STK16* | 0.96 | 2.31E-02 | Not documented gene |
| *IFT57* | 0.96 | 2.32E-02 | Not documented gene |
| *EIF1AX* | 0.96 | 2.32E-02 | Not documented gene |
| *HIST2H4A* | 0.96 | 2.32E-02 | Not documented gene |
| *RUVBL1* | 0.96 | 2.32E-02 | Not documented gene |
| *PTCD1* | 0.95 | 2.33E-02 | Not documented gene |
| *BNIP3L* | 0.95 | 2.33E-02 | Not documented gene |
| *SATB2* | 0.95 | 2.33E-02 | Not documented gene |
| *SQLE* | 0.95 | 2.35E-02 | Not documented gene |
| *SP4* | 0.95 | 2.35E-02 | Not documented gene |
| *NEK8* | 0.95 | 2.35E-02 | Not documented gene |
| *IFNA7* | 0.95 | 2.36E-02 | Not documented gene |
| *PLEKHB1* | 0.94 | 2.36E-02 | Not documented gene |
| *AP4B1* | 0.94 | 2.37E-02 | Not documented gene |
| *FAM110A* | 0.94 | 2.37E-02 | Not documented gene |
| *NOL6* | 0.94 | 2.37E-02 | Not documented gene |
| *LRBA* | 0.94 | 2.38E-02 | Not documented gene |
| *SLC35B4* | 0.94 | 2.38E-02 | Not documented gene |
| *PTPRG* | 0.94 | 2.39E-02 | Not documented gene |
| *ATOH8* | 0.93 | 2.39E-02 | Not documented gene |
| *CAPZA1* | 0.93 | 2.39E-02 | Not documented gene |
| *RNF213* | 0.93 | 2.41E-02 | Not documented gene |
| *FAM43A* | 0.92 | 2.43E-02 | Not documented gene |
| *RRS1* | 0.92 | 2.43E-02 | Not documented gene |
| *PLAC8L1* | 0.92 | 2.44E-02 | Not documented gene |
| *AFP* | 0.92 | 2.45E-02 | Not documented gene |
| *ZFP36L1* | 0.92 | 2.46E-02 | Not documented gene |
| *ZNF430* | 0.92 | 2.46E-02 | Not documented gene |
| *UGDH* | 0.92 | 2.46E-02 | Not documented gene |
| *DNTTIP1* | 0.91 | 2.47E-02 | Not documented gene |
| *SKIV2L2* | 0.91 | 2.48E-02 | Not documented gene |
| *ADAMTSL4* | 0.91 | 2.49E-02 | Not documented gene |
| *CCDC27* | 0.91 | 2.49E-02 | Not documented gene |
| *R3HDM1* | 0.90 | 2.50E-02 | Not documented gene |
| *TRIM48* | 0.90 | 2.51E-02 | Not documented gene |
| *TUBA4A* | 0.90 | 2.52E-02 | Not documented gene |
| *CRISPLD2* | 0.90 | 2.52E-02 | Not documented gene |
| *TTC19* | 0.90 | 2.52E-02 | Not documented gene |
| *ECH1* | 0.90 | 2.53E-02 | Not documented gene |
| *PICK1* | 0.89 | 2.53E-02 | Not documented gene |
| *WARS2* | 0.89 | 2.54E-02 | Not documented gene |
| *UROD* | 0.89 | 2.55E-02 | Not documented gene |
| *MAP3K11* | 0.89 | 2.55E-02 | Not documented gene |
| *NSMCE2* | 0.88 | 2.56E-02 | Not documented gene |
| *POLA* | 0.88 | 2.57E-02 | Not documented gene |
| *OPLAH* | 0.88 | 2.58E-02 | Not documented gene |
| *CCT8* | 0.88 | 2.58E-02 | Not documented gene |
| *HSBP1* | 0.87 | 2.60E-02 | Not documented gene |
| *AARS* | 0.87 | 2.60E-02 | Not documented gene |
| *MUTYH* | 0.87 | 2.60E-02 | Not documented gene |
| *CFLAR* | 0.87 | 2.61E-02 | Not documented gene |
| *GGTLC1* | 0.87 | 2.61E-02 | Not documented gene |
| *FAM19A2* | 0.87 | 2.61E-02 | Documented gene |
| *HBA2* | 0.87 | 2.61E-02 | Not documented gene |
| *FAM111B* | 0.87 | 2.62E-02 | Not documented gene |
| *ALPPL2* | 0.87 | 2.63E-02 | Not documented gene |
| *IFNA21* | 0.86 | 2.64E-02 | Not documented gene |
| *SFMBT1* | 0.86 | 2.64E-02 | Not documented gene |
| *CMAH* | 0.86 | 2.64E-02 | Not documented gene |
| *TMEM174* | 0.86 | 2.64E-02 | Not documented gene |
| *SEC16B* | 0.86 | 2.64E-02 | Not documented gene |
| *SFRS16* | 0.86 | 2.64E-02 | Not documented gene |
| *AYP1P1* | 0.86 | 2.65E-02 | Not documented gene |
| *TIPIN* | 0.86 | 2.65E-02 | Not documented gene |
| *KLHL7* | 0.86 | 2.66E-02 | Not documented gene |
| *OTUD1* | 0.85 | 2.67E-02 | Not documented gene |
| *CEP57* | 0.85 | 2.67E-02 | Not documented gene |
| *EIF4E* | 0.85 | 2.67E-02 | Not documented gene |
| *TMC8* | 0.85 | 2.68E-02 | Not documented gene |
| *SUMF1* | 0.85 | 2.68E-02 | Not documented gene |
| *ZNF556* | 0.85 | 2.68E-02 | Not documented gene |
| *FPR1* | 0.85 | 2.69E-02 | Not documented gene |
| *ALPI* | 0.85 | 2.69E-02 | Not documented gene |
| *IARS2* | 0.85 | 2.70E-02 | Not documented gene |
| *KLF1* | 0.84 | 2.71E-02 | Not documented gene |
| *HOXD11* | 0.84 | 2.72E-02 | Not documented gene |
| *AACS* | 0.84 | 2.73E-02 | Not documented gene |
| *ALS2CR13* | 0.84 | 2.74E-02 | Not documented gene |
| *POLR3F* | 0.83 | 2.74E-02 | Not documented gene |
| *SIRT1* | 0.83 | 2.74E-02 | Not documented gene |
| *NME6* | 0.83 | 2.76E-02 | Not documented gene |
| *MRCL3* | 0.83 | 2.78E-02 | Not documented gene |
| *PSD3* | 0.83 | 2.78E-02 | Not documented gene |
| *TSPAN15* | 0.83 | 2.78E-02 | Not documented gene |
| *MLLT1* | 0.82 | 2.79E-02 | Not documented gene |
| *PLCD4* | 0.82 | 2.80E-02 | Not documented gene |
| *NUP210* | 0.82 | 2.82E-02 | Not documented gene |
| *TCF25* | 0.82 | 2.82E-02 | Not documented gene |
| *TRIM69* | 0.81 | 2.82E-02 | Not documented gene |
| *RNF167* | 0.81 | 2.83E-02 | Not documented gene |
| *MAP4K5* | 0.81 | 2.83E-02 | Not documented gene |
| *NAT9* | 0.81 | 2.84E-02 | Not documented gene |
| *MRPL48* | 0.81 | 2.85E-02 | Not documented gene |
| *CLPX* | 0.81 | 2.86E-02 | Not documented gene |
| *POLR2C* | 0.80 | 2.87E-02 | Not documented gene |
| *PPP1R3D* | 0.80 | 2.87E-02 | Not documented gene |
| *ALKBH2* | 0.80 | 2.87E-02 | Not documented gene |
| *ZNF248* | 0.80 | 2.88E-02 | Not documented gene |
| *ZNF213* | 0.80 | 2.90E-02 | Not documented gene |
| *ROCK2* | 0.80 | 2.90E-02 | Not documented gene |
| *ACPT* | 0.79 | 2.90E-02 | Not documented gene |
| *MTMR2* | 0.79 | 2.91E-02 | Not documented gene |
| *IFI35* | 0.79 | 2.91E-02 | Not documented gene |
| *TSC22D1* | 0.79 | 2.91E-02 | Not documented gene |
| *TRMT12* | 0.79 | 2.92E-02 | Not documented gene |
| *TP53INP1* | 0.79 | 2.92E-02 | Not documented gene |
| *RRBP1* | 0.79 | 2.93E-02 | Not documented gene |
| *RANBP2* | 0.79 | 2.93E-02 | Not documented gene |
| *HDGF* | 0.79 | 2.93E-02 | Not documented gene |
| *NQO1* | 0.79 | 2.93E-02 | Not documented gene |
| *LSM11* | 0.78 | 2.95E-02 | Not documented gene |
| *OR2T6* | 0.78 | 2.95E-02 | Not documented gene |
| *GAGE4* | 0.78 | 2.95E-02 | Not documented gene |
| *MCTS1* | 0.78 | 2.96E-02 | Not documented gene |
| *CCAR1* | 0.78 | 2.96E-02 | Not documented gene |
| *SPRY4* | 0.78 | 2.96E-02 | Not documented gene |
| *SHOX2* | 0.78 | 2.97E-02 | Not documented gene |
| *DDX1* | 0.78 | 2.98E-02 | Not documented gene |
| *APBB3* | 0.77 | 2.99E-02 | Not documented gene |
| *OTUD3* | 0.77 | 2.99E-02 | Not documented gene |
| *WDR21A* | 0.77 | 3.00E-02 | Not documented gene |
| *RAB6A* | 0.77 | 3.00E-02 | Not documented gene |
| *NUDCD2* | 0.77 | 3.00E-02 | Not documented gene |
| *TRIM37* | 0.77 | 3.00E-02 | Not documented gene |
| *DCTD* | 0.77 | 3.01E-02 | Not documented gene |
| *PARN* | 0.77 | 3.01E-02 | Not documented gene |
| *TAC3* | 0.77 | 3.01E-02 | Not documented gene |
| *ZBTB26* | 0.77 | 3.01E-02 | Not documented gene |
| *BAZ2B* | 0.77 | 3.01E-02 | Not documented gene |
| *KCTD10* | 0.77 | 3.02E-02 | Not documented gene |
| *ZBTB20* | 0.77 | 3.03E-02 | Not documented gene |
| *RET* | 0.77 | 3.03E-02 | Not documented gene |
| *NDRG2* | 0.77 | 3.03E-02 | Not documented gene |
| *SMPD3* | 0.76 | 3.04E-02 | Not documented gene |
| *FICD* | 0.76 | 3.04E-02 | Not documented gene |
| *VPS39* | 0.76 | 3.05E-02 | Not documented gene |
| *UBE2L6* | 0.76 | 3.05E-02 | Not documented gene |
| *CIB2* | 0.76 | 3.05E-02 | Not documented gene |
| *SGTB* | 0.76 | 3.06E-02 | Not documented gene |
| *EDEM3* | 0.76 | 3.06E-02 | Not documented gene |
| *DRGX* | 0.76 | 3.06E-02 | Not documented gene |
| *NARS2* | 0.76 | 3.06E-02 | Not documented gene |
| *POU2F1* | 0.76 | 3.06E-02 | Not documented gene |
| *CNKSR2* | 0.76 | 3.06E-02 | Not documented gene |
| *LRTM2* | 0.76 | 3.07E-02 | Not documented gene |
| *DCP1A* | 0.75 | 3.07E-02 | Not documented gene |
| *GALT* | 0.75 | 3.07E-02 | Not documented gene |
| *ABCC9* | 0.75 | 3.08E-02 | Not documented gene |
| *SRA1* | 0.75 | 3.08E-02 | Not documented gene |
| *CYP4V2* | 0.75 | 3.08E-02 | Not documented gene |
| *MSI2* | 0.75 | 3.08E-02 | Not documented gene |
| *ETV7* | 0.75 | 3.08E-02 | Not documented gene |
| *COIL* | 0.75 | 3.09E-02 | Not documented gene |
| *BRD2* | 0.75 | 3.09E-02 | Documented gene |
| *ARFGAP2* | 0.75 | 3.10E-02 | Not documented gene |
| *CHI3L2* | 0.75 | 3.10E-02 | Not documented gene |
| *TRIML1* | 0.75 | 3.10E-02 | Not documented gene |
| *TERF2* | 0.75 | 3.11E-02 | Not documented gene |
| *CABP2* | 0.75 | 3.11E-02 | Not documented gene |
| *MRPS6* | 0.75 | 3.11E-02 | Not documented gene |
| *AKAP4* | 0.75 | 3.11E-02 | Not documented gene |
| *DPF2* | 0.74 | 3.12E-02 | Not documented gene |
| *PPIL5* | 0.74 | 3.12E-02 | Not documented gene |
| *CHRNA2* | 0.74 | 3.12E-02 | Documented gene |
| *TMEM175* | 0.74 | 3.13E-02 | Not documented gene |
| *MCL1* | 0.74 | 3.13E-02 | Not documented gene |
| *ETNK1* | 0.74 | 3.14E-02 | Not documented gene |
| *RIN1* | 0.74 | 3.14E-02 | Not documented gene |
| *PSENEN* | 0.73 | 3.17E-02 | Not documented gene |
| *EAF2* | 0.73 | 3.18E-02 | Not documented gene |
| *AK1* | 0.73 | 3.18E-02 | Not documented gene |
| *ZNF148* | 0.73 | 3.18E-02 | Not documented gene |
| *CCDC82* | 0.73 | 3.19E-02 | Not documented gene |
| *SULF2* | 0.73 | 3.19E-02 | Not documented gene |
| *HEY1* | 0.73 | 3.19E-02 | Not documented gene |
| *UBE3B* | 0.73 | 3.20E-02 | Not documented gene |
| *RBM6* | 0.73 | 3.20E-02 | Not documented gene |
| *TMPRSS11F* | 0.73 | 3.20E-02 | Not documented gene |
| *CASD1* | 0.73 | 3.20E-02 | Not documented gene |
| *IMP3* | 0.72 | 3.24E-02 | Not documented gene |
| *LRRC8D* | 0.72 | 3.24E-02 | Not documented gene |
| *SMPDL3A* | 0.72 | 3.24E-02 | Not documented gene |
| *GRAP2* | 0.71 | 3.25E-02 | Not documented gene |
| *GAB2* | 0.71 | 3.25E-02 | Not documented gene |
| *PIP3-E* | 0.71 | 3.25E-02 | Not documented gene |
| *RDH10* | 0.71 | 3.26E-02 | Not documented gene |
| *MAP2K4* | 0.71 | 3.27E-02 | Not documented gene |
| *STAT1* | 0.71 | 3.27E-02 | Not documented gene |
| *GLO1* | 0.71 | 3.27E-02 | Not documented gene |
| *SLC12A3* | 0.71 | 3.27E-02 | Not documented gene |
| *GSTP1* | 0.71 | 3.29E-02 | Not documented gene |
| *BTN2A2* | 0.70 | 3.29E-02 | Not documented gene |
| *FADS6* | 0.70 | 3.29E-02 | Not documented gene |
| *YIF1A* | 0.70 | 3.29E-02 | Not documented gene |
| *SS18L1* | 0.70 | 3.30E-02 | Not documented gene |
| *MGMT* | 0.70 | 3.31E-02 | Not documented gene |
| *GPRIN3* | 0.70 | 3.31E-02 | Not documented gene |
| *CTAGE5* | 0.70 | 3.31E-02 | Not documented gene |
| *PPIF* | 0.70 | 3.31E-02 | Not documented gene |
| *HTR3B* | 0.70 | 3.32E-02 | Not documented gene |
| *CTNNB1* | 0.70 | 3.34E-02 | Not documented gene |
| *GCM1* | 0.70 | 3.34E-02 | Not documented gene |
| *PHOSPHO1* | 0.70 | 3.34E-02 | Not documented gene |
| *UTP3* | 0.69 | 3.35E-02 | Not documented gene |
| *11-Sep* | 0.69 | 3.36E-02 | Not documented gene |
| *MAFG* | 0.69 | 3.36E-02 | Not documented gene |
| *TXNL1* | 0.69 | 3.37E-02 | Not documented gene |
| *XRN2* | 0.69 | 3.38E-02 | Not documented gene |
| *PARP10* | 0.69 | 3.38E-02 | Not documented gene |
| *FIZ1* | 0.68 | 3.39E-02 | Not documented gene |
| *CCDC43* | 0.68 | 3.41E-02 | Not documented gene |
| *GNL1* | 0.68 | 3.41E-02 | Not documented gene |
| *WDR62* | 0.68 | 3.42E-02 | Not documented gene |
| *DOK1* | 0.68 | 3.42E-02 | Not documented gene |
| *HIVEP2* | 0.68 | 3.42E-02 | Not documented gene |
| *TMEM106B* | 0.68 | 3.42E-02 | Not documented gene |
| *LGALS3* | 0.68 | 3.43E-02 | Not documented gene |
| *DNAJC18* | 0.67 | 3.45E-02 | Not documented gene |
| *ERBB3* | 0.67 | 3.45E-02 | Documented gene |
| *SNORA66* | 0.67 | 3.46E-02 | Not documented gene |
| *PRPF4* | 0.67 | 3.47E-02 | Not documented gene |
| *SF3A2* | 0.67 | 3.49E-02 | Not documented gene |
| *PHYH* | 0.66 | 3.49E-02 | Not documented gene |
| *SNTB2* | 0.66 | 3.52E-02 | Not documented gene |
| *ANAPC4* | 0.66 | 3.52E-02 | Not documented gene |
| *XRRA1* | 0.66 | 3.53E-02 | Not documented gene |
| *7-Mar* | 0.66 | 3.53E-02 | Not documented gene |
| *SAMD4A* | 0.66 | 3.54E-02 | Not documented gene |
| *ZNF396* | 0.66 | 3.54E-02 | Not documented gene |
| *KCNK6* | 0.66 | 3.55E-02 | Not documented gene |
| *CSNK2B* | 0.66 | 3.55E-02 | Not documented gene |
| *JOSD3* | 0.65 | 3.55E-02 | Not documented gene |
| *CHST3* | 0.65 | 3.56E-02 | Not documented gene |
| *KLF10* | 0.65 | 3.57E-02 | Not documented gene |
| *LEKR1* | 0.65 | 3.57E-02 | Not documented gene |
| *DOCK10* | 0.65 | 3.59E-02 | Not documented gene |
| *INTS4* | 0.65 | 3.59E-02 | Not documented gene |
| *VSIG6* | 0.65 | 3.59E-02 | Not documented gene |
| *PRR16* | 0.65 | 3.59E-02 | Not documented gene |
| *COL23A1* | 0.64 | 3.60E-02 | Not documented gene |
| *ZNF155* | 0.64 | 3.60E-02 | Not documented gene |
| *CDC42BPB* | 0.64 | 3.62E-02 | Not documented gene |
| *MCM3AP* | 0.64 | 3.62E-02 | Not documented gene |
| *CDC25B* | 0.64 | 3.62E-02 | Not documented gene |
| *PSMB9* | 0.64 | 3.63E-02 | Not documented gene |
| *TBC1D1* | 0.64 | 3.63E-02 | Not documented gene |
| *TFPI* | 0.64 | 3.63E-02 | Not documented gene |
| *TGIF1* | 0.64 | 3.64E-02 | Not documented gene |
| *POLR2B* | 0.64 | 3.64E-02 | Not documented gene |
| *TSPAN2* | 0.64 | 3.64E-02 | Not documented gene |
| *DHX30* | 0.63 | 3.65E-02 | Not documented gene |
| *HERC4* | 0.63 | 3.66E-02 | Not documented gene |
| *LTA* | 0.63 | 3.66E-02 | Not documented gene |
| *SCAND1* | 0.63 | 3.66E-02 | Not documented gene |
| *EGFL7* | 0.63 | 3.66E-02 | Not documented gene |
| *USP43* | 0.63 | 3.66E-02 | Not documented gene |
| *CCNL1* | 0.63 | 3.67E-02 | Not documented gene |
| *BMP1* | 0.63 | 3.68E-02 | Not documented gene |
| *GHITM* | 0.63 | 3.68E-02 | Not documented gene |
| *SLC6A15* | 0.63 | 3.70E-02 | Not documented gene |
| *DTX1* | 0.63 | 3.70E-02 | Not documented gene |
| *C1GALT1* | 0.62 | 3.71E-02 | Not documented gene |
| *SH2B3* | 0.62 | 3.72E-02 | Not documented gene |
| *STIM2* | 0.62 | 3.72E-02 | Not documented gene |
| *BLMH* | 0.62 | 3.73E-02 | Not documented gene |
| *GRINL1A* | 0.62 | 3.74E-02 | Not documented gene |
| *MNAT1* | 0.62 | 3.74E-02 | Not documented gene |
| *OR2T1* | 0.62 | 3.74E-02 | Not documented gene |
| *XCR1* | 0.62 | 3.74E-02 | Not documented gene |
| *EED* | 0.62 | 3.74E-02 | Not documented gene |
| *MECP2* | 0.62 | 3.75E-02 | Not documented gene |
| *RBM7* | 0.62 | 3.75E-02 | Not documented gene |
| *ATP8B1* | 0.62 | 3.75E-02 | Not documented gene |
| *RAMP3* | 0.62 | 3.75E-02 | Not documented gene |
| *KCTD6* | 0.62 | 3.76E-02 | Not documented gene |
| *ADAM8* | 0.61 | 3.78E-02 | Not documented gene |
| *RPL32P3* | 0.61 | 3.78E-02 | Not documented gene |
| *SVIL* | 0.61 | 3.78E-02 | Not documented gene |
| *HEXDC* | 0.61 | 3.79E-02 | Not documented gene |
| *KCNAB1* | 0.61 | 3.80E-02 | Not documented gene |
| *THUMPD1* | 0.61 | 3.80E-02 | Not documented gene |
| *RPH3A* | 0.61 | 3.80E-02 | Not documented gene |
| *TTRAP* | 0.60 | 3.83E-02 | Not documented gene |
| *MAP3K7IP3* | 0.60 | 3.83E-02 | Not documented gene |
| *FCGR1B* | 0.60 | 3.84E-02 | Not documented gene |
| *ALDH9A1* | 0.60 | 3.85E-02 | Not documented gene |
| *ANKRD40* | 0.60 | 3.86E-02 | Not documented gene |
| *B3GALNT2* | 0.59 | 3.88E-02 | Not documented gene |
| *MOSPD1* | 0.59 | 3.89E-02 | Not documented gene |
| *CYB5D1* | 0.59 | 3.90E-02 | Not documented gene |
| *COPG2* | 0.59 | 3.90E-02 | Not documented gene |
| *TYW3* | 0.59 | 3.90E-02 | Not documented gene |
| *IPO8* | 0.59 | 3.90E-02 | Not documented gene |
| *ARL14* | 0.59 | 3.91E-02 | Not documented gene |
| *HLA-C* | 0.59 | 3.92E-02 | Not documented gene |
| *ZFAND3* | 0.59 | 3.92E-02 | Not documented gene |
| *PLD4* | 0.59 | 3.93E-02 | Not documented gene |
| *HAPLN2* | 0.59 | 3.93E-02 | Not documented gene |
| *CHD1L* | 0.59 | 3.94E-02 | Not documented gene |
| *MDC1* | 0.58 | 3.94E-02 | Not documented gene |
| *NAPSB* | 0.58 | 3.96E-02 | Not documented gene |
| *EPAS1* | 0.58 | 3.96E-02 | Not documented gene |
| *SEC15L2* | 0.58 | 3.96E-02 | Not documented gene |
| *TMEM19* | 0.58 | 3.96E-02 | Not documented gene |
| *PPP1R16B* | 0.58 | 3.96E-02 | Not documented gene |
| *BRI3* | 0.58 | 3.97E-02 | Not documented gene |
| *APIP* | 0.58 | 3.97E-02 | Not documented gene |
| *SHROOM1* | 0.58 | 3.97E-02 | Not documented gene |
| *DHX40* | 0.57 | 4.00E-02 | Not documented gene |
| *ALKBH6* | 0.57 | 4.01E-02 | Not documented gene |
| *CHMP2B* | 0.57 | 4.02E-02 | Not documented gene |
| *RTP2* | 0.57 | 4.03E-02 | Not documented gene |
| *CD1A* | 0.57 | 4.03E-02 | Not documented gene |
| *LPAR1* | 0.57 | 4.04E-02 | Not documented gene |
| *ALG13* | 0.57 | 4.05E-02 | Not documented gene |
| *CBX4* | 0.56 | 4.06E-02 | Not documented gene |
| *ALX3* | 0.56 | 4.07E-02 | Not documented gene |
| *LCE1B* | 0.56 | 4.08E-02 | Not documented gene |
| *MYCL1* | 0.56 | 4.08E-02 | Not documented gene |
| *BBS12* | 0.56 | 4.10E-02 | Not documented gene |
| *DSTN* | 0.56 | 4.10E-02 | Not documented gene |
| *CCL13* | 0.56 | 4.10E-02 | Not documented gene |
| *TUBD1* | 0.56 | 4.11E-02 | Not documented gene |
| *IFNGR1* | 0.56 | 4.11E-02 | Not documented gene |
| *ZNF502* | 0.56 | 4.11E-02 | Not documented gene |
| *MPO* | 0.56 | 4.11E-02 | Not documented gene |
| *HIVEP3* | 0.56 | 4.12E-02 | Not documented gene |
| *DST* | 0.55 | 4.12E-02 | Not documented gene |
| *CENPM* | 0.55 | 4.13E-02 | Not documented gene |
| *ACOT9* | 0.55 | 4.13E-02 | Not documented gene |
| *HIST1H2AE* | 0.55 | 4.14E-02 | Not documented gene |
| *SAP130* | 0.55 | 4.15E-02 | Not documented gene |
| *TMC3* | 0.55 | 4.16E-02 | Not documented gene |
| *DKFZP434B0335* | 0.55 | 4.16E-02 | Not documented gene |
| *CCND2* | 0.55 | 4.17E-02 | Not documented gene |
| *DHCR7* | 0.54 | 4.18E-02 | Not documented gene |
| *CCDC3* | 0.54 | 4.18E-02 | Not documented gene |
| *SENP6* | 0.54 | 4.19E-02 | Not documented gene |
| *KCNK1* | 0.54 | 4.19E-02 | Not documented gene |
| *HLA-B* | 0.54 | 4.20E-02 | Documented gene |
| *GNG5* | 0.54 | 4.20E-02 | Not documented gene |
| *CPT1A* | 0.54 | 4.21E-02 | Not documented gene |
| *ITGBL1* | 0.54 | 4.21E-02 | Not documented gene |
| *SLC25A16* | 0.54 | 4.21E-02 | Not documented gene |
| *HOXD4* | 0.54 | 4.22E-02 | Not documented gene |
| *GPM6A* | 0.54 | 4.22E-02 | Not documented gene |
| *SNORD31* | 0.54 | 4.22E-02 | Not documented gene |
| *RND2* | 0.54 | 4.23E-02 | Not documented gene |
| *PCYT1A* | 0.54 | 4.23E-02 | Not documented gene |
| *NDFIP2* | 0.54 | 4.24E-02 | Not documented gene |
| *TIGD6* | 0.54 | 4.24E-02 | Not documented gene |
| *DDX54* | 0.53 | 4.25E-02 | Not documented gene |
| *GBP2* | 0.53 | 4.25E-02 | Not documented gene |
| *TFAP2E* | 0.53 | 4.26E-02 | Not documented gene |
| *PNO1* | 0.53 | 4.26E-02 | Not documented gene |
| *STRC* | 0.53 | 4.27E-02 | Not documented gene |
| *IQUB* | 0.53 | 4.29E-02 | Not documented gene |
| *ZNF428* | 0.53 | 4.29E-02 | Not documented gene |
| *HBD* | 0.53 | 4.29E-02 | Not documented gene |
| *CENPC1* | 0.53 | 4.30E-02 | Not documented gene |
| *MTMR4* | 0.53 | 4.31E-02 | Not documented gene |
| *SERPINA4* | 0.52 | 4.32E-02 | Not documented gene |
| *NTSR1* | 0.52 | 4.33E-02 | Not documented gene |
| *PPT2* | 0.52 | 4.33E-02 | Not documented gene |
| *MGAT5* | 0.52 | 4.35E-02 | Documented gene |
| *CCDC59* | 0.52 | 4.35E-02 | Not documented gene |
| *FIGLA* | 0.52 | 4.36E-02 | Not documented gene |
| *SYTL3* | 0.52 | 4.36E-02 | Not documented gene |
| *ASGR2* | 0.52 | 4.36E-02 | Not documented gene |
| *ZNF205* | 0.52 | 4.37E-02 | Not documented gene |
| *ATP6V0D2* | 0.51 | 4.38E-02 | Not documented gene |
| *RAD50* | 0.51 | 4.39E-02 | Documented gene |
| *DHX57* | 0.51 | 4.39E-02 | Not documented gene |
| *SFRS4* | 0.51 | 4.39E-02 | Not documented gene |
| *SNF1LK2* | 0.51 | 4.42E-02 | Not documented gene |
| *ZNF331* | 0.51 | 4.42E-02 | Not documented gene |
| *ORM2* | 0.51 | 4.44E-02 | Not documented gene |
| *CAPS* | 0.51 | 4.44E-02 | Not documented gene |
| *LRRC8E* | 0.50 | 4.45E-02 | Not documented gene |
| *2-Sep* | 0.50 | 4.45E-02 | Not documented gene |
| *SNED1* | 0.50 | 4.46E-02 | Not documented gene |
| *OSGEP* | 0.50 | 4.47E-02 | Not documented gene |
| *FSD1* | 0.50 | 4.48E-02 | Not documented gene |
| *YTHDC1* | 0.50 | 4.48E-02 | Not documented gene |
| *UQCRC2* | 0.50 | 4.49E-02 | Not documented gene |
| *DHTKD1* | 0.50 | 4.50E-02 | Not documented gene |
| *PEX1* | 0.50 | 4.50E-02 | Not documented gene |
| *EPHA2* | 0.50 | 4.50E-02 | Not documented gene |
| *CCR2* | 0.49 | 4.51E-02 | Not documented gene |
| *CSTF1* | 0.49 | 4.53E-02 | Not documented gene |
| *SIGLEC9* | 0.49 | 4.53E-02 | Not documented gene |
| *ZNF484* | 0.49 | 4.54E-02 | Not documented gene |
| *ADHFE1* | 0.49 | 4.54E-02 | Not documented gene |
| *EPS8* | 0.49 | 4.54E-02 | Not documented gene |
| *F2RL1* | 0.49 | 4.54E-02 | Not documented gene |
| *TNIP3* | 0.49 | 4.54E-02 | Not documented gene |
| *ZCWPW2* | 0.49 | 4.56E-02 | Not documented gene |
| *ERAP1* | 0.49 | 4.57E-02 | Not documented gene |
| *VRK3* | 0.49 | 4.57E-02 | Not documented gene |
| *MAP2K2* | 0.49 | 4.57E-02 | Not documented gene |
| *PFAAP5* | 0.48 | 4.57E-02 | Not documented gene |
| *DHRS7* | 0.48 | 4.57E-02 | Not documented gene |
| *ETFDH* | 0.48 | 4.59E-02 | Not documented gene |
| *PIK3R2* | 0.48 | 4.59E-02 | Not documented gene |
| *BRF2* | 0.48 | 4.59E-02 | Not documented gene |
| *ADAM19* | 0.48 | 4.59E-02 | Not documented gene |
| *ORM1* | 0.48 | 4.60E-02 | Not documented gene |
| *SPRR1A* | 0.48 | 4.61E-02 | Not documented gene |
| *SLC35D2* | 0.48 | 4.62E-02 | Not documented gene |
| *ZSCAN18* | 0.48 | 4.62E-02 | Not documented gene |
| *COX5A* | 0.48 | 4.62E-02 | Not documented gene |
| *TMEM209* | 0.48 | 4.63E-02 | Not documented gene |
| *ZNF358* | 0.48 | 4.63E-02 | Not documented gene |
| *DKFZP686E2433* | 0.48 | 4.63E-02 | Not documented gene |
| *TTC21A* | 0.48 | 4.63E-02 | Not documented gene |
| *ERICH1* | 0.47 | 4.66E-02 | Not documented gene |
| *SNF8* | 0.47 | 4.66E-02 | Not documented gene |
| *ACTR1B* | 0.47 | 4.66E-02 | Not documented gene |
| *MAP2K1* | 0.47 | 4.67E-02 | Not documented gene |
| *RAB35* | 0.47 | 4.69E-02 | Not documented gene |
| *MAX* | 0.47 | 4.69E-02 | Not documented gene |
| *EHD2* | 0.47 | 4.70E-02 | Not documented gene |
| *PPP1CA* | 0.47 | 4.72E-02 | Not documented gene |
| *ZFP42* | 0.47 | 4.72E-02 | Not documented gene |
| *XBP1* | 0.46 | 4.72E-02 | Not documented gene |
| *ZNF467* | 0.46 | 4.73E-02 | Not documented gene |
| *ZNF438* | 0.46 | 4.73E-02 | Not documented gene |
| *PLEK2* | 0.46 | 4.73E-02 | Not documented gene |
| *MIA3* | 0.46 | 4.75E-02 | Not documented gene |
| *MAP4K1* | 0.46 | 4.75E-02 | Not documented gene |
| *GPR109B* | 0.46 | 4.75E-02 | Not documented gene |
| *SUMO4* | 0.46 | 4.75E-02 | Not documented gene |
| *PHKB* | 0.46 | 4.76E-02 | Not documented gene |
| *UGCGL2* | 0.46 | 4.76E-02 | Not documented gene |
| *PROS1* | 0.46 | 4.76E-02 | Not documented gene |
| *RNF175* | 0.46 | 4.77E-02 | Not documented gene |
| *EEF1E1* | 0.46 | 4.77E-02 | Not documented gene |
| *FANCM* | 0.46 | 4.78E-02 | Not documented gene |
| *COPB1* | 0.46 | 4.78E-02 | Not documented gene |
| *ZNF418* | 0.46 | 4.78E-02 | Not documented gene |
| *PRKAB2* | 0.46 | 4.79E-02 | Not documented gene |
| *OSBPL10* | 0.45 | 4.79E-02 | Not documented gene |
| *PTPN11* | 0.45 | 4.80E-02 | Not documented gene |
| *ATF6* | 0.45 | 4.81E-02 | Not documented gene |
| *ZNF652* | 0.45 | 4.81E-02 | Documented gene |
| *SLC25A31* | 0.45 | 4.82E-02 | Not documented gene |
| *IL11RA* | 0.45 | 4.83E-02 | Not documented gene |
| *SLC2A11* | 0.45 | 4.83E-02 | Not documented gene |
| *PARP11* | 0.45 | 4.84E-02 | Not documented gene |
| *PRR15* | 0.45 | 4.85E-02 | Not documented gene |
| *CLEC11A* | 0.44 | 4.88E-02 | Not documented gene |
| *SYNJ2BP* | 0.44 | 4.88E-02 | Not documented gene |
| *SETX* | 0.44 | 4.89E-02 | Not documented gene |
| *PELI1* | 0.44 | 4.89E-02 | Not documented gene |
| *ZNF197* | 0.44 | 4.90E-02 | Not documented gene |
| *SFRS11* | 0.44 | 4.92E-02 | Not documented gene |
| *ORC3L* | 0.44 | 4.92E-02 | Not documented gene |
| *RSBN1* | 0.44 | 4.92E-02 | Not documented gene |
| *FAM108C1* | 0.44 | 4.93E-02 | Not documented gene |
| *DLX3* | 0.44 | 4.93E-02 | Not documented gene |
| *MUC16* | 0.44 | 4.93E-02 | Documented gene |
| *KRT36* | 0.43 | 4.95E-02 | Not documented gene |
| *ETFB* | 0.43 | 4.96E-02 | Not documented gene |
| *BEST2* | 0.43 | 4.96E-02 | Not documented gene |
| *POU2F3* | 0.43 | 4.97E-02 | Not documented gene |
| *TSN* | 0.43 | 4.98E-02 | Not documented gene |
| *DNMT1* | 0.43 | 4.98E-02 | Not documented gene |
| *AGPAT3* | 0.43 | 4.99E-02 | Not documented gene |

**Supplemental Table S2. 228 Sherlock-identified genes from discovery dataset #3 overlapped with MAGMA-identified genes**

| **Gene Name** | **LBF** | **Sherlock-based P-value** | **MAGMA-based P-value** | **GWAS Catalog** |
| --- | --- | --- | --- | --- |
| *HLA-DRB5* | 10.67 | 7.93E-07 | 7.57E-10 | Documented gene |
| *HLA-DRB1* | 8.87 | 7.93E-07 | 1.41E-11 | Documented gene |
| *HLA-DOB* | 7.86 | 1.59E-06 | 2.43E-06 | Not documented gene |
| *IKZF3* | 7.17 | 1.59E-06 | 6.63E-05 | Documented gene |
| *IL18R1* | 7.17 | 1.59E-06 | 4.99E-17 | Documented gene |
| *DEXI* | 7.10 | 1.59E-06 | 9.70E-15 | Not documented gene |
| *SUOX* | 7.08 | 3.17E-06 | 2.13E-07 | Documented gene |
| *RERE* | 6.89 | 4.76E-06 | 4.36E-08 | Documented gene |
| *GNGT2* | 6.34 | 1.11E-05 | 1.42E-02 | Not documented gene |
| *SLC22A4* | 5.99 | 1.90E-05 | 5.40E-05 | Not documented gene |
| *HLA-DQA1* | 5.74 | 2.85E-05 | 2.90E-23 | Documented gene |
| *NDFIP1* | 5.57 | 3.80E-05 | 9.42E-06 | Documented gene |
| *IER3* | 5.45 | 4.76E-05 | 1.40E-02 | Not documented gene |
| *D2HGDH* | 5.43 | 5.07E-05 | 3.29E-13 | Documented gene |
| *ZNF76* | 4.82 | 1.17E-04 | 1.38E-04 | Not documented gene |
| *RPS26* | 4.74 | 1.27E-04 | 2.90E-05 | Documented gene |
| *FES* | 4.66 | 1.43E-04 | 5.96E-05 | Not documented gene |
| *STAT2* | 4.53 | 1.76E-04 | 1.89E-03 | Not documented gene |
| *SLC22A5* | 4.49 | 1.85E-04 | 1.23E-06 | Documented gene |
| *GPBAR1* | 4.47 | 1.85E-04 | 1.30E-04 | Not documented gene |
| *PNKD* | 4.44 | 1.92E-04 | 1.53E-04 | Not documented gene |
| *CCBL1* | 4.44 | 1.92E-04 | 1.10E-02 | Not documented gene |
| *TOMM40L* | 4.43 | 1.95E-04 | 1.17E-04 | Not documented gene |
| *MGAT4A* | 4.39 | 2.06E-04 | 3.60E-05 | Not documented gene |
| *STAT6* | 4.35 | 2.19E-04 | 1.94E-11 | Documented gene |
| *ENDOG* | 4.29 | 2.39E-04 | 4.47E-04 | Not documented gene |
| *CDC42SE2* | 4.09 | 3.61E-04 | 1.48E-02 | Not documented gene |
| *RTN1* | 3.91 | 4.88E-04 | 8.26E-04 | Not documented gene |
| *FCER1G* | 3.64 | 7.29E-04 | 4.44E-06 | Documented gene |
| *DEF6* | 3.61 | 7.53E-04 | 3.38E-03 | Not documented gene |
| *SEPHS1* | 3.46 | 9.04E-04 | 1.79E-02 | Not documented gene |
| *ULK3* | 3.44 | 9.23E-04 | 6.79E-04 | Not documented gene |
| *TCN2* | 3.44 | 9.24E-04 | 4.16E-02 | Not documented gene |
| *DUSP18* | 3.36 | 1.02E-03 | 4.34E-04 | Not documented gene |
| *USP35* | 3.34 | 1.06E-03 | 1.78E-02 | Not documented gene |
| *FADS2* | 3.34 | 1.06E-03 | 3.63E-04 | Documented gene |
| *SDF4* | 3.14 | 1.36E-03 | 3.76E-02 | Not documented gene |
| *SACM1L* | 3.13 | 1.39E-03 | 4.67E-02 | Not documented gene |
| *NSMCE1* | 3.11 | 1.43E-03 | 2.03E-02 | Not documented gene |
| *RPS27L* | 3.03 | 1.60E-03 | 2.63E-02 | Not documented gene |
| *CEP192* | 3.01 | 1.64E-03 | 5.53E-05 | Not documented gene |
| *TSGA10* | 2.95 | 1.76E-03 | 3.71E-03 | Not documented gene |
| *MPI* | 2.89 | 1.90E-03 | 1.12E-05 | Not documented gene |
| *KIF16B* | 2.86 | 1.98E-03 | 4.03E-02 | Documented gene |
| *DAGLA* | 2.86 | 1.98E-03 | 1.34E-02 | Not documented gene |
| *SCAMP2* | 2.80 | 2.14E-03 | 3.46E-05 | Not documented gene |
| *GLB1* | 2.74 | 2.32E-03 | 2.98E-02 | Documented gene |
| *FEZ2* | 2.72 | 2.39E-03 | 1.13E-04 | Not documented gene |
| *PDIA6* | 2.71 | 2.41E-03 | 9.66E-03 | Not documented gene |
| *PIK3R3* | 2.69 | 2.46E-03 | 1.82E-02 | Not documented gene |
| *RUNX3* | 2.53 | 3.06E-03 | 2.41E-06 | Documented gene |
| *CDK9* | 2.52 | 3.10E-03 | 9.28E-03 | Not documented gene |
| *CTSK* | 2.51 | 3.18E-03 | 4.85E-04 | Not documented gene |
| *MINK1* | 2.49 | 3.24E-03 | 1.58E-02 | Not documented gene |
| *STARD3* | 2.44 | 3.47E-03 | 7.48E-07 | Not documented gene |
| *ZNF23* | 2.44 | 3.48E-03 | 3.81E-02 | Not documented gene |
| *IL23A* | 2.44 | 3.48E-03 | 1.42E-04 | Not documented gene |
| *VPS4A* | 2.43 | 3.50E-03 | 8.83E-03 | Not documented gene |
| *MFSD5* | 2.43 | 3.51E-03 | 1.45E-02 | Not documented gene |
| *TLR6* | 2.40 | 3.63E-03 | 4.10E-02 | Documented gene |
| *DEFB1* | 2.36 | 3.80E-03 | 2.67E-02 | Not documented gene |
| *CDH23* | 2.35 | 3.87E-03 | 1.15E-02 | Not documented gene |
| *ST3GAL5* | 2.31 | 4.11E-03 | 8.26E-03 | Not documented gene |
| *ALMS1* | 2.29 | 4.18E-03 | 1.30E-02 | Not documented gene |
| *MRPL21* | 2.27 | 4.29E-03 | 1.36E-03 | Not documented gene |
| *IL27RA* | 2.25 | 4.39E-03 | 3.55E-02 | Not documented gene |
| *FAM20B* | 2.23 | 4.51E-03 | 4.84E-03 | Not documented gene |
| *ZC3H10* | 2.23 | 4.53E-03 | 2.68E-04 | Not documented gene |
| *HSPA1A* | 2.21 | 4.63E-03 | 1.77E-05 | Not documented gene |
| *EPC1* | 2.14 | 5.03E-03 | 1.40E-02 | Not documented gene |
| *NCF1* | 2.14 | 5.03E-03 | 3.43E-02 | Not documented gene |
| *TAP1* | 2.14 | 5.07E-03 | 2.76E-04 | Not documented gene |
| *HIATL1* | 2.11 | 5.22E-03 | 3.26E-04 | Not documented gene |
| *CTSS* | 2.10 | 5.30E-03 | 1.16E-02 | Not documented gene |
| *MAPKAPK3* | 2.08 | 5.50E-03 | 4.84E-03 | Not documented gene |
| *CORO1C* | 2.06 | 5.58E-03 | 3.76E-02 | Not documented gene |
| *ZNF74* | 2.06 | 5.64E-03 | 3.76E-02 | Not documented gene |
| *PCMT1* | 2.01 | 5.98E-03 | 1.02E-02 | Not documented gene |
| *NUP43* | 1.99 | 6.11E-03 | 1.47E-02 | Not documented gene |
| *PHF5A* | 1.97 | 6.26E-03 | 9.46E-03 | Documented gene |
| *ACVR1* | 1.96 | 6.42E-03 | 2.98E-03 | Not documented gene |
| *IL4R* | 1.93 | 6.69E-03 | 6.53E-06 | Documented gene |
| *KCNQ1* | 1.90 | 6.96E-03 | 8.64E-04 | Not documented gene |
| *SNAP29* | 1.88 | 7.06E-03 | 2.07E-03 | Not documented gene |
| *NIP7* | 1.88 | 7.15E-03 | 1.66E-02 | Not documented gene |
| *COPA* | 1.87 | 7.21E-03 | 3.60E-04 | Not documented gene |
| *CDKL1* | 1.87 | 7.25E-03 | 4.36E-03 | Not documented gene |
| *TLK1* | 1.86 | 7.34E-03 | 7.10E-03 | Not documented gene |
| *HSF2* | 1.83 | 7.65E-03 | 4.08E-02 | Not documented gene |
| *ZNF2* | 1.83 | 7.70E-03 | 1.13E-02 | Not documented gene |
| *SELL* | 1.80 | 7.99E-03 | 3.37E-04 | Not documented gene |
| *HP1BP3* | 1.80 | 8.00E-03 | 3.39E-02 | Not documented gene |
| *LILRB2* | 1.78 | 8.15E-03 | 1.05E-02 | Not documented gene |
| *MYO7A* | 1.77 | 8.31E-03 | 3.80E-02 | Not documented gene |
| *PELO* | 1.77 | 8.31E-03 | 8.41E-03 | Not documented gene |
| *TRAPPC6B* | 1.74 | 8.59E-03 | 4.56E-02 | Not documented gene |
| *GEN1* | 1.73 | 8.65E-03 | 1.91E-02 | Not documented gene |
| *FADD* | 1.72 | 8.76E-03 | 1.43E-04 | Not documented gene |
| *TMTC4* | 1.71 | 8.91E-03 | 3.78E-03 | Not documented gene |
| *TIMP2* | 1.69 | 9.16E-03 | 4.19E-03 | Not documented gene |
| *UNC50* | 1.68 | 9.23E-03 | 1.05E-02 | Not documented gene |
| *PEX19* | 1.66 | 9.43E-03 | 4.47E-03 | Not documented gene |
| *GRAMD4* | 1.65 | 9.54E-03 | 4.78E-02 | Not documented gene |
| *ICAM5* | 1.62 | 9.86E-03 | 4.93E-03 | Not documented gene |
| *SPTBN1* | 1.62 | 9.87E-03 | 1.40E-02 | Not documented gene |
| *LYPLAL1* | 1.62 | 9.91E-03 | 5.84E-04 | Not documented gene |
| *PPOX* | 1.60 | 1.02E-02 | 3.17E-05 | Not documented gene |
| *IGHMBP2* | 1.59 | 1.03E-02 | 6.03E-04 | Not documented gene |
| *GPR18* | 1.59 | 1.03E-02 | 2.86E-08 | Not documented gene |
| *SMAD7* | 1.56 | 1.06E-02 | 3.86E-03 | Not documented gene |
| *ICAM4* | 1.53 | 1.10E-02 | 2.01E-03 | Not documented gene |
| *IRF1* | 1.53 | 1.10E-02 | 6.45E-08 | Not documented gene |
| *TOM1* | 1.52 | 1.11E-02 | 2.26E-02 | Not documented gene |
| *DAP* | 1.49 | 1.16E-02 | 6.34E-04 | Not documented gene |
| *SMARCE1* | 1.49 | 1.16E-02 | 7.47E-04 | Documented gene |
| *LRRC20* | 1.48 | 1.18E-02 | 3.32E-03 | Not documented gene |
| *ZNF514* | 1.47 | 1.20E-02 | 1.20E-02 | Not documented gene |
| *PAK4* | 1.40 | 1.30E-02 | 3.04E-03 | Not documented gene |
| *PPP1R12A* | 1.39 | 1.32E-02 | 1.26E-02 | Not documented gene |
| *STEAP4* | 1.39 | 1.32E-02 | 1.43E-03 | Not documented gene |
| *UBA7* | 1.38 | 1.33E-02 | 1.18E-02 | Not documented gene |
| *TPRKB* | 1.38 | 1.34E-02 | 2.65E-03 | Not documented gene |
| *GFRA2* | 1.35 | 1.38E-02 | 1.00E-02 | Documented gene |
| *ARRDC4* | 1.32 | 1.44E-02 | 3.57E-02 | Not documented gene |
| *COG6* | 1.30 | 1.46E-02 | 3.51E-03 | Documented gene |
| *MICA* | 1.29 | 1.49E-02 | 1.57E-05 | Not documented gene |
| *KATNA1* | 1.29 | 1.50E-02 | 2.11E-02 | Not documented gene |
| *SEMA3F* | 1.27 | 1.53E-02 | 3.47E-03 | Not documented gene |
| *DECR2* | 1.23 | 1.62E-02 | 2.22E-02 | Not documented gene |
| *TAF7* | 1.20 | 1.69E-02 | 1.14E-02 | Not documented gene |
| *HECA* | 1.19 | 1.70E-02 | 4.27E-02 | Not documented gene |
| *EIF4E3* | 1.18 | 1.72E-02 | 2.11E-03 | Not documented gene |
| *SSR3* | 1.18 | 1.73E-02 | 4.63E-02 | Documented gene |
| *FAM136A* | 1.17 | 1.76E-02 | 2.47E-02 | Not documented gene |
| *TOM1L2* | 1.16 | 1.78E-02 | 4.05E-02 | Not documented gene |
| *SLC15A2* | 1.13 | 1.83E-02 | 4.05E-02 | Not documented gene |
| *IFRD1* | 1.13 | 1.85E-02 | 2.18E-02 | Not documented gene |
| *CIITA* | 1.11 | 1.89E-02 | 1.67E-09 | Not documented gene |
| *CCS* | 1.10 | 1.91E-02 | 3.16E-04 | Not documented gene |
| *HARS2* | 1.08 | 1.97E-02 | 9.82E-03 | Not documented gene |
| *HOXB6* | 1.08 | 1.97E-02 | 9.61E-03 | Not documented gene |
| *HNRNPL* | 1.07 | 1.99E-02 | 2.02E-02 | Not documented gene |
| *ATP5S* | 1.04 | 2.08E-02 | 7.33E-04 | Not documented gene |
| *ANP32B* | 1.03 | 2.09E-02 | 2.41E-03 | Not documented gene |
| *EIF2S2* | 1.02 | 2.12E-02 | 9.10E-03 | Not documented gene |
| *WDR55* | 1.02 | 2.14E-02 | 1.35E-02 | Not documented gene |
| *PDF* | 0.97 | 2.28E-02 | 1.43E-02 | Not documented gene |
| *LNPEP* | 0.97 | 2.28E-02 | 3.98E-02 | Not documented gene |
| *IFT57* | 0.96 | 2.32E-02 | 2.24E-02 | Not documented gene |
| *HIST2H4A* | 0.96 | 2.32E-02 | 8.98E-03 | Not documented gene |
| *RUVBL1* | 0.96 | 2.32E-02 | 6.79E-04 | Not documented gene |
| *PTCD1* | 0.95 | 2.33E-02 | 4.80E-02 | Not documented gene |
| *NOL6* | 0.94 | 2.37E-02 | 1.13E-02 | Not documented gene |
| *SLC35B4* | 0.94 | 2.38E-02 | 1.34E-02 | Not documented gene |
| *RRS1* | 0.92 | 2.43E-02 | 1.48E-02 | Not documented gene |
| *TTC19* | 0.90 | 2.52E-02 | 3.23E-02 | Not documented gene |
| *ECH1* | 0.90 | 2.53E-02 | 2.28E-02 | Not documented gene |
| *CEP57* | 0.85 | 2.67E-02 | 2.05E-03 | Not documented gene |
| *FPR1* | 0.85 | 2.69E-02 | 8.88E-03 | Not documented gene |
| *SIRT1* | 0.83 | 2.74E-02 | 3.50E-02 | Not documented gene |
| *PLCD4* | 0.82 | 2.80E-02 | 1.72E-02 | Not documented gene |
| *RNF167* | 0.81 | 2.83E-02 | 3.74E-02 | Not documented gene |
| *ZNF213* | 0.80 | 2.90E-02 | 2.48E-02 | Not documented gene |
| *APBB3* | 0.77 | 2.99E-02 | 3.39E-03 | Not documented gene |
| *TRIM37* | 0.77 | 3.00E-02 | 4.40E-02 | Not documented gene |
| *BAZ2B* | 0.77 | 3.01E-02 | 3.25E-02 | Not documented gene |
| *RET* | 0.77 | 3.03E-02 | 8.44E-03 | Not documented gene |
| *GALT* | 0.75 | 3.07E-02 | 2.35E-02 | Not documented gene |
| *ABCC9* | 0.75 | 3.08E-02 | 1.82E-03 | Not documented gene |
| *SRA1* | 0.75 | 3.08E-02 | 4.50E-03 | Not documented gene |
| *MSI2* | 0.75 | 3.08E-02 | 4.89E-02 | Not documented gene |
| *CHI3L2* | 0.75 | 3.10E-02 | 4.69E-03 | Not documented gene |
| *TERF2* | 0.75 | 3.11E-02 | 4.80E-03 | Not documented gene |
| *TMEM175* | 0.74 | 3.13E-02 | 4.77E-02 | Not documented gene |
| *RIN1* | 0.74 | 3.14E-02 | 1.79E-04 | Not documented gene |
| *CCDC82* | 0.73 | 3.19E-02 | 1.62E-03 | Not documented gene |
| *UBE3B* | 0.73 | 3.20E-02 | 1.70E-02 | Not documented gene |
| *GRAP2* | 0.71 | 3.25E-02 | 3.38E-03 | Not documented gene |
| *GAB2* | 0.71 | 3.25E-02 | 2.12E-02 | Not documented gene |
| *YIF1A* | 0.70 | 3.29E-02 | 6.02E-04 | Not documented gene |
| *MGMT* | 0.70 | 3.31E-02 | 3.82E-02 | Not documented gene |
| *GCM1* | 0.70 | 3.34E-02 | 6.34E-03 | Not documented gene |
| *PHOSPHO1* | 0.70 | 3.34E-02 | 1.82E-08 | Not documented gene |
| *PARP10* | 0.69 | 3.38E-02 | 1.85E-02 | Not documented gene |
| *GNL1* | 0.68 | 3.41E-02 | 3.28E-02 | Not documented gene |
| *ERBB3* | 0.67 | 3.45E-02 | 2.37E-04 | Documented gene |
| *PHYH* | 0.66 | 3.49E-02 | 1.92E-02 | Not documented gene |
| *SNTB2* | 0.66 | 3.52E-02 | 9.71E-03 | Not documented gene |
| *XRRA1* | 0.66 | 3.53E-02 | 3.92E-02 | Not documented gene |
| *CSNK2B* | 0.66 | 3.55E-02 | 2.16E-02 | Not documented gene |
| *LEKR1* | 0.65 | 3.57E-02 | 1.26E-02 | Not documented gene |
| *DOCK10* | 0.65 | 3.59E-02 | 1.47E-02 | Not documented gene |
| *PRR16* | 0.65 | 3.59E-02 | 3.63E-05 | Not documented gene |
| *CDC42BPB* | 0.64 | 3.62E-02 | 2.18E-02 | Not documented gene |
| *CDC25B* | 0.64 | 3.62E-02 | 3.33E-02 | Not documented gene |
| *PSMB9* | 0.64 | 3.63E-02 | 5.98E-03 | Not documented gene |
| *LTA* | 0.63 | 3.66E-02 | 4.61E-05 | Not documented gene |
| *SCAND1* | 0.63 | 3.66E-02 | 1.29E-02 | Not documented gene |
| *GHITM* | 0.63 | 3.68E-02 | 2.17E-02 | Not documented gene |
| *HEXDC* | 0.61 | 3.79E-02 | 1.99E-02 | Not documented gene |
| *RPH3A* | 0.61 | 3.80E-02 | 4.34E-03 | Not documented gene |
| *FCGR1B* | 0.60 | 3.84E-02 | 1.46E-02 | Not documented gene |
| *IPO8* | 0.59 | 3.90E-02 | 1.52E-02 | Not documented gene |
| *HLA-C* | 0.59 | 3.92E-02 | 4.03E-02 | Not documented gene |
| *CHD1L* | 0.59 | 3.94E-02 | 1.65E-03 | Not documented gene |
| *CHMP2B* | 0.57 | 4.02E-02 | 4.69E-02 | Not documented gene |
| *BBS12* | 0.56 | 4.10E-02 | 4.88E-02 | Not documented gene |
| *SENP6* | 0.54 | 4.19E-02 | 3.56E-02 | Not documented gene |
| *HLA-B* | 0.54 | 4.20E-02 | 2.80E-05 | Documented gene |
| *CPT1A* | 0.54 | 4.21E-02 | 1.64E-02 | Not documented gene |
| *TIGD6* | 0.54 | 4.24E-02 | 2.38E-03 | Not documented gene |
| *MTMR4* | 0.53 | 4.31E-02 | 3.65E-02 | Not documented gene |
| *PPT2* | 0.52 | 4.33E-02 | 4.17E-06 | Not documented gene |
| *SYTL3* | 0.52 | 4.36E-02 | 7.11E-04 | Not documented gene |
| *RAD50* | 0.51 | 4.39E-02 | 2.80E-06 | Documented gene |
| *DHX57* | 0.51 | 4.39E-02 | 1.37E-02 | Not documented gene |
| *ORM2* | 0.51 | 4.44E-02 | 7.12E-03 | Not documented gene |
| *FSD1* | 0.50 | 4.48E-02 | 4.25E-02 | Not documented gene |
| *F2RL1* | 0.49 | 4.54E-02 | 6.05E-03 | Not documented gene |
| *ORM1* | 0.48 | 4.60E-02 | 9.01E-03 | Not documented gene |
| *COX5A* | 0.48 | 4.62E-02 | 3.21E-06 | Not documented gene |
| *ZNF358* | 0.48 | 4.63E-02 | 3.77E-02 | Not documented gene |
| *ZNF438* | 0.46 | 4.73E-02 | 2.94E-02 | Not documented gene |
| *PROS1* | 0.46 | 4.76E-02 | 7.46E-03 | Not documented gene |
| *ZNF652* | 0.45 | 4.81E-02 | 2.76E-08 | Documented gene |
| *IL11RA* | 0.45 | 4.83E-02 | 3.12E-02 | Not documented gene |
| *CLEC11A* | 0.44 | 4.88E-02 | 3.27E-02 | Not documented gene |
| *POU2F3* | 0.43 | 4.97E-02 | 9.71E-03 | Not documented gene |

**Supplemental Table S3. Significant KEGG pathways enriched by severe asthma-relevant genes identified from Sherlock Bayesian analysis**

| **Pathway ID** | **Pathway Terms** | **P value** | **Corrected P value** | **Associated gene proportion** | **Associated genes** |
| --- | --- | --- | --- | --- | --- |
| KEGG:04612 | Antigen processing and presentation | 1.48E-07 | 4.30E-06 | 0.13 | *CIITA, CTSS, HLA-B, HLA-C, HLA-DOB, HLA-DQA1, HLA-DRB1, HLA-DRB5, HSPA1A, TAP1* |
| KEGG:04940 | Type I diabetes mellitus | 2.53E-06 | 7.09E-05 | 0.16 | *HLA-B, HLA-C, HLA-DOB, HLA-DQA1, HLA-DRB1, HLA-DRB5, LTA* |
| KEGG:05321 | Inflammatory bowel disease (IBD) | 4.21E-06 | 1.14E-04 | 0.12 | *HLA-DOB, HLA-DQA1, HLA-DRB1, HLA-DRB5, IL18R1, IL23A, IL4R, STAT6* |
| KEGG:05168 | Herpes simplex infection | 1.53E-05 | 3.97E-04 | 0.06 | *CSNK2B, FADD, HLA-B, HLA-C, HLA-DOB, HLA-DQA1, HLA-DRB1, HLA-DRB5, LTA, POU2F3, STAT2, TAP1* |
| KEGG:05330 | Allograft rejection | 1.65E-05 | 4.12E-04 | 0.16 | *HLA-B, HLA-C, HLA-DOB, HLA-DQA1, HLA-DRB1, HLA-DRB5* |
| KEGG:05332 | Graft-versus-host disease | 2.59E-05 | 6.21E-04 | 0.15 | *HLA-B, HLA-C, HLA-DOB, HLA-DQA1, HLA-DRB1, HLA-DRB5* |
| KEGG:04145 | Phagosome | 7.24E-05 | 1.67E-03 | 0.07 | *CTSS, HLA-B, HLA-C, HLA-DOB, HLA-DQA1, HLA-DRB1, HLA-DRB5, NCF1, TAP1, TLR6* |
| KEGG:05310 | Asthma | 7.82E-05 | 1.72E-03 | 0.16 | *FCER1G, HLA-DOB, HLA-DQA1, HLA-DRB1, HLA-DRB5* |
| KEGG:05320 | Autoimmune thyroid disease | 1.15E-04 | 2.41E-03 | 0.11 | *HLA-B, HLA-C, HLA-DOB, HLA-DQA1, HLA-DRB1, HLA-DRB5* |
| KEGG:04659 | Th17 cell differentiation | 1.63E-04 | 3.27E-03 | 0.07 | *HLA-DOB, HLA-DQA1, HLA-DRB1, HLA-DRB5, IL23A, IL27RA, IL4R, STAT6* |
| KEGG:05416 | Viral myocarditis | 2.09E-04 | 3.98E-03 | 0.10 | *HLA-B, HLA-C, HLA-DOB, HLA-DQA1, HLA-DRB1, HLA-DRB5* |
| KEGG:05152 | Tuberculosis | 2.80E-04 | 5.04E-03 | 0.06 | *CIITA, CTSS, FADD, FCER1G, HLA-DOB, HLA-DQA1, HLA-DRB1, HLA-DRB5, IL23A, TLR6* |
| KEGG:04658 | Th1 and Th2 cell differentiation | 3.82E-04 | 6.49E-03 | 0.08 | *HLA-DOB, HLA-DQA1, HLA-DRB1, HLA-DRB5, IL4R, RUNX3, STAT6* |
| KEGG:05150 | Staphylococcus aureus infection | 1.31E-03 | 2.10E-02 | 0.09 | *FPR1, HLA-DOB, HLA-DQA1, HLA-DRB1, HLA-DRB5* |
| KEGG:05323 | Rheumatoid arthritis | 2.00E-03 | 3.01E-02 | 0.07 | *CTSK, HLA-DOB, HLA-DQA1, HLA-DRB1, HLA-DRB5, IL23A* |
| KEGG:04640 | Hematopoietic cell lineage | 2.93E-03 | 4.10E-02 | 0.06 | *HLA-DOB, HLA-DQA1, HLA-DRB1, HLA-DRB5, IL11RA, IL4R* |
| KEGG:05164 | Influenza A | 3.81E-03 | 4.96E-02 | 0.05 | *CIITA, HLA-DOB, HLA-DQA1, HLA-DRB1, HLA-DRB5, HSPA1A, PIK3R3, STAT2* |

**Supplemental Table S4. Significant GO-terms of molecular function enriched by severe asthma-relevant genes identified from Sherlock Bayesian analysis**

| **GO-term ID** | **GO Terms** | **P value** | **Corrected P value** | **Associated genes proportion** | **Associated genes** |
| --- | --- | --- | --- | --- | --- |
| GO:0042605 | Peptide antigen binding | 7.38E-06 | 5.17E-05 | 0.16 | *HLA-B, HLA-C, HLA-DQA1, HLA-DRB1, HLA-DRB5, TAP1* |
| GO:0042277 | Peptide binding | 2.86E-04 | 1.72E-03 | 0.04 | *ACVR1, APBB3, HLA-B, HLA-C, HLA-DQA1, HLA-DRB1, HLA-DRB5, LILRB2, LNPEP, PEX19, TAP1, TLR6* |
| GO:0004003 | ATP-dependent DNA helicase activity | 1.43E-03 | 7.15E-03 | 0.10 | *CHD1L, IGHMBP2, RAD50, RUVBL1* |
| GO:0043325 | Phosphatidylinositol-3,4-bisphosphate binding | 4.15E-03 | 1.66E-02 | 0.12 | *GAB2, KIF16B, NCF1* |
| GO:1902936 | Phosphatidylinositol bisphosphate binding | 6.74E-03 | 2.02E-02 | 0.05 | *GAB2, KCNQ1, KIF16B, NCF1, RPH3A* |
| GO:1904680 | Peptide transmembrane transporter activity | 6.85E-03 | 1.37E-02 | 0.10 | *SLC15A2, TAP1, TOMM40L* |
| GO:0005164 | Tumor necrosis factor receptor binding | 8.87E-03 | 8.87E-03 | 0.09 | *FADD, LTA, TRIM37* |

**Supplemental Table S5. Significant GO-terms of cellular component enriched by severe asthma-relevant genes identified from Sherlock Bayesian analysis**

| **GO-term ID** | **GO Terms** | **P value** | **Corrected P value** | **Associated genes proportion** | **Associated genes** |
| --- | --- | --- | --- | --- | --- |
| GO:0042611 | MHC protein complex | 4.09E-08 | 4.91E-07 | 0.24 | *HLA-B, HLA-C, HLA-DOB, HLA-DQA1, HLA-DRB1, HLA-DRB5, PNKD* |
| GO:0098797 | Plasma Membrane Protein Complex | 6.18E-07 | 6.79E-06 | 0.06 | *ABCC9, CORO1C, FADD, FCER1G, GNGT2, HLA-B, HLA-C, HLA-DOB, HLA-DQA1, HLA-DRB1, HLA-DRB5, PNKD, RET, SMAD7, SNTB2* |
| GO:0042613 | MHC class II protein complex | 5.43E-05 | 5.43E-04 | 0.22 | *HLA-DOB, HLA-DQA1, HLA-DRB1, HLA-DRB5* |
| GO:0071556 | Integral Component Of Lumenal Side Of Endoplasmic Reticulum Membrane | 5.79E-05 | 5.21E-04 | 0.14 | *HLA-B, HLA-C, HLA-DQA1, HLA-DRB1, HLA-DRB5* |
| GO:0030176 | Integral Component Of Endoplasmic Reticulum Membrane | 1.20E-04 | 9.63E-04 | 0.06 | *HLA-B, HLA-C, HLA-DQA1, HLA-DRB1, HLA-DRB5, RTN1, SACM1L, SLC35B4, TAP1* |
| GO:0042612 | MHC class I protein complex | 2.63E-04 | 1.84E-03 | 0.27 | *HLA-B, HLA-C, PNKD* |
| GO:0030666 | Endocytic Vesicle Membrane | 1.58E-03 | 9.48E-03 | 0.04 | *FCGR1B, HLA-B, HLA-C, HLA-DQA1, HLA-DRB1, HLA-DRB5, TAP1, TLR6* |
| GO:0005798 | Golgi-Associated Vesicle | 3.85E-03 | 1.92E-02 | 0.04 | *COPA, HLA-B, HLA-C, HLA-DQA1, HLA-DRB1, HLA-DRB5, YIF1A* |
| GO:0030669 | Clathrin-Coated Endocytic Vesicle Membrane | 4.28E-03 | 1.71E-02 | 0.07 | *FCGR1B, HLA-DQA1, HLA-DRB1, HLA-DRB5* |
| GO:0035097 | Histone Methyltransferase Complex | 1.00E-02 | 3.00E-02 | 0.06 | *RUVBL1, SIRT1, TAF7, TRIM37* |
| GO:0045335 | Phagocytic Vesicle | 1.10E-02 | 2.19E-02 | 0.05 | *HLA-B, HLA-C, NCF1, TAP1, TLR6* |
| GO:0030173 | Integral Component Of Golgi Membrane | 1.68E-02 | 1.68E-02 | 0.07 | *SLC35B4, UNC50, YIF1A* |

**Supplemental Table S6. Significant GO-terms of biological process enriched by severe asthma-relevant genes identified from Sherlock Bayesian analysis**

| **GO-term ID** | **GO Terms** | **P value** | **Corrected P value** | **Associated genes proportion** | **Associated genes** |
| --- | --- | --- | --- | --- | --- |
| GO:0002703 | Regulation Of Leukocyte Mediated Immunity | 5.63E-08 | 4.22E-06 | 0.08 | *F2RL1, FADD, FCER1G, FES, GAB2, HLA-B, IL23A, IL27RA, IL4R, LTA, MICA, NDFIP1, SMAD7, STAT6* |
| GO:0048002 | Antigen Processing And Presentation Of Peptide Antigen | 2.13E-07 | 1.58E-05 | 0.07 | *CTSS, FCER1G, FCGR1B, HLA-B, HLA-C, HLA-DOB, HLA-DQA1, HLA-DRB1, HLA-DRB5, LNPEP, NCF1, PNKD, PSMB9, TAP1* |
| GO:0002819 | Regulation Of Adaptive Immune Response | 2.91E-07 | 2.12E-05 | 0.08 | *FADD, FCER1G, HLA-B, IL23A, IL27RA, IL4R, IRF1, LTA, NDFIP1, SIRT1, SMAD7, STAT6* |
| GO:0042088 | T-Helper 1 Type Immune Response | 1.02E-06 | 7.32E-05 | 0.16 | *HLA-DRB1, IL18R1, IL23A, IL27RA, IL4R, STAT6, TLR6* |
| GO:0002474 | Antigen Processing And Presentation Of Peptide Antigen Via Mhc Class I | 4.47E-06 | 3.17E-04 | 0.09 | *FCER1G, FCGR1B, HLA-B, HLA-C, LNPEP, NCF1, PNKD, PSMB9, TAP1* |
| GO:0002822 | Regulation Of Adaptive Immune Response Based On Somatic Recombination Of Immune Receptors Built From Immunoglobulin Superfamily Domains | 7.31E-06 | 5.11E-04 | 0.08 | *FADD, FCER1G, HLA-B, IL23A, IL27RA, IL4R, LTA, NDFIP1, SMAD7, STAT6* |
| GO:0002706 | Regulation Of Lymphocyte Mediated Immunity | 7.81E-06 | 5.39E-04 | 0.07 | *FADD, FCER1G, HLA-B, IL23A, IL27RA, LTA, MICA, NDFIP1, SMAD7, STAT6* |
| GO:0002821 | Positive Regulation Of Adaptive Immune Response | 1.53E-05 | 1.04E-03 | 0.09 | *FADD, FCER1G, HLA-B, IL23A, IL27RA, LTA, SIRT1, STAT6* |
| GO:0002460 | Adaptive Immune Response Based On Somatic Recombination Of Immune Receptors Built From Immunoglobulin Superfamily Domains | 2.60E-05 | 1.74E-03 | 0.04 | *FADD, FCER1G, GNL1, HLA-B, HLA-DRB1, IL18R1, IL23A, IL27RA, IL4R, LTA, MICA, NDFIP1, SMAD7, STAT6, TLR6* |
| GO:0060333 | Interferon-Gamma-Mediated Signaling Pathway | 3.13E-05 | 2.06E-03 | 0.08 | *CIITA, FCGR1B, HLA-B, HLA-C, HLA-DQA1, HLA-DRB1, HLA-DRB5, IRF1* |
| GO:0002285 | Lymphocyte Activation Involved In Immune Response | 4.76E-05 | 3.09E-03 | 0.06 | *DOCK10, F2RL1, FCER1G, IL18R1, IL23A, IL27RA, IL4R, NDFIP1, SMAD7, STAT6* |
| GO:0050777 | Negative Regulation Of Immune Response | 9.00E-05 | 5.76E-03 | 0.06 | *HLA-B, HLA-DRB1, IL27RA, IL4R, LILRB2, MICA, NDFIP1, SMAD7, STAT6* |
| GO:0046632 | Alpha-Beta T Cell Differentiation | 1.75E-04 | 1.10E-02 | 0.08 | *GPR18, IL18R1, IL23A, IL4R, IRF1, SMAD7, STAT6* |
| GO:0002699 | Positive Regulation Of Immune Effector Process | 1.99E-04 | 1.23E-02 | 0.05 | *F2RL1, FADD, FCER1G, FES, GAB2, HLA-B, IL23A, IL4R, LTA, STAT6* |
| GO:0002286 | T Cell Activation Involved In Immune Response | 2.14E-04 | 1.30E-02 | 0.07 | *F2RL1, FCER1G, IL18R1, IL23A, IL4R, SMAD7, STAT6* |
| GO:0002480 | Antigen Processing And Presentation Of Exogenous Peptide Antigen Via Mhc Class I, Tap-Independent | 2.23E-04 | 1.34E-02 | 0.30 | *HLA-B, HLA-C, LNPEP* |
| GO:0043300 | Regulation Of Leukocyte Degranulation | 2.48E-04 | 1.46E-02 | 0.11 | *F2RL1, FCER1G, FES, GAB2, IL4R* |
| GO:0043280 | Positive Regulation Of Cysteine-Type Endopeptidase Activity Involved In Apoptotic Process | 3.03E-04 | 1.76E-02 | 0.06 | *ANP32B, DAP, FADD, GRAMD4, IFT57, RET, RPS27L, SIRT1* |
| GO:0002886 | Regulation Of Myeloid Leukocyte Mediated Immunity | 3.05E-04 | 1.74E-02 | 0.11 | *F2RL1, FCER1G, FES, GAB2, IL4R* |
| GO:0019885 | Antigen Processing And Presentation Of Endogenous Peptide Antigen Via Mhc Class I | 4.01E-04 | 2.25E-02 | 0.25 | *HLA-B, HLA-C, TAP1* |
| GO:0002889 | Regulation Of Immunoglobulin Mediated Immune Response | 4.49E-04 | 2.47E-02 | 0.10 | *FCER1G, IL27RA, LTA, NDFIP1, STAT6* |
| GO:0032609 | Interferon-Gamma Production | 4.92E-04 | 2.66E-02 | 0.06 | *F2RL1, FADD, HLA-DRB1, IL18R1, IL23A, IL27RA, LTA* |
| GO:0002479 | Antigen Processing And Presentation Of Exogenous Peptide Antigen Via Mhc Class I, Tap-Dependent | 5.22E-04 | 2.77E-02 | 0.08 | *FCGR1B, HLA-B, HLA-C, NCF1, PSMB9, TAP1* |
| GO:0002828 | Regulation Of Type 2 Immune Response | 5.95E-04 | 3.09E-02 | 0.13 | *IL27RA, IL4R, NDFIP1, STAT6* |
| GO:0002294 | Cd4-Positive, Alpha-Beta T Cell Differentiation Involved In Immune Response | 6.38E-04 | 3.25E-02 | 0.09 | *IL18R1, IL23A, IL4R, SMAD7, STAT6* |
| GO:0009437 | Carnitine Metabolic Process | 6.52E-04 | 3.26E-02 | 0.21 | *CPT1A, SLC22A4, SLC22A5* |
| GO:0001913 | T Cell Mediated Cytotoxicity | 6.73E-04 | 3.30E-02 | 0.13 | *FADD, HLA-B, IL23A, MICA* |
| GO:0002708 | Positive Regulation Of Lymphocyte Mediated Immunity | 7.21E-04 | 3.46E-02 | 0.07 | *FADD, FCER1G, HLA-B, IL23A, LTA, STAT6* |
| GO:0030217 | T Cell Differentiation | 7.52E-04 | 3.53E-02 | 0.04 | *FADD, FCER1G, GPR18, IL18R1, IL23A, IL4R, IRF1, LILRB2, SMAD7, STAT6* |
| GO:0002456 | T Cell Mediated Immunity | 9.19E-04 | 4.23E-02 | 0.07 | *FADD, GNL1, HLA-B, IL23A, MICA, SMAD7* |
| GO:0016045 | Detection Of Bacterium | 9.84E-04 | 4.43E-02 | 0.19 | *HLA-B, HLA-DRB1, TLR6* |
| GO:0001910 | Regulation Of Leukocyte Mediated Cytotoxicity | 1.03E-03 | 4.51E-02 | 0.08 | *F2RL1, FADD, HLA-B, IL23A, MICA* |

**Supplemental Table S7. Multiple eSNPs identified in 11 candidate genes implicated in moderate-to-severe asthma**

| **Gene name** | **SNP ID** | **Chromosome** | **Position** | **Proximity** | **eQTL P values** | **GWAS P values** | **LBF** | **Gene resources** |
| --- | --- | --- | --- | --- | --- | --- | --- | --- |
| *HLA-DRB5* | rs9266455 | 6 | 31446364 | trans | 1.41E-18 | 2.38E-05 | 4.29 | Zeller eQTL |
| *HLA-DRB5* | rs9272346 | 6 | 32712350 | cis | 2.15E-100 | 8.77E-18 | 6.51 | Zeller eQTL |
| *HLA-DRB5* | rs2802329 | 1 | 26338776 | trans | 1.00E-05 | 2.32E-02 | 0.09 | Duan eQTL |
| *HLA-DRB5* | rs12120763 | 1 | 184408459 | trans | 1.00E-05 | 2.79E-02 | -0.07 | Duan eQTL |
| *HLA-DRB5* | rs973392 | 2 | 45213175 | trans | 1.00E-05 | 3.30E-02 | 0.00 | Duan eQTL |
| *HLA-DRB5* | rs3117572 | 6 | 31825671 | cis | 3.00E-05 | 3.40E-04 | 2.56 | Duan eQTL |
| *HLA-DRB5* | rs3830058 | 6 | 32741988 | cis | 2.00E-19 | 9.44E-14 | 6.62 | Duan eQTL |
| *HLA-DRB5* | rs6942478 | 7 | 81240410 | trans | 1.00E-05 | 4.12E-02 | 0.09 | Duan eQTL |
| *HLA-DRB5* | rs1529712 | 19 | 44358807 | trans | 1.00E-05 | 1.75E-03 | 1.31 | Duan eQTL |
| *HLA-DRB5* | rs9609057 | 22 | 29438617 | trans | 1.00E-05 | 2.36E-03 | 1.04 | Duan eQTL |
| *HLA-DRB5* | rs9267658 | 6 | 31953964 | cis | 6.50E-06 | 2.69E-03 | 2.81 | Dixon eQTL |
| *HLA-DRB1* | rs9266455 | 6 | 31446364 | trans | 5.93E-26 | 2.38E-05 | 4.07 | Zeller eQTL |
| *HLA-DRB1* | rs9272346 | 6 | 32712350 | cis | 2.46E-128 | 8.77E-18 | 6.22 | Zeller eQTL |
| *HLA-DRB1* | rs2802329 | 1 | 26338776 | trans | 1.00E-05 | 2.32E-02 | 0.09 | Duan eQTL |
| *HLA-DRB1* | rs12120763 | 1 | 184408459 | trans | 1.00E-05 | 2.79E-02 | -0.07 | Duan eQTL |
| *HLA-DRB1* | rs973392 | 2 | 45213175 | trans | 1.00E-05 | 3.30E-02 | 0.00 | Duan eQTL |
| *HLA-DRB1* | rs3117572 | 6 | 31825671 | cis | 3.00E-05 | 3.40E-04 | 2.56 | Duan eQTL |
| *HLA-DRB1* | rs3830058 | 6 | 32741988 | cis | 2.00E-19 | 9.44E-14 | 6.62 | Duan eQTL |
| *HLA-DRB1* | rs6942478 | 7 | 81240410 | trans | 1.00E-05 | 4.12E-02 | 0.09 | Duan eQTL |
| *HLA-DRB1* | rs1529712 | 19 | 44358807 | trans | 1.00E-05 | 1.75E-03 | 1.31 | Duan eQTL |
| *HLA-DRB1* | rs9609057 | 22 | 29438617 | trans | 1.00E-05 | 2.36E-03 | 1.04 | Duan eQTL |
| *HLA-DRB1* | rs750764 | 2 | 220489250 | trans | 8.90E-06 | 5.22E-03 | 0.73 | Dixon eQTL |
| *HLA-DRB1* | rs2516049 | 6 | 32678378 | cis | 5.70E-25 | 4.63E-08 | 5.82 | Dixon eQTL |
| *HLA-DRB1* | rs2448001 | 11 | 27350389 | trans | 5.10E-06 | 5.60E-03 | 0.96 | Dixon eQTL |
| *GNGT2* | rs17637472 | 17 | 44816432 | cis | 2.98E-08 | 3.40E-08 | 6.63 | Zeller eQTL |
| *GNGT2* | rs11265180 | 1 | 157610272 | trans | 6.00E-06 | 1.99E-03 | 1.32 | Duan eQTL |
| *GNGT2* | rs1867087 | 17 | 44795419 | cis | 1.00E-04 | 1.84E-05 | 4.51 | Dixon eQTL |
| *HLA-DQA1* | rs17426593 | 6 | 32716055 | cis | 2.29E-102 | 2.38E-13 | 7.20 | Zeller eQTL |
| *HLA-DQA1* | rs156650 | 1 | 44828880 | trans | 2.00E-07 | 8.35E-03 | 1.24 | Duan eQTL |
| *HLA-DQA1* | rs7527372 | 1 | 216353958 | trans | 5.00E-06 | 3.02E-02 | 0.21 | Duan eQTL |
| *HLA-DQA1* | rs12477083 | 2 | 128759109 | trans | 1.00E-05 | 3.65E-02 | 0.11 | Duan eQTL |
| *HLA-DQA1* | rs3130614 | 6 | 31584437 | trans | 2.00E-06 | 1.42E-03 | 1.30 | Duan eQTL |
| *HLA-DQA1* | rs9272346 | 6 | 32712350 | cis | 2.00E-09 | 8.77E-18 | 6.74 | Duan eQTL |
| *HLA-DQA1* | rs11768402 | 7 | 94998789 | trans | 4.00E-06 | 4.84E-02 | 0.04 | Duan eQTL |
| *HLA-DQA1* | rs6578882 | 11 | 7555967 | trans | 4.00E-06 | 1.16E-02 | 0.51 | Duan eQTL |
| *HLA-DQA1* | rs564404 | 11 | 58574201 | trans | 7.00E-06 | 1.25E-02 | 0.30 | Duan eQTL |
| *HLA-DQA1* | rs1522374 | 3 | 153718220 | trans | 2.50E-06 | 3.07E-04 | 3.33 | Dixon eQTL |
| *HLA-DQA1* | rs3130981 | 6 | 31191792 | trans | 2.80E-07 | 4.93E-02 | 0.00 | Dixon eQTL |
| *HLA-DQA1* | rs9267658 | 6 | 31953964 | cis | 1.30E-09 | 2.69E-03 | 2.40 | Dixon eQTL |
| *HLA-DQA1* | rs2516049 | 6 | 32678378 | cis | 4.20E-31 | 4.63E-08 | 5.81 | Dixon eQTL |
| *SLC22A5* | rs4371745 | 5 | 131807854 | cis | 7.16E-18 | 8.33E-09 | 5.19 | Zeller eQTL |
| *SLC22A5* | rs6595948 | 5 | 129697436 | trans | 1.00E-05 | 1.21E-02 | 0.36 | Duan eQTL |
| *SLC22A5* | rs2631360 | 5 | 131735328 | cis | 1.00E-04 | 1.77E-08 | 5.16 | Duan eQTL |
| *SLC22A5* | rs6717510 | 2 | 224796073 | trans | 7.30E-06 | 1.83E-02 | 0.30 | Dixon eQTL |
| *SLC22A5* | rs1812310 | 4 | 5272479 | trans | 6.60E-06 | 4.41E-02 | 0.14 | Dixon eQTL |
| *SLC22A5* | rs11950562 | 5 | 131680428 | cis | 1.80E-08 | 9.96E-08 | 4.99 | Dixon eQTL |
| *STAT6* | rs12368672 | 12 | 55798737 | cis | 3.38E-77 | 3.13E-11 | 4.56 | Zeller eQTL |
| *STAT6* | rs6807471 | 3 | 31849018 | trans | 1.00E-05 | 8.38E-03 | 0.50 | Duan eQTL |
| *STAT6* | rs10003541 | 4 | 142100509 | trans | 3.00E-06 | 1.80E-02 | 0.49 | Duan eQTL |
| *STAT6* | rs324015 | 12 | 55776367 | cis | 1.30E-04 | 1.33E-03 | 1.42 | Dixon eQTL |
| *MPI* | rs13148144 | 4 | 139459922 | trans | 4.71E-06 | 4.77E-02 | 0.09 | Zeller eQTL |
| *MPI* | rs1133323 | 15 | 72999278 | cis | 8.93E-10 | 2.43E-04 | 4.08 | Zeller eQTL |
| *MPI* | rs11072518 | 15 | 73021663 | cis | 8.00E-05 | 3.06E-03 | 0.83 | Duan eQTL |
| *MPI* | rs4886648 | 15 | 73095890 | cis | 4.00E-04 | 3.62E-04 | 1.17 | Dixon eQTL |
| *TLR6* | rs11466640 | 4 | 38455298 | cis | 1.64E-07 | 2.00E-03 | 2.55 | Zeller eQTL |
| *TLR6* | rs11130280 | 3 | 51186315 | trans | 5.00E-06 | 1.83E-02 | 0.28 | Duan eQTL |
| *TLR6* | rs5743592 | 4 | 38479458 | cis | 3.00E-06 | 2.49E-03 | 2.18 | Duan eQTL |
| *TLR6* | rs5743595 | 4 | 38479039 | cis | 4.50E-07 | 2.61E-03 | 2.17 | Dixon eQTL |
| *DECR2* | rs1698231 | 16 | 407586 | cis | 8.57E-39 | 2.42E-03 | 1.64 | Zeller eQTL |
| *DECR2* | rs17769811 | 12 | 3549815 | trans | 3.00E-06 | 2.01E-03 | 1.68 | Duan eQTL |
| *DECR2* | rs1698231 | 16 | 407586 | cis | 8.00E-06 | 2.42E-03 | 2.73 | Duan eQTL |
| *DECR2* | rs1204504 | 16 | 401763 | cis | 6.00E-05 | 2.44E-03 | 0.69 | Dixon eQTL |
| *LNPEP* | rs3909451 | 5 | 96320877 | cis | 4.79E-07 | 1.30E-02 | 1.23 | Zeller eQTL |
| *LNPEP* | rs1608927 | 1 | 72997067 | trans | 9.00E-06 | 3.15E-02 | -0.28 | Duan eQTL |
| *LNPEP* | rs2323571 | 3 | 74701931 | trans | 7.00E-06 | 7.36E-04 | 1.69 | Duan eQTL |
| *LNPEP* | rs382424 | 10 | 64159780 | trans | 6.00E-06 | 2.48E-02 | 0.22 | Duan eQTL |
| *LNPEP* | rs1104906 | 10 | 80662663 | trans | 1.00E-05 | 3.74E-03 | 0.87 | Duan eQTL |
| *LNPEP* | rs2518348 | 11 | 111601847 | trans | 9.00E-06 | 4.35E-02 | 0.05 | Duan eQTL |
| *LNPEP* | rs248186 | 5 | 21163415 | trans | 6.90E-06 | 9.85E-03 | 0.53 | Dixon eQTL |
| *LNPEP* | rs9569076 | 13 | 54125829 | trans | 9.80E-06 | 2.75E-02 | 0.22 | Dixon eQTL |
| *LNPEP* | rs11864608 | 16 | 15823628 | trans | 2.80E-06 | 4.47E-04 | 3.12 | Dixon eQTL |
| *TTC19* | rs3785631 | 17 | 15886671 | cis | 1.79E-17 | 6.37E-03 | 1.67 | Zeller eQTL |
| *TTC19* | rs7990880 | 13 | 60086872 | trans | 1.00E-05 | 3.82E-02 | 0.06 | Duan eQTL |
| *TTC19* | rs1160109 | 17 | 15973857 | cis | 1.00E-06 | 6.19E-03 | 1.63 | Duan eQTL |
| *TTC19* | rs1485006 | 4 | 94617011 | trans | 9.60E-06 | 2.24E-02 | 0.20 | Dixon eQTL |
| *TTC19* | rs4245210 | 11 | 119336983 | trans | 8.10E-06 | 9.82E-03 | 0.47 | Dixon eQTL |
| *TTC19* | rs1446313 | 15 | 75262435 | trans | 1.80E-06 | 4.51E-02 | 0.17 | Dixon eQTL |
| *TTC19* | rs3785631 | 17 | 15886671 | cis | 1.90E-20 | 6.37E-03 | 1.51 | Dixon eQTL |

**Supplemental Table S8. Additional evidence of eQTL findings for *GNGT2* gene**

| **rs# of eSNP** | **Traits** | **Tissues** | **P values** | **References (PMID)** |
| --- | --- | --- | --- | --- |
| rs17637472 | Systematic identification of trans eQTLs as putative drivers of known disease associations | Whole blood | 2.13×10^-52^ | 24013639 |
| rs1867087 | Systematic identification of trans eQTLs as putative drivers of known disease associations | Whole blood | 3.79×10^-22^ | 24013639 |
